# Lignin-derived guaiacols as platform chemicals for the modular synthesis of 1,2,3,4-tetrahydroquinolines and benzomorpholines†

**DOI:** 10.1039/d5su00151j

**Published:** 2025-07-02

**Authors:** Antonio A. Castillo-Garcia, Jörg Haupenthal, Anna K. H. Hirsch, Katalin Barta

**Affiliations:** a Institute of Chemistry, University of Graz Heinrichstrasse 28/II A-8010 Graz Austria katalin.barta@uni-graz.at; b Stratingh Institute for Chemistry, University of Groningen Nijenborgh 4, 9747 AG Groningen The Netherlands; c Helmholtz Institute for Pharmaceutical Research Saarland (HIPS)–Helmholtz Centre for Infection Research (HZI) Campus Building E8.1 66123 Saarbrücken Germany; d PharmaScienceHub Campus Building A2.3 66123 Saarbrücken Germany; e Saarland University, Department of Pharmacy Campus Building E8.1 66123 Saarbrücken Germany

## Abstract

Reductive catalytic fractionation (RCF) has emerged as a centrally important method in modern biorefining, delivering well-defined aromatic platform chemicals from lignin with high selectivity. To establish attractive future biorefinery schemes, urgent attention needs to be devoted to the development of sustainable catalytic methods for the downstream conversion of these aromatic platform chemicals. In this regard, the efficient production of structurally complex, biologically active amines with high atom and step economy represents an attractive goal. Herein, we describe the development of novel catalytic pathways for converting prominent lignin-derived guaiacols that originated during RCF processing into different series of six-membered N-heterocycles, applying hydrogen borrowing amination and C–N cross coupling as key catalytic steps. Specifically, 4-propanol guaiacol (1G) was converted into 1,2,3,4-tetrahydroquinolines 1Gd_n_, whereas the formation of benzomorpholines 2–3Gd_n_ from 4-propyl guaiacol (2G) and 4-ethyl guaiacol (3G) was achieved. The biological activity of the developed compound libraries was evaluated in terms of anticancer activity using human HepG2 cells, which displayed promising activity in several examples.

## Abstract 2

The development of novel catalytic pathways for converting lignin-derived guaiacols, into biologically active 1,2,3,4-tetrahydroquinolines and benzomorpholines by applying hydrogen borrowing amination and C–N cross coupling as key catalytic steps.

## Graphical abstract

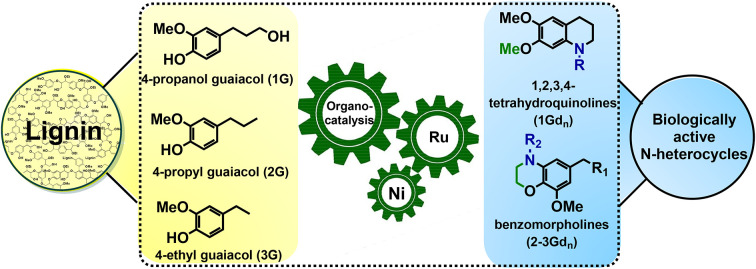

**Sustainability spotlight**

The valorization of lignin-derived building blocks into N-heterocycles with potential biological activity employing catalytic methods and avoiding the production of hazardous waste aims to contribute to the development of environmentally friendly alternatives for the sustainable manufacture of pharmaceuticals in accordance with the UN SDGs-2030, GOAL-3 (Good Health and Well-being).

## 1 Introduction

Considered the vastest source of renewable aromatics, lignin is the most promising non-fossil carbon feedstock for the production of aromatic chemicals.^1,2^ The aromatic core and intrinsic oxygenated functionalities of lignin and its subunits are commonly found in biologically active natural products^3^ and provide tremendous opportunities for further functionalization with the identification of suitable synthetic and catalytic methods. In this context, the transformation of biomass resources into nitrogen-containing chemicals^4–7^ has recently gained attention for the construction of biologically active cores.^8,9^ Specifically, N-heterocycles are privileged scaffolds given their ubiquity in pharmaceuticals.^10,11^ The key challenge in this regard is the identification of specific synthetic strategies and the development of appropriate catalytic methods to accomplish the formation of (typically) difficult-to-construct N-heterocycles with high atom economy and good step efficiency, starting from lignin-derived monomers.

**Fig. 1:**
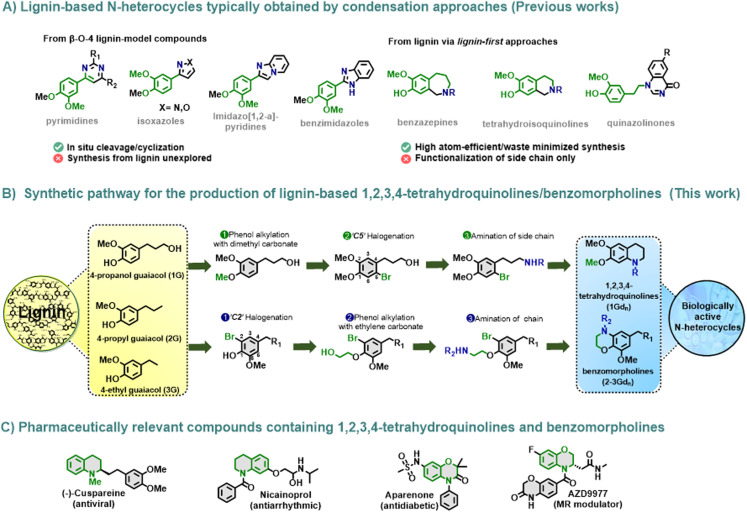
Synthetic strategy for obtaining lignin-based 1,2,3,4-tetrahydroquinolines 1Gd_n_ and benzomorpholines 2–3Gd_n_. (A) Previous reports on the formation of N-heterocycles containing lignin-derived motifs (highlighted in green). (B) Construction of 1,2,3-4-tetrahydroquinolines and benzomorpholines by modular functionalization of lignin-derived guaiacols. (C) Selected examples of pharmaceutically relevant compounds bearing the targeted N-heterocycles.

The aromatic monomers furnished upon lignin depolymerization inherently carry the structural features of the parent lignin, namely, recurrently *para*-substituted methoxylated phenols. The *para*-substituent depends mainly on the type of lignin depolymerization/stabilization method employed. Among the available depolymerization methods, reductive catalytic fractionation (RCF)^12^ displays various advantages, including its ability to efficiently convert lignin into a narrow product array and its high yield and efficiency. Various RCF strategies have been successfully developed over the past decade,^13–16^ obtaining monomer mixtures of 4-propanol guaiacol (1G) in most cases,^16*a*^ 4-propyl guaiacol (2G)^16*b*^ and 4-ethyl guaiacol (3G) in the case of softwood, and the respective syringol analogues in the case of hardwood. These monomers have found applications mostly in polymer synthesis.^17,18^ Targeting high-value pharmaceutically relevant compounds (Fig. 1A) has gained interest in recent years.^19,20^ Recent reports by Zhang, Li and co-workers attempted the synthesis of highly attractive N-heterocyclic scaffolds from β-O-4 lignin model compounds, following condensation approaches in a one-pot cleavage/cyclization fashion.^21^ Our group pioneered the modular synthesis of seven-membered tetrahydro-2-benzazepines from RCF-derived 4-propanol guaiacol (1G) in three waste-minimized steps from raw biomass, representing an important advancement in the search for greener routes for the construction of N-heterocycles from lignin, whereby ring formation was accomplished by Pictet–Spengler cyclization.^22*a*,*b*^ Later on, a similar strategy was applied to C2-monophenolics originating *via* ‘lignin-first’ Diol-Assisted Fractionation, leading to the discovery of a versatile library of highly bioactive N-heterocyclic compounds, including tetrahydroisoquinolines and quinazolinones.^22*c*^ In this contribution, we turn our attention to the formation of highly valuable 6-membered N-heterocycles. We envisage that upon judicious modification, the lignin-derived phenols (1–3G) can serve as suitable electrophiles for Ni-catalysed Csp_2_–N coupling reactions. Interestingly, limited examples involving C_sp^2^_–N bond formation are reported so far; most of them are mediated by Pd or Ir catalysis.^15,23–25^ Furthermore, the reactivity of these phenols can be enhanced by SEAr reactions, where regioselective pathways would be nonetheless advantageous.^26^ To address this, we propose that 1–3G can be selectively modified towards the formation of suitable derivatives for the construction of value-added N-heterocycles. More specifically, the halogenation and subsequent selective catalytic amination of the intrinsic propanol chain in 1G can lead to the construction of 6-membered 1,2,3,4-tetrahydroquinolines *via* C–N coupling.^27,28^ However, the *ortho*-halogenation of 2–3G, which contains a substantially less reactive aliphatic chain, followed by the formation of amino-alkyl derivatives *via* phenol hydroxyalkylation/amination, would allow for the formation of benzomorpholines (Fig. 1B).^29,30^ Overall, the methodology presented herein involved the construction of valuable bio-based nitrogen-containing scaffolds^31^ with broad pharmaceutical applications (Fig. 1C) using catalytic methods, benign solvents, and mild reaction conditions, and finally evaluated the biological activity of these novel lignin-derived N-heterocycles in terms of their anticancer activity on human HepG2 cells.

## 2 Experimental

### 2.1 Chemicals and materials

4-Propanol guaiacol (1G) was prepared according to the literature.^22^ 4-Propyl guaiacol (2G), 4-ethyl guaiacol (3G) and commercially available chemicals were acquired from Sigma-Aldrich and used as received without further purification. Shvo catalyst and bis(1,5-cyclooctadiene)nickel (0) were purchased from Strem chemicals. Cyclopentyl methyl ether (CPME, 99.9%, anhydrous) and toluene (99.9%, anhydrous) were purchased from Sigma-Aldrich.

#### 2.1.1 General procedure for the halogenation of lignin-derived guaiacols 1Ga and 2–3G

An oven-dried 10 mL glass vial was charged with 1Ga or 2–3G (0.5 mmol), NBS (265 mg, 1.5 mmol), C1 (3.5 mg, 0.025 mmol) and anhydrous toluene (5 mL). Then, the mixture was stirred at 0 °C for 0.5 h. After completion of the reaction, the mixture was quenched with NaHCO_3_ (5 mL) and extracted with EtOAc (3 × 5 mL). The organic phase was washed with water (2 × 10 mL) and saturated brine (2 × 10 mL). Finally, the combined organic phase was dried over Na_2_SO_4_, and a small aliquot (0.3 mL) was analysed by applying GC-MS/GC-FID to monitor the product formation.

#### 2.1.2 General procedure for the alkylation of 1G with dimethyl carbonate

The alkylation of 1G was carried out as previously reported.^32^ Step 1: A 20 mL oven-dried microwave vial equipped with a stirring bar was charged with 1G (364 mg, 2 mmol), K_2_CO_3_ (2.7 mg, 0.02 mmol) and dimethyl carbonate (1.8 g, 20 mmol). Afterwards, the vial was capped, placed into a heating block at 160 °C and stirred for 16 h. Then, the mixture was diluted in EtOAc (2 mL), and the solution was filtered and concentrated under reduced pressure. Step 2: The product obtained from Step 1 was dissolved in MeOH/H_2_O (5 : 1, 2.5 mL), and NaOH (54 mg, 1.3 mmol) was added to the mixture. The mixture was stirred at 40 °C for 4 h. After the reaction was completed, the mixture was filtered and concentrated under a vacuum. The residue was then dissolved in Et_2_O (10 mL) and washed with brine (2 × 10 mL). Finally, the organic phase was dried over anhydrous MgSO_4_, and the solvent was evaporated under reduced pressure.

#### 2.1.3 General procedure for alkylation of 2–3Ga with ethylene carbonate

A 20 mL oven-dried microwave vial equipped with a stirring bar was charged with 2–3Ga (1.0 mmol), ethylene carbonate (105.6 mg, 1.2 mmol), tetrabutylammonium fluoride (13.0 mg, 0.05 mmol) and DMF (0.2 mL). Then, the vial was capped and placed into a heating block at 190 °C and stirred for 1 h. After completion of the reaction, the mixture was diluted in MeOH (2 mL) and filtered with a PTFE septum. Then, the solvent was removed under reduced pressure, and the product was isolated by flash chromatography using a mixture of ethyl acetate/pentane as an eluent.

#### 2.1.4 General procedure for Ru-catalyzed N-alkylation of anilines with 1–3Gb

An oven-dried Schlenk tube equipped with a stirring bar was charged with 1–3Gb (0.26 mmol), amine (0.2 mmol), Shvo catalyst (8.5 mg, 0.008 mmol) and cyclopentyl methylether (CPME, 1.5 mL). Then, the Schlenk tube was subsequently connected to an argon line, and vacuum-argon exchange was performed three times. The Schlenk tube was capped, and the mixture was rapidly stirred at room temperature for 1 min, then placed into a pre-heated oil bath at 130 °C and stirred for 16 h. After completion, the reaction mixture was cooled to room temperature, and a small aliquot (0.3 mL) was analyzed using GC-FID and GC-MS to determine the conversion and yield. GC-FID analysis was carried out based on the calculation of response factors *via* calibration using 3,5-dimethyl phenol as an internal standard.

#### 2.1.5 General procedure for Ni-catalysed intramolecular C–N coupling

An oven-dried Schlenk tube equipped with a stirring bar was charged with *N*-alkylated derivative 1–3Gc_n_ (0.1 mmol), NaOtBu (15 mg, 0.15 mmol), bis(1,5-cyclooctadiene)nickel(0) (1.4 mg, 0.005 mmol), IPr·HCl (2.2 mg, 0.005 mmol) and toluene (1.5 mL). Then, the Schlenk tube was subsequently connected to an argon line; toluene was added under an argon stream, and vacuum-argon exchange was performed three times. The Schlenk tube was capped, and the mixture was rapidly stirred at room temperature for 1 min, then placed into a pre-heated oil bath at 110 °C and stirred for 16 h. Finally, the reaction mixture was cooled to room temperature. Conversion and yield were measured by GC-FID based on the calculation of response factors *via* calibration using 3,5-dimethyl phenol as an internal standard, and the identification of products was carried out by GC-MS.

#### 2.1.6 Procedure for cytotoxicity assay

The cultivation of the human hepatocellular carcinoma cell line HepG2 and the MTT assay to evaluate the metabolic activity of the novel compounds were performed at the Helmholtz Institute for Pharmaceutical Research Saarland (HIPS), Helmholtz Centre for Infection Research (HZI), following our previously described methodology.^33^

## 3 Results and discussion

### 3.1 Functionalization of lignin-derived guaiacols 1–3G by alkylation/halogenation reactions

**Scheme 1:**
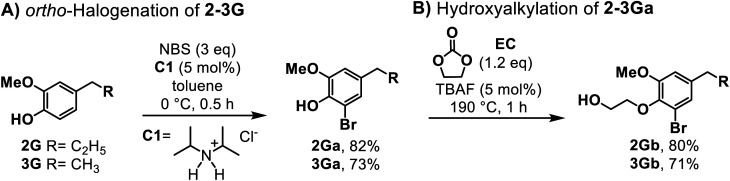
Synthesis of derivatives 2–3Gb. (A) *Ortho*-halogenation of 2-3G, (B) hydroxyalkylation of 2-3Ga (for experimental details, see ESI Section 2†).

We began our study with the selective *ortho*-halogenation of 4-ethyl guaiacol (3G) in toluene (Scheme 1). Although the use of *para*-substituted phenols is a significant advantage for this transformation, the inherent presence of two *ortho*, *para*-directing groups (–OH, –OMe) in such monomers might lead to the formation of polyhalogenated products.^34^ To increase regioselectivity, we employed the ammonium salt C1 as an organocatalyst, given its remarkable efficiency in *ortho*-halogenation reactions.^26,35^ In fact, the ability of C1 to simultaneously activate both phenol and *N*-bromosuccinimide resulted in high selectivity towards the *ortho*-brominated derivative when the reaction was conducted at 0 °C for 0.5 h, affording 3Ga in 73% isolated yield (see Table S1†). A drastic loss of selectivity was observed when the reaction was performed either without a catalyst at room temperature or with MeOH^36^ as a solvent (see Fig. S1–S4†). Similarly, the *ortho*-halogenation of 4-propyl guaiacol (2G) proceeded efficiently, affording 2Ga in 82% isolated yield. *Ortho*-brominated phenols are not only valuable intermediates for organic synthesis, but they are also frequently found in biologically active natural products.^37^ Hence, the selective synthesis of 2Ga and 3Ga provides an attractive route for the valorisation of these guaiacols, which have been scarcely utilized, especially for the construction of N-chemicals.^24^

**Scheme 2:**
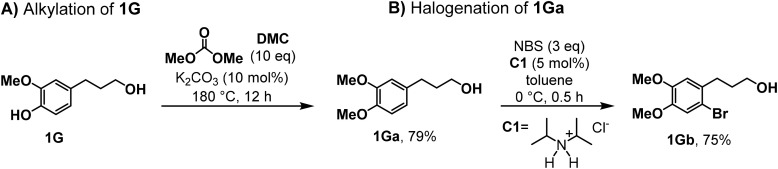
Synthesis of derivative 1Gb. (A) Alkylation of 1G. (B) Halogenation of 1Ga (for experimental details, see ESI Section 2†).

Furthermore, the phenol motif in the new brominated derivatives (2–3Ga) was submitted to hydroxyalkylation using ethylene carbonate (EC) as an alkylating agent. Recently, the use of cyclic carbonates has been explored as a green pathway to produce alkyl aryloxy motifs, which are recurrent intermediates in the production of pharmaceuticals and polymers.^38–41^ This reaction is usually conducted in the presence of either basic or phase-transfer catalysts; therefore, the hydroxyl alkylation of 2–3Ga was carried out using TBAF (5 mol%) as a catalyst at 190 °C for 1 h, affording good isolated yields in both cases (Scheme 1B). The formation of the lignin-based alkyl aryloxy intermediates (2–3Gb) with ethylene carbonate allows a more atom-efficient transformation, avoiding the use of alkyl halides, and enhances the reactivity of these monomers given the incorporation of the aliphatic alcohol, which can be diversified *via* C–N or C–C processes.^42^ However, the alkylation of 4-propanol guaiacol (1G) was successfully performed by employing dimethyl carbonate under low toxic, green methylating agent^43,44^ and catalytic amounts of K_2_CO_3_ under neat conditions (Scheme 2A). As expected, the methylation of the phenol moiety promoted the mono bromination of 1Ga in the *meta* position to the phenol moiety,^45^ and the synthesis of 1Gb was smoothly achieved under the same conditions as in the case of 2–3Ga, affording a 75% isolated yield (Scheme 2B). As mentioned before, the dual role of C1 significantly improves the selectivity of NBS towards the SEAr substitution; considering that this reagent has also been reported for the thiourea-mediated halogenation of aliphatic alcohols,^46^ no aliphatic bromination was satisfactorily observed in this case.

### 3.2 Alcohol amination of 1–3Gb*via* the hydrogen borrowing methodology

New lignin-based derivatives (1–3Gb) were then submitted to the amination step with diverse anilines *via* the borrowing hydrogen methodology.^47^ Previously, our group successfully explored the use of Shvo catalyst (C2) for the direct catalytic amination of different types of renewables, including the amination of 1G, under waste-free conditions.^22,48–50^ Interestingly, the efficiency of this system towards the *N*-alkylation of anilines and the tolerance to different substituents, including halogens, was observed. Although enormous efforts have been made to develop new catalytic methods for *N*-alkylation reactions employing earth-abundant metals,^51–55^ previous catalyst preparation or the use of additives, such as strong bases, which might affect the reactivity of 1–3Gb, is required. In contrast, the catalytic amination of aryloxy ethers, such as 2–3Gb, was achieved earlier using the [Ru(*p*-cymene)_2_Cl_2_]_2_/DPEPhos catalytic system under mild conditions.^56^ Therefore, we decided to utilize the commercially available Ru-based C2 for this study. Interestingly, the catalytic amination of 3Gb with *p*-anisidine (1) was readily achieved using cyclopentyl methyl ether (CPME) as a bio-derived solvent and 4 mol% of C2 when the reaction was carried out at 130 °C (Table 1, entry 1). The importance of using a small excess of 3Gb was confirmed when the reaction was performed with a stoichiometric ratio, obtaining a significantly lower yield (entry 4). Although C2 has shown exceptional activity even at low concentrations,^57^ only 15% conversion was observed when *N*-alkylation was performed employing 1 mol% of catalyst (entry 5).

**Table 1:** Catalytic alcohol amination of 3Gb with *para*-methoxy aniline (1)^a^

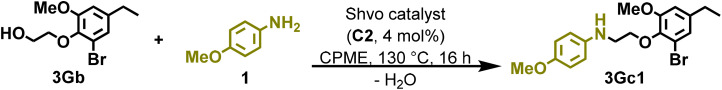		
Entry	Deviation from standard conditions	3Gc1^b^(%)
1	None	90 (77)^c^
2	*T* = 120 °C	11
3	*t* = 8 h	38
4	1 eq. of 3Gb	33
5	1 mol% of Shvo's catalyst	14
6	Toluene instead of CPME	88
7	2Gb instead of 3Gb	82^d^

a General reaction conditions: 3Gb (0.26 mmol), 1 (0.2 mmol), Shvo catalyst (0.008 mmol), CPME (2 mL), 130 °C, 20 h.

b Determined by GC-FID analysis using 3,5-dimethyl phenol as internal standard.

c Isolated yield.

d 2Gc1 as a product.

**Fig. 2:**
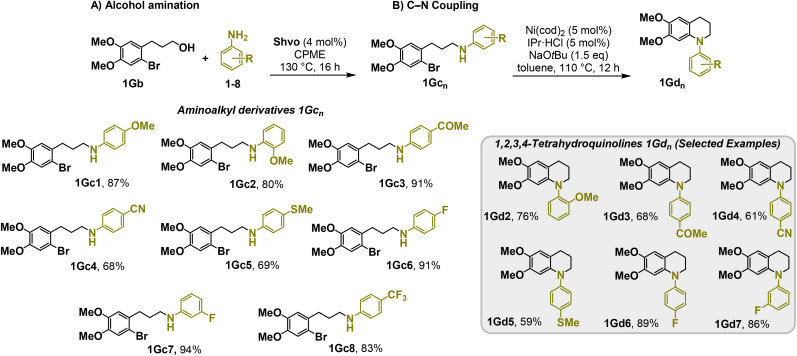
Construction of lignin-based 1,2,3,4-tetrahydroquinolines 1Gd_n_. (A) (Alcohol amination): 1Gb (0.26 mmol), 1–8 (0.2 mmol), Shvo catalyst (0.008 mmol), CPME (1.5 mL), 130 °C, 16 h. Isolated yields are shown. (B) (C–N coupling): 1Gc_n_ (0.1 mmol), Ni(cod)_2_ (0.005 mmol), IPr·HCl (0.005 mmol), NaOtBu (0.15 mmol), toluene (1.5 mL), 12 h. Isolated yields are shown.

**Fig. 3:**
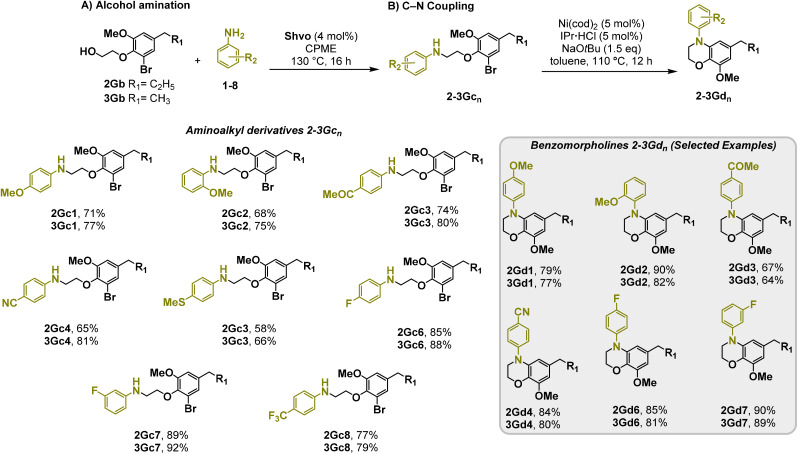
Synthesis of lignin-based benzomorpholines 2–3Gd_n_. (A) (Alcohol amination): 2–3Gb (0.26 mmol), 1–8 (0.2 mmol), Shvo catalyst (0.008 mmol), CPME (1.5 mL), 130 °C, 16 h. Isolated yields are shown. (B) (C–N coupling): 2–3Gc_n_ (0.1 mmol), Ni(cod)_2_ (0.005 mmol), IPr·HCl (0.005 mmol), NaOtBu (0.15 mmol), toluene (1.5 mL), 12 h. Isolated yields are shown.

Moreover, we observed that the presence of the electron-withdrawing -Br substituent in the new derivatives 1–3Gb enhances the reactivity of such substrates compared to the non-halogenated analogue 3G–OH (see ESI Note 1†), where a significantly lower conversion was detected when the reaction with *p*-anisidine was performed under the same reaction conditions. Next, diverse substituted anilines were submitted to the *N*-alkylation reaction with 1–3Gb (Fig. 2A and 3A), where amino alkyl derivatives with electron-donating substituents, such as –OCH_3_ (1–3Gc1 and 1–3Gc2) or –SCH_3_ (1–3Gc5), were generally obtained in good yields. Additionally, derivatives bearing anilines with electron-withdrawing substituents, such as –F (1–3Gc6 and 1–3Gc7) or –CF_3_ (1–3Gc8), were efficiently afforded in good to excellent yields.

Further transformations can be made in the case of 1–3Gc3 and 1–3Gc4 where the anilines contain reducible functional groups. Interestingly, the preparation of *N*-(2-phenoxyethyl)-amines, such as 2–3Gc_n_, was previously achieved by reacting amines with carboxylic acids; however, these examples are either mediated by Pt catalysts^58^ or involve the use of higher temperatures^59^ or H_2_/hydrogen donors.^60–62^ Therefore, the method described herein undoubtedly provides a much milder and more modular pathway for the synthesis of this type of structure. Interestingly, the anticarcinogenic activity of similar *N*-(2-phenoxyethyl)-amines scaffolds in diverse cell lines has been recently reported^63^ because they are recognized as reactive oxygen species (ROS) capable of acting as DNA-modifying agents.^64^

### 3.3 Ni-mediated synthesis of 1,2,3,4-tetrahydroquinolines 1Gd_n_ and benzomorpholines 2–3Gd_n_ by intramolecular C–N coupling

After establishing a convenient method for the modular amination of lignin-derived guaiacols 1–3Gb_n_, we focused our attention on the intramolecular cyclization of the aminoalkyl derivatives 1–3Gc_n_. Inspired by the seminal work by Fort and coworkers,^65^ where the intramolecular amination of aryl chlorides catalyzed by the Ni(cod)_2_/SIPr system was efficiently performed. We focused our efforts on the application of Ni-based catalytic systems. Traditionally, the use of Ni catalysts with N-heterocyclic carbenes (NHC) has shown remarkable activity in forging C–N couplings. Therefore, we foresaw that the intramolecular cyclization of the 1–3Gc_n_ derivatives should be feasible. Gratifyingly, the construction of a series of novel lignin-based 1,2,3,4-tetrahydroquinolines (1Gd_n_) was conveniently performed by employing Ni(cod)_2_/IPr·HCl and NaOtBu (1.5 eq.) as bases in toluene under reflux for 16 h (Fig. 2B), providing a good alternative to access this building block from aminoalkyl derivatives, formerly attempted through a two-step functionalization of fossil-based styrenes mediated by Ti and Pd.^57^ Significant lower conversion was detected using other NHC-carbenes (IMes, SIPr, SIMes) (see Table S2,† entry 2). As expected, almost no conversion was observed when commercially available Ni(ii) precatalysts were utilized instead of Ni(cod)_2_ (Table S2,† entry 4). The effects of temperature and base were also studied when the reaction was carried out at a lower temperature (80 °C) or using Cs_2_CO_3_ as a base (1.5 eq.), observing a yield decrease in both cases (Table S2,† entry 5–6). Notably, the use of the aforementioned Ni systems has also been investigated in the catalytic C_sp^2^_–O bond activation of aryl-methyl ethers, where demethoxylated products are commonly obtained.^66^ In addition, the catalytic amination of aryl methyl ethers has also been reported under analogous reaction conditions.^67,68^ In this case, C_sp^2^_–O bond activation towards demethoxylation or amination reactions was not observed, keeping all the intrinsic lignin-derived functionalities intact, which is a beneficial factor in terms of biomass utilization efficiency (BUE).^12^ Similarly, the intramolecular cyclization of diverse amino alkyl derivatives 2–3Gc_n_ led to the formation of a series of lignin-based benzomorpholines 2–3Gd_n_ containing various substituents, affording moderate to good isolated yields (>60%) in all the cases (Fig. 3B); this process enables an “earth-metal-based” synthesis protocol of these building blocks, which is previously approached following Pd-based methods.^69^

### 3.4 Preliminary evaluation of the biological activity of 1–3Gd_n_ heterocycles

The biological activity of six-membered 1,2,3,4-tetrahydroquinolines and benzomorpholines has been explored over the years, identifying potential applications as antiviral, anticancer, or antimicrobial agents. For instance, these scaffolds have shown intriguing potential as RORγt agonists for the treatment of certain types of cancer.^28^ Therefore, as a preliminary study, the anticancer activity of the selected 1–3Gdn compounds was evaluated on the human hepatoma cell line HepG2 (Table 2). Overall, moderate inhibition of cell viability in the range of 60–70% at 100 μM was detected in the case of 1,2,3,4-tetrahydroquinolines 1Gdn. However, the influence of the substituents is more evident in the case of benzomorpholines 2–3Gd_n_, where the presence of potential reducible groups, such as –COMe (2–3Gd3) or –CN (2–3Gd4), enhances the inhibitory effect of these derivatives compared to other substituents. Based on these initial results, we deduce that further molecular design, optimization and development of the most promising inhibitors for anticancer drugs are necessary to enhance the activity of these new lignin-derived N-heterocycles.

**Table 2:** Inhibitory effects of compounds 1–3Gd_n_ on the viability of HepG2 cells treated with 100 μM of the indicated compounds^a^

*n* =	1	2	3	4	5	6	7
1Gd_n_		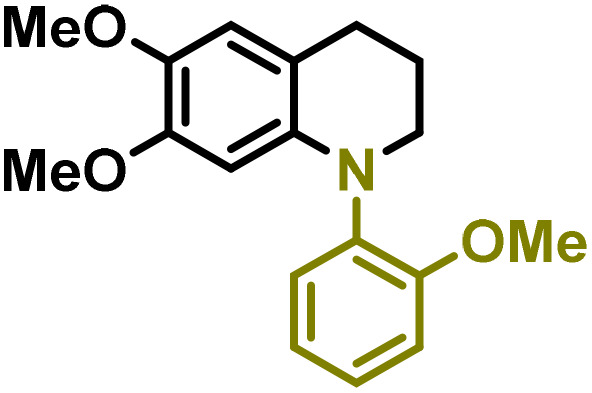	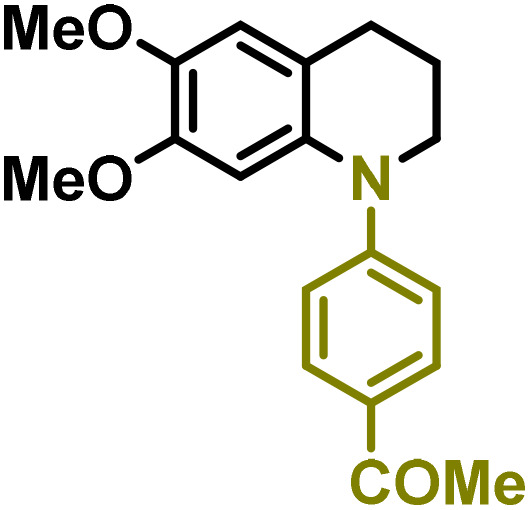	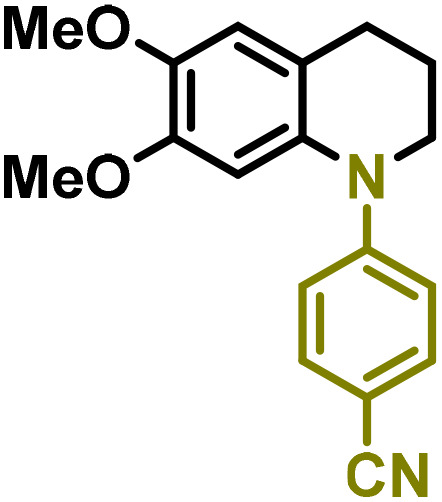	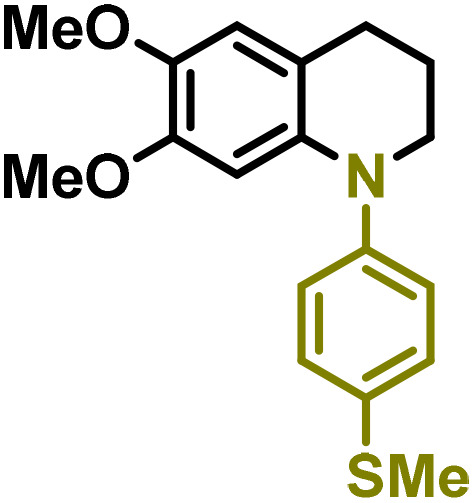	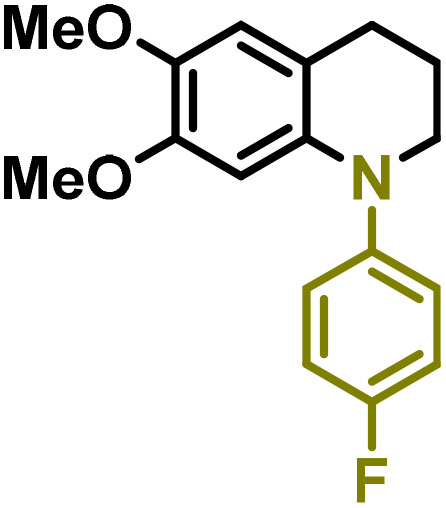	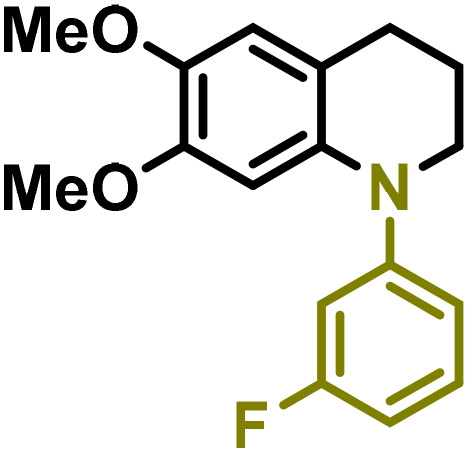
		66.4 ± 6.6	66.6 ± 5.4	64.4 ± 9.2	69.5 ± 4.6	69.2 ± 2.4	63.8 ± 0.3
2Gd_n_	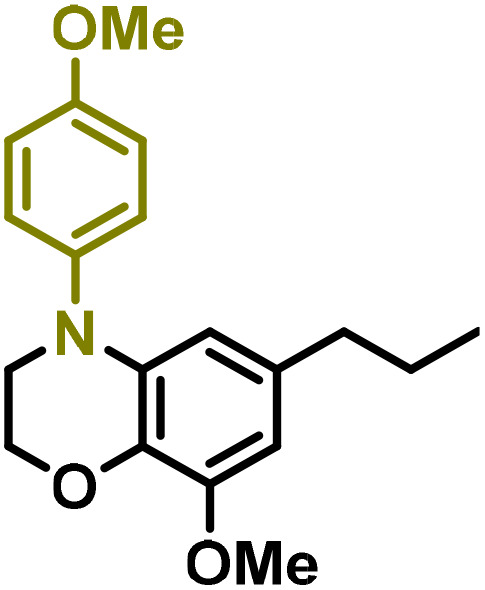	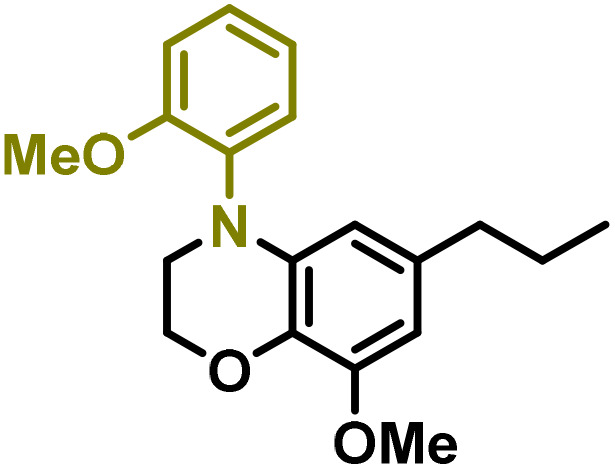	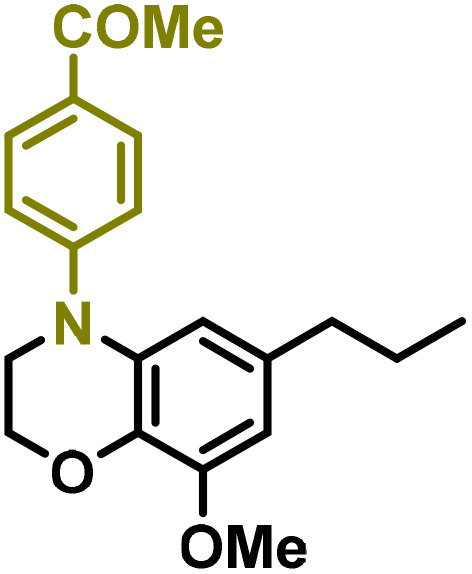	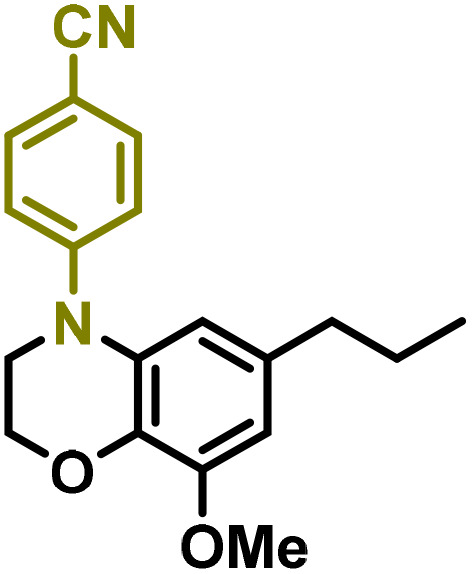		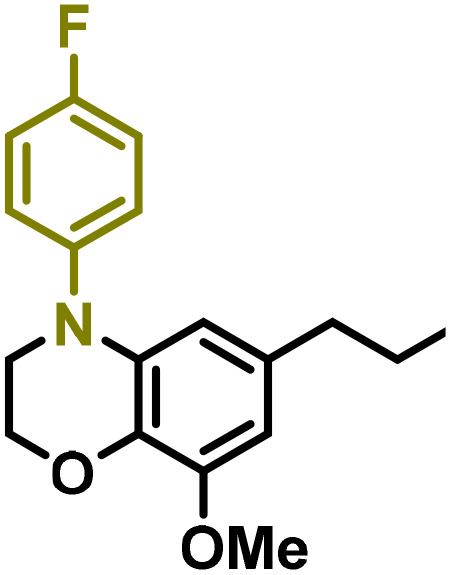	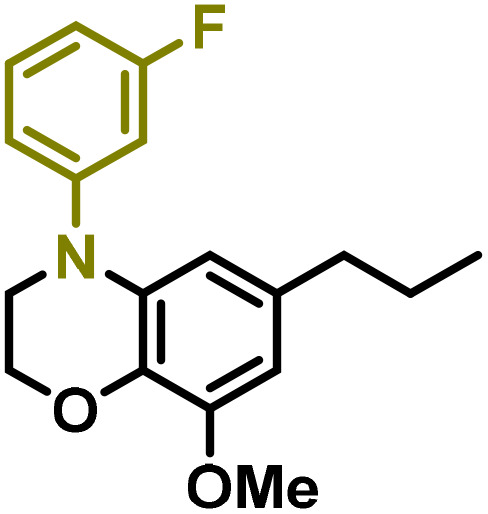
	57.7 ± 10.3	34.4 ± 12.5	77.3 ± 0.9	71.6 ± 3.5		39.0 ± 8.3	17.2 ± 10.7
3Gd_n_	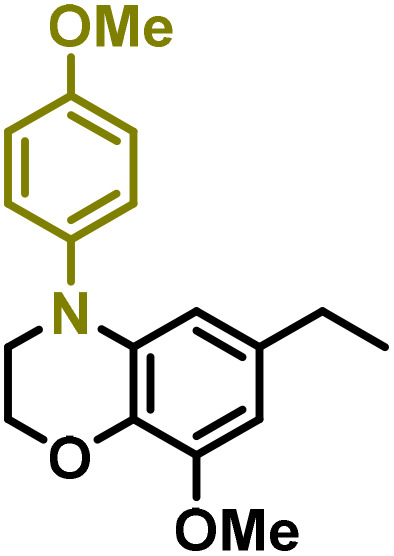	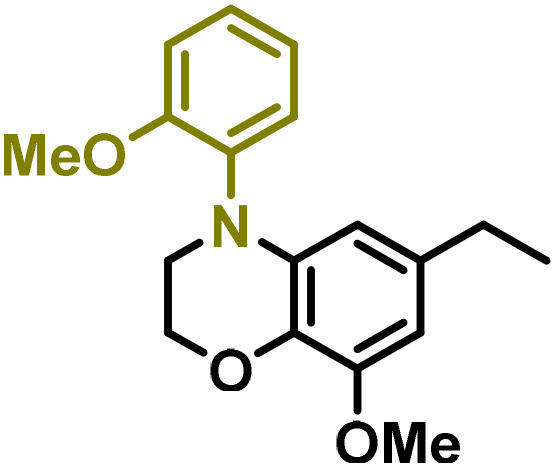	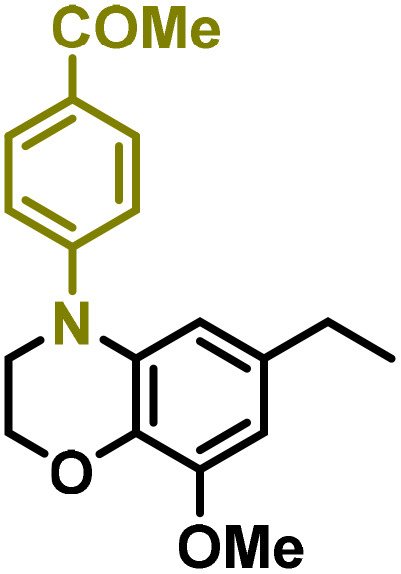	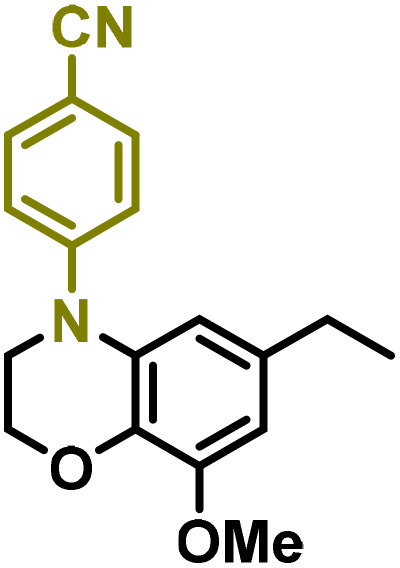		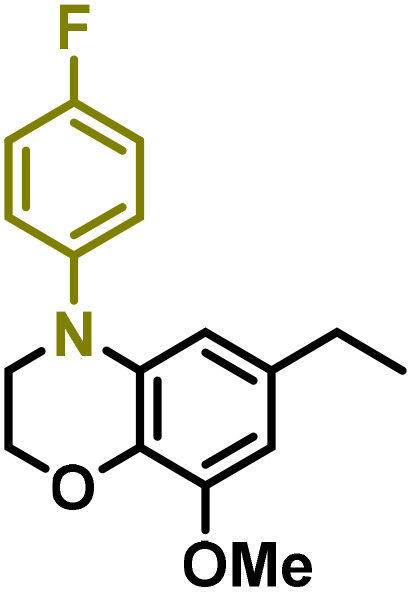	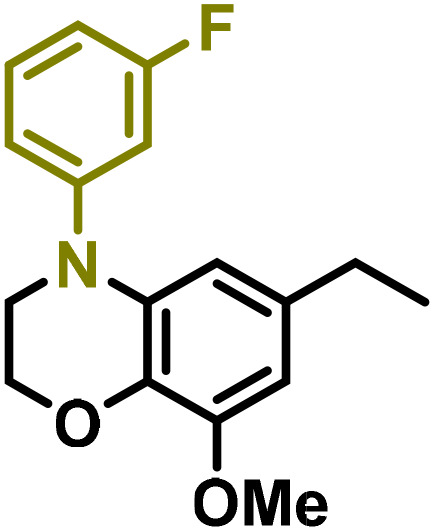
	35.4 ± 7.0	34.7 ± 10.8	63.8 ± 0.3	69.2 ± 4.9		54.4 ± 3.2	57.9 ± 3.2

a Percent (%) inhibition values and standard deviations from at least two independent experiments are indicated. As a reference compound, we used the antineoplastic agent doxorubicin, which reduced HepG2 cell viability by 55.0 ± 7.4% at a concentration of 1 μM.

### 3.5 Qualitative assessment of green credentials for the obtention of 1–3Gd_n_

Finally, the greenness of the methodology was evaluated in terms of CHEM21 (ref. 70) qualitative metrics at the first pass level. The results of this analysis are summarized in Table 3. Overall, the use of catalytic strategies (green flag) resulted in processes with moderate (>60%) to high atom efficiency (>85%) over the four steps. High atom efficiency is achieved using carbonates in the phenol alkylation step, which proceeds under neat conditions (green flag). Similar performance during the amination step is observed, where the synthesis of amino-alkyl derivatives is carried out in bio-derived solvent CPME with water as the only side-product, giving green flags in both cases. Overall, the avoidance of highly critical elements was achieved in most of the steps, except for the use of the Ru-based Shvo catalyst for the amination step. Regarding the energy requirement, three of the steps in this methodology perform positively, except for the alkylation step, where the inherent use of intensive reaction conditions is required. The four steps underperform regarding work-up procedures, given the usage of flash chromatography for intermediate purification, which will receive attention in future studies.

**Table 3:** Qualitative assessment of solvent use, inherent hazards of used chemicals, catalyst or reagent use, energy and workup methods for the synthesis of 3Gd1^a^

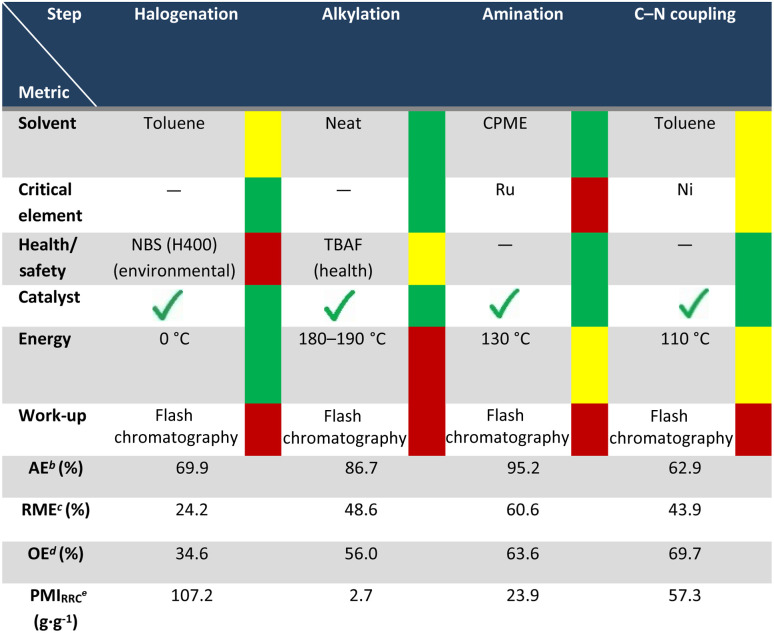

a Values calculated for the synthesis of 6-ethyl-8-methoxy-4-(4-methoxyphenyl)-3,4-dihydro-2*H*-benzo[*b*][1,4]oxazine (3Gd1) from 4-ethyl guaiacol (3G).

b AE (atom economy) = (molecular weight of product/total molecular weight of reactants) × 100.

c RME (reaction mass efficiency) = (mass of isolated products/total mass of reactants) × 100.

d OE (overall economy) = RME/AE.

e PMI (process mass intensity) = total mass process step (reagents + reactants + catalyst + solvent)/total mass product.

The calculation of process mass intensity values (PMI) is of great relevance in the pharmaceutical industry; therefore, such important metric was calculated for all the steps of the methodology in terms of the number of reagents, reactants and catalysts utilized (PMI_RRC_), affording exceptional values specifically in the case of the alkylation step where neat conditions were employed.

In addition, the synthetic procedure assessment based on the criteria of the 12 Principles of Green Chemistry^71^ is presented in Table 4, which comprises the use of renewable feedstocks, catalytic reagents and less hazardous chemicals, minimizing waste generation and leading to more atom-efficient processes.

**Table 4:** Application of the principles of green chemistry and analysis for the synthesis of 1–3Gd_n_

Principle	Step of the methodology/justification
Use of renewable feedstocks (7)	All the steps: Renewable lignin-derived guaiacols are valorized into biologically active 6-membered N-heterocycles
Use catalysts, not stoichiometric reagents (9)	All the steps: The use of catalysts in moderate to low concentrations (<5 mol%) is contemplated in every step
Maximize atom economy (2)	Step 2 (alkylation) and 3 (amination): Employing cyclic carbonates as phenol alkylating agents avoids the use of halo-alkyl derivatives. In the case of step 3, the hydrogen borrowing methodology enables efficient alcohol amination with H_2_O as a byproduct
Design less hazardous chemical syntheses (3)	Step 1 (halogenation): The reaction was performed under mild conditions by employing a less hazardous brominating agent, such as NBS.
Prevent waste (1)	Step 3 (amination): H_2_O as the only side product

## 4 Conclusions

The design of atom-efficient synthetic methodologies to produce nitrogen-containing chemicals is a powerful alternative for maximizing the viability of lignocellulosic biorefineries. In this contribution, we proposed a practical protocol for the upgrading of nonedible renewable resources, such as lignin-derived guaiacols, into a novel series of 1,2,3,4-tetrahydroquinolines and benzomorpholines. The combination of selective and catalytic methods together with the complete utilization of the intrinsic structure of these platform chemicals represents an advantageous strategy for the efficient synthesis of bio-based N-heterocycles with potential pharmaceutical applications. This approach is versatile and embraces multiple RCF-derived moieties, which are a growing family of compounds in emerging biorefinery concepts. We believe that the modularity of this approach will open the door to a more scrutinous molecular design and optimization, in which more active substituents can be incorporated to enhance the biological activity of these structures. This study provides proof of principle for obtaining relevant N-heterocyclic products from biomass. Future studies should be directed to the development of robust catalyst systems specifically suitable to accommodate more oxygenated starting materials, such as Earth-abundant metal catalysts to replace Ru-based ones for the amination of alcohols *via* hydrogen borrowing, or robust Ni or Fe catalysts to accomplish cross-coupling reactions.

## Author contributions

AC: conceptualization, investigation, original draft writing and editing; JH: investigation and writing; AKH: supervision and funding acquisition, KB: conceptualization, writing, review and editing, funding acquisition and supervision.

## Conflicts of interest

The authors declare no conflict of interest.

## Supplementary Material

SU-003-D5SU00151J-s001

## Data Availability

The data supporting this article have been included as part of the ESI.†
