# Supplementary material for: Lignin-derived guaiacols as platform chemicals for the modular synthesis of 1,2,3,4-tetrahydroquinolines and benzomorpholines
Source: RSC Sustain. 2025 Jul 2;3(9):4039–48. doi: 10.1039/d5su00151j (PMC12264585; doi:10.1039/d5su00151j)
Supplement: SU-003-D5SU00151J-s001 [file SU-003-D5SU00151J-s001.pdf]

Supplementary Information (SI) for RSC Sustainability.
This journal is © The Royal Society of Chemistry 2025

Supplementary information

Lignin-derived guaiacols as platform chemicals for the modular synthesis of 1,2,3,4-tetrahydroquinolines and benzomorpholines

Antonio A. Castillo-Garcia,[a,b] Jörg Haupenthal,[c,d] Anna K. H. Hirsch,[c,d,e] and Katalin Barta*,[a]

*Correspondence katalin.barta@uni-graz.at

1. General methods

Commercially available materials were purchased from Sigma-Aldrich or TCI Chemicals and were used as received without further purification.1-Hydroxytetraphenylcyclopentadienyl-(tetraphenyl-2,4-cyclopentadien-1-one)-μ hydrotetracarbonyldiruthenium(II) (Shvos catalyst) and bis(1,5-cyclooctadiene)nickel (0) were purchased from Strem chemicals. Cyclopentyl methyl ether (CPME, 99.9%, anhydrous) and toluene (99.9 %, anhydrous) were purchased from Sigma-Aldrich.

Column chromatography was performed using Merck silica gel type 9385 230-400 mesh and typically pentane and ethyl acetate as eluent.

Thin layer chromatography (TLC): Merck silica gel 60, 0.25 mm. The components were visualized by UV or $KMnO_4$ staining.

Analytical methods. Product identification was performed by GC-MS (Shimadzu QP2010 Ultra) with an HP-1MS column, and helium as carrier gas. GC-MS method: The temperature program started at 50 °C for 5 min, heated by 30 °C/minute to 250 °C and held for 15 min. Conversions and product selectivity were determined by GC-FID (Agilent Technologies 6890) with an HP-5MS column using nitrogen as carrier gas. GC-FID analysis method: The temperature program started at 50 °C for 5 min, heated by 30 °C/min to 320 °C and held for 15 min. ^1H and ^{13}C NMR spectra were recorded on a Varian Mercury Plus 400, Agilent MR 400 (400 and 101 MHz, respectively), Varian Inova 500 (500 and 126 MHz, respectively) and Bruker Avance NEO 600 (600 and 151 MHz, respectively) using $CDCl_3$ as a solvent. ^1H and ^{13}C NMR spectra were recorded at room temperature. Chemical shift values are reported in ppm with the solvent resonance as the internal standard ($CDCl_3$: 7.26 for 1H, 77.00 for ^{13}C). Data are reported as follows: chemical shifts, multiplicity (s = singlet, d = doublet, t = triplet, q = quartet, br. = broad, m = multiplet), coupling constants (Hz), and integration.

Cytotoxicity assay: Hep G2 (hepatocellular carcinoma) cells (2×105 cells per well) were seeded in 24-well, flat-bottomed plates. Culturing of cells, incubations and OD measurements were performed as described previously[1,2] with small modifications. Twenty-four hours after seeding the cells, the incubation was started by the addition of compounds in a final DMSO concentration of 1%. The living cell mass was determined after 48 hours in a PHERAstar microplate reader (BMG labtech, Ortenberg, Germany). At least two independent measurements were performed for each compound.

1.1 Synthesis of dihydroconiferyl alcohol (**1G**)

1G was synthesized by slight modification of the literature procedure.[3] A mixture of (E)-3-(4- hydroxy-3-methoxyphenyl)acrylic acid (10 g, 51.54 mmol) and 5% Pd/C (0.2 g) in methanol/EtOAc (50 mL, 1:1) were placed in a high-pressure Parr autoclave. The reactor was sealed, purged 3 times with H_2 and then pressurized with H_2 (40 bar) and stirred at room temperature for 18 h. After filtering through a Celite plug, the solvent was evaporated under reduced pressure to provide 10 g of 3-(4-hydroxy-3-methoxyphenyl)propanoic acid (51.04 mmol, 99% yield), which was used without further purification. To a rapidly stirred suspension of $LiAlH_4$ (1.45 g, 38.26 mmol) in 50 mL of THF was added over 50 min a solution of 3-(4-hydroxy-3-methoxyphenyl)propanoic acid (5 g, 25.51 mmol) in 20 mL of THF at 0 °C. After the addition was complete, the reaction mixture was allowed to room temperature and refluxed for 60 min, and then the mixture was cooled again to room temperature, poured into ice water which was then poured into a solution of 5% HCl over ice, and extracted once with diethyl ether (100 mL) and twice with ethyl acetate (50 mL). The combined organic extract was washed with a saturated solution of $NaHCO_3$ (50 mL) and brine (100 mL), dried over anhydrous $MgSO_4$ and the solvent was evaporated under reduced pressure to provide 3.7 g of dihydroconiferyl alcohol (80% yield).

1.2 Synthesis of 3-(3,4-dimethoxyphenyl)propan-1-ol (**1Ga**)

The synthesis of 1Ga was carried out by following our previous report.[4] **Step 1**: A 20 mL oven-dried microwave vial equipped with stirring bar was charged with **1G** (364 mg, 2 mmol), K_2CO_3 (2.7 mg, 0.02 mmol) and dimethylcarbonate (1.8 g, 20 mmol). Afterwards, the vial was capped and placed into a heating block at 160 °C and stirred during 16 h. Then, the mixture was diluted in EtOAc (2 mL) and the solution was filtered and concentrated under reduced pressure. **Step 2**: The product obtained from Step 1 was dissolved in MeOH/H_2O (5:1, 2.5 mL) and NaOH (54 mg, 1.3 mmol) was added. The mixture was stirred at 40 °C during 4 h. After reaction completion, the mixture was filtered and concentrated under vacuum. The residue was then dissolved in Et_2O (10 mL) and washed with brine (2x10 mL). Finally, the organic phase is dried over anhydrous $MgSO_4$ and the solvent was evaporated under reduced pressure affording 309 mg of **1Ga** (79% yield).

2. General experimental procedures and optimization of reaction conditions

2.1 General procedure for the synthesis of **1Gb** and **2-3Ga**

An oven-dried 10 mL glass vial was charged with **1Ga** or **2-3G** (0.5 mmol), NBS (265 mg, 1.5 mmol), **C1** (3.5 mg, 0.025 mmol) and anhydrous toluene (5 mL). Then, the mixture was stirred at 0 °C for 0.5 h. After reaction completion, the mixture was quenched with $NaHCO_3$ (5 mL) and extracted with EtOAc (3x5 mL). The organic phase was washed with water (2x10 mL) and saturated brine (2x10 mL). Finally, the combined organic phase was dried over Na_2SO_4 and a small aliquot (0.3 mL) was analyzed by GC-MS/GC-FID to monitor the product formation.

Table S1. Optimization of reaction conditions: Selective ortho-bromination of 4-ethylguaiacol (**3G**)[a]

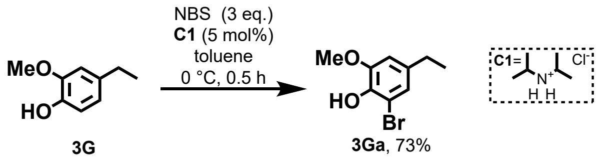

Entry	Deviation of standard conditions	Conversion (%)[a]	3Ga (%)[b]
1	-	98	83 (73)[c]
2	0.5 mmol of NBS	27	25
3	0.1 mmol of NBS	47	44
4	t= 1 h	98	84
5	Without **C1**	19	10
6	room temperature	45	13
7	MeOH instead of toluene	73	40
8	**2G** instead of **3G**	88	82[b] (**2Ga**)

[a] General conditions: **3G** (76 mg, 0.5 mmol), NBS (265 mg, 1.5 mmol), **C1** (3.5 mg, 0.025 mmol), toluene (5 mL), 0 °C, 0.5 h. [b] Conversion and yields were determined by GC-FID analysis based on the calculation of response factors via calibration using 3,5-dimethyl phenol as internal standard. [c] Isolated yield.

Supplementary Note 1. Analytical data of selective ortho-bromination of 4-ethylguaiacol

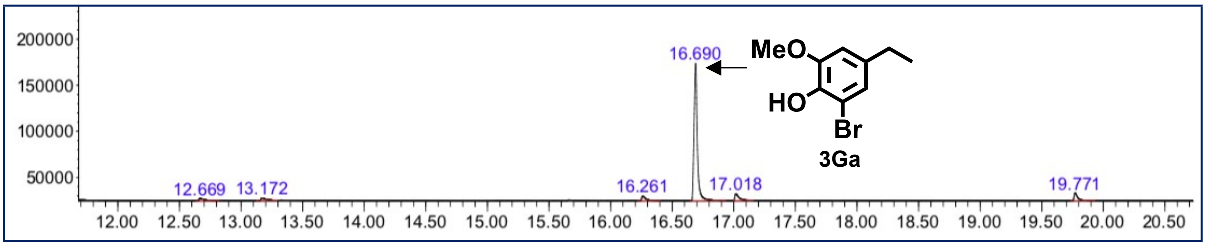

Fig. S1 GC-FID chromatogram of the mixture of products obtained under standard conditions.

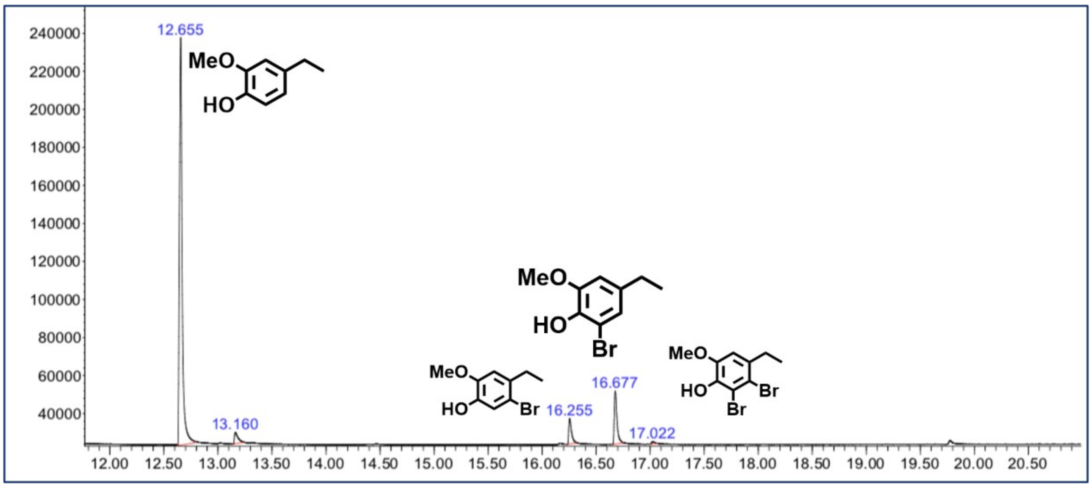

Fig. S2 GC-FID chromatogram of the mixture of products obtained in the absence of **C1**

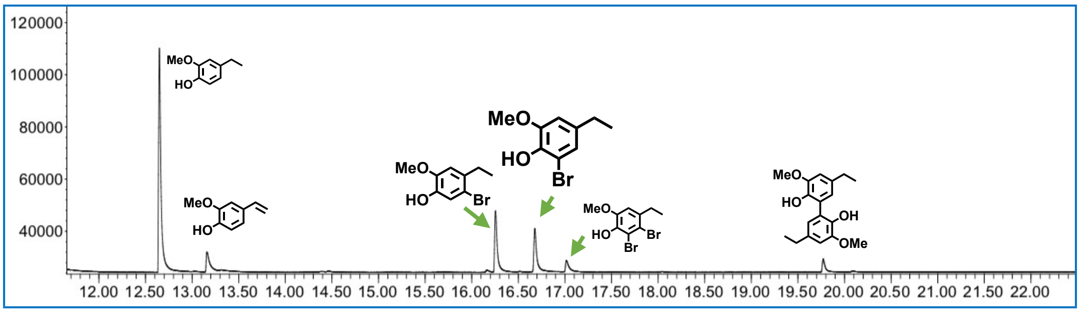

Fig. S3 GC-FID chromatogram of the mixture of products obtained at room temperature

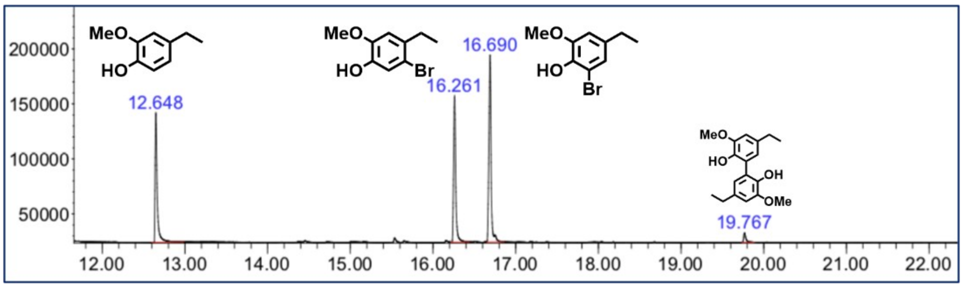

Fig. S4 GC-FID chromatogram of the mixture of products obtained with MeOH as solvent.

2.2 General procedure for the hydroxylalkylation of **2Ga** and **3Ga** with ethylene carbonate (**EC**)

An oven-dried microwave 20 mL vial equipped with stirring bar was charged with **2-3Ga** (1.0 mmol), ethylene carbonate (105.6 mg, 1.2 mmol), tetrabutylammonium fluoride (13.0 mg, 0.05 mmol) and DMF (0.2 mL). Then, the vial was capped and placed into a heating block at 190 °C and stirred during 1 h. After reaction completion, the mixture was diluted in MeOH (2 mL) and filtered with a PTFE septum. Then, the solvent was removed under reduced pressure and the product was isolated by flash chromatography using a mixture of ethyl acetate/pentane as eluent.

2.3 General procedure for Ru-catalyzed N-alkylation of anilines with **1-3Gb**

An oven-dried Schlenk tube equipped with stirring bar was charged with **1-3Gb** (0.26 mmol), amine (0.2 mmol), **Shvo catalyst** (8.5 mg, 0.008 mmol) and cyclopentyl methylether (CPME, 1.5 mL). Then, the Schlenk tube was subsequently connected to an argon line and vacuum-argon exchange was performed three times. The Schlenk tube was capped and the mixture was rapidly stirred at room temperature for 1 min, then was placed into a pre-

heated oil bath at 130 °C and stirred for 16 h. After completion, the reaction mixture was cooled down to room temperature and a small aliquot (0.3 mL) was analyzed by GC-FID and GC-MS to determine conversion and yield.

Supplementary Note 2. Ru-catalyzed N-alkylation of **3G-OH** with p-anisidine

The catalytic amination of the non-brominated derivative **3G-OH** with p-anisidine was carried out under the standard conditions. However, this compound displayed a significant lower conversion (23%) in comparison to the brominated derivative **3Gb**, where a 96% conversion was observed

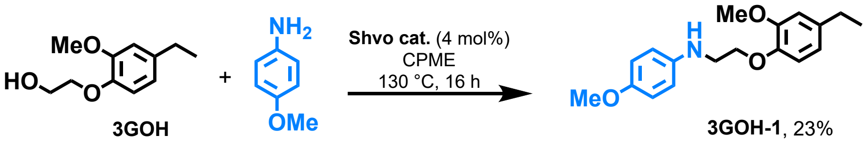

2.4 General procedure for Ni-catalyzed intramolecular C-N coupling:

Synthesis of 1,2,3,4-tetrahydroquinolines and benzomorpholines (**1-3Gd$_n$**)

An oven-dried Schlenk tube equipped with stirring bar was charged with N-alkylated derivative **1-3Gc$_n$** (0.1 mmol), NaOtBu (15 mg, 0.15 mmol), bis(1,5-cyclooctadiene)nickel(0) (1.4 mg, 0.005 mmol), IPr·HCl (2.2 mg, 0.005 mmol) and toluene (1.5 mL). Then, the Schlenk tube was subsequently connected to an argon line, toluene was added under argon stream and vacuum-argon exchange was performed three times. The Schlenk tube was capped and the mixture was rapidly stirred at room temperature for 1 min, then was placed into a pre-heated oil bath at 110 °C and stirred for 16 h. Finally, the reaction mixture was cooled down to room temperature. Conversion and yield were measured by GC-FID, identification of products was carried out by GC-MS.

Table S2. Intramolecular C–N coupling of **3Gc1**: Establishing optimal reaction conditions[a]

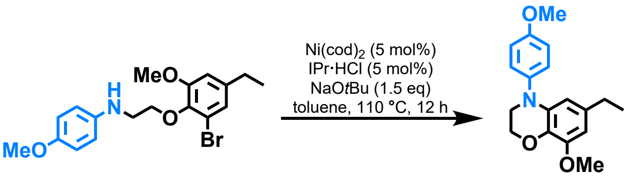

Entry	Deviation from above	3Gd1 (%)[b]
1	**none**	89 (77)[c]
2	Other NHC-Carbenes (IMes, SIPr, SIMes)	>10
3	Bisphosphine ligands (dppf, dcpp, dppp, dcpe, dppe, Xanthphos, N-Xantphos)	40-70
4	Ni(II) precatalysts $Ni(dme)Br_2$, $Ni(OTf)_2$, $Ni(PCy_3)_2Cl_2$	>10
5	T= 80 °C	8
6	Cs_2CO_3 instead of NaOtBu	39

[a] General reaction conditions: **3Gc1** (0.1 mmol), Ni catalyst (0.005 mmol), ligand (0.005 mmol), NaOtBu (1.5 eq), toluene, 110 °C, 16 h. [b] Yield determination by GC-FID analysis was carried out based on the calculation of response factors via calibration using 3,5-dimethyl phenol as internal standard. [c] Isolated yield.

Supplementary Note 3. Calculation of atom efficiency (AE) of products **1Gd2**, **2Gd2** and **3Gd2**

**Atom efficiency (AE)= (molecular weight of product/ total molecular weight of reactants) x 100**	
**Compound: 1Gd2**<br>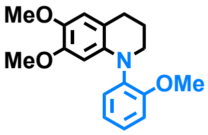	
**Step**	**AE (%)**
Halogenation	73.4
Alkylation	72.6
Amination	95.4
C-N coupling	78.8

**Compound: 2Gd2**<br>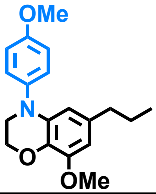	
**Step**	**AE (%)**
Halogenation	71.1
Alkylation	86.7
Amination	95.6
C-N coupling	79.6

**Compound: 3Gd2**<br>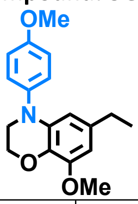	
**Step**	**AE (%)**
Halogenation	69.9
Alkylation	86.4
Amination	95.2
C-N coupling	78.8

3. Characterization data of isolated compounds

3-(3,4-dimethoxyphenyl)propan-1-ol (**1Ga**)

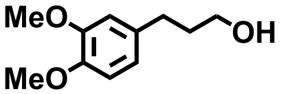

The compound was synthesized according to the General method (**1.2)**. A dark-yellow oil (850 mg, 79%) was obtained after flash chromatography (SiO_2, Pentane/EtOAc 80:20). Characterization data is in accordance with the previously reported in the literature.[4] **^{1}H NMR** (300 MHz, CDCl3) δ 6.82 – 6.67 (m, 1H), 3.65 (t, J = 6.7 Hz, 1H), 2.64 (dd, J = 8.7, 6.7 Hz, 1H), 2.00 – 1.68 (m, 1H). **^{13}C NMR** (75 MHz, $CDCl_3$) δ 148.9, 147.2, 134.5, 120.6, 111.8, 62.3, 56.0, 55.9, 34.4, 31.8. **HRMS** (ESI$^+$ m/z). Calculated for $[M+H]^+$: 196.115213, found: 196.238196.

3-(2-bromo-4,5-dimethoxyphenyl)propan-1-ol (**1Gb**)

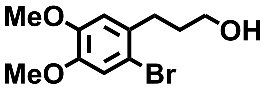

The compound was synthesized according to the General procedure (**2.1**). A light-yellow solid (103 mg, 75%) was obtained after flash chromatography (SiO_2, Pentane/EtOAc 70:30) **^{1}H NMR** (300 MHz, $CDCl_3$) δ 6.92 (s, 1H), 6.64 (s, 1H), 4.03 (t, J = 6.5 Hz, 2H), 3.78 (s, 3H), 3.77 (s, 3H), 2.71 – 2.60 (m, 2H), 1.94 – 1.78 (m, 1H). **^{13}C NMR** (75 MHz, $CDCl_3$) δ 148.4, 148.0, 132.2, 115.6, 114.0, 112.9, 63.7, 56.1, 56.0, 32.2, 29.0. **HRMS** (ESI$^+$ m/z). Calculated for $[M+H]^+$: 274.026682, found: 275.261216.

N-(3-(2-bromo-4,5-dimethoxyphenyl)propyl)-4-methoxyaniline (**1Gc1**)

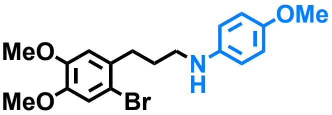

The compound was synthesized according to the General procedure (**2.3**). 4-methoxyaniline (25 mg, 0.2 mmol) affords **1Gc1** (66 mg, 87%). A dark-purple oil was obtained after column chromatography (SiO_2, Pentane/EtOAc 70:30). **^{1}H NMR** (300 MHz, $CDCl_3$) δ 7.00 (s, 1H), 6.84 – 6.70 (m, 3H), 6.63 – 6.52 (m, 2H), 3.85 (s, 3H), 3.81 (s, 3H), 3.75 (s, 3H), 3.12 (t, J = 6.9 Hz, 2H), 2.84 – 2.73 (m, 2H), 1.91 (d, J = 1.7 Hz, 2H). **^{13}C NMR** (75 MHz, $CDCl_3$) δ 152.2, 148.5, 148.1, 142.8, 133.1, 115.8, 115.1, 114.2, 113.1, 56.3, 56.2, 56.0, 44.4, 33.4, 30.1. **HRMS** (ESI$^+$ m/z). Calculated for $[M+H]^+$: 379.082936, found: 379.896621.

N-(3-(2-bromo-4,5-dimethoxyphenyl)propyl)-3,4-dimethoxyaniline (**1Gc2**)

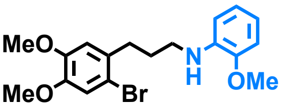

The compound was synthesized according to the General procedure (**2.3**). 2-methoxyaniline (25 mg, 0.2 mmol) affords **1Gc2** (59 mg, 80%). A dark-purple was obtained after column chromatography (SiO_2, Pentane/EtOAc 70:30). **^{1}H NMR** (300 MHz, $CDCl_3$) δ 7.01 (s, 1H), 6.87 (td, J = 7.9, 1.6 Hz, 1H), 6.77 (dd, J = 7.9, 1.6 Hz, 1H), 6.72 (s, 1H), 6.64 (ddd, J = 15.9, 7.9, 1.6 Hz, 2H), 6.58 (d, J = 1.6 Hz, 1H), 3.85 (d, J = 2.4 Hz, 6H), 3.80 (s, 3H), 3.18 (t, J = 6.9 Hz, 2H), 2.86 – 2.75 (m, 2H), 2.05 – 1.89 (m, 2H). **^{13}C NMR** (75 MHz, $CDCl_3$) δ 148.5, 148.0, 146.9, 138.4, 133.1, 121.4, 116.4, 115.7, 114.1, 113.1, 109.5, 56.3, 55.5, 43.1, 33.4, 29.9. **HRMS** (ESI$^+$ m/z). Calculated for $[M+H]^+$: 379.062236, found: 380.212269.

1-(4-((3-(2-bromo-4,5-dimethoxyphenyl)propyl)amino)phenyl)ethan-1-one (**1Gc3**)

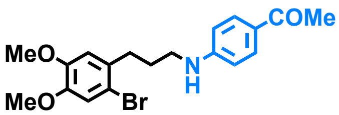

The compound was synthesized according to the General procedure (**2.3**). 4-aminoacetophenone (27 mg, 0.2 mmol) affords **1Gc3** (71 mg, 90%). A light-yellow solid was obtained after column chromatography (SiO_2, Pentane/EtOAc 70:30). **^{1}H NMR** (300 MHz, $CDCl_3$) δ 7.82 (d, J = 8.8 Hz, 1H), 7.00 (s, 1H), 6.69 (s, 1H), 6.54 (d, J = 8.8 Hz, 1H), 3.85 (s, 2H), 3.80 (s, 2H), 3.24 (t, J = 6.9 Hz, 1H), 2.78 (dd, J = 8.6, 6.6 Hz, 1H), 2.49 (s, 2H), 2.02 – 1.84 (m, 1H). **^{13}C NMR** (75 MHz, $CDCl_3$) δ 196.5, 152.3, 148.6, 148.2, 132.6, 131.0, 126.8, 115.7, 114.1, 113.0, 111.5, 56.3, 56.2, 42.7, 29.8, 26.1. **HRMS** (ESI^+ m/z). Calculated for $[M+H]^+$: 391.082588, found: 391.022634.

4-((3-(2-bromo-4,5-dimethoxyphenyl)propyl)amino)benzonitrile (**1Gc4**)

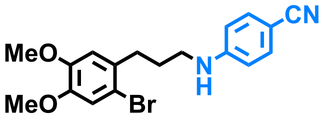

The compound was synthesized according to the General procedure (**2.3**). 4-benzonitrile (24 mg, 0.2 mmol) affords **1Gc4** (51 mg, 68%). A light-yellow oil was obtained after column chromatography (SiO_2, Pentane/EtOAc 70:30). **^{1}H NMR** (300 MHz, $CDCl_3$) δ 7.40 (d, J = 8.8 Hz, 2H), 7.00 (s, 1H), 6.68 (s, 1H), 6.53 (d, J = 8.8 Hz, 2H), 3.85 (s, 3H), 3.80 (s, 3H), 3.19 (t, J = 7.1 Hz, 2H), 2.81 – 2.74 (m, 2H), 1.93 (dt, J = 15.5, 7.1 Hz, 2H). **^{13}C NMR** (75 MHz, $CDCl_3$) δ 151.4, 148.6, 148.2, 133.9, 132.4, 120.6, 115.7, 114.1, 113.0, 112.2, 98.7, 56.2, 42.5, 33.2, 29.6. **HRMS** (ESI^+ m/z). Calculated for $[M+H]^+$: 374.062930, found: 374.264817.

N-(3-(2-bromo-4,5-dimethoxyphenyl)propyl)-4-(methylthio)aniline (**1Gc5**)

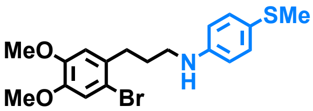

The compound was synthesized according to the General procedure (**2.3**). 4-(methylthio)aniline (28 mg, 0.2 mmol) affords **1Gc5** (54 mg, 69%). A dark-yellow oil was obtained after column chromatography (SiO_2, Pentane/EtOAc 70:30). **^{1}H NMR** (300 MHz, $CDCl_3$) δ 7.21 (d, J = 8.7 Hz, 2H), 7.00 (s, 1H), 6.70 (s, 1H), 6.54 (d, J = 8.7 Hz, 2H), 3.85 (s, 7H), 3.15 (t, J = 6.9 Hz, 2H), 2.83 – 2.61 (m, 2H), 2.40 (s, 3H), 2.00 – 1.84 (m, 2H). **^{13}C NMR** (75 MHz, $CDCl_3$) δ 148.5, 148.0, 147.3, 132.9, 131.7, 124.1, 114.1, 113.4, 113.0, 56.3, 56.2, 43.3, 33.3, 29.9, 19.4. **HRMS** (ESI^+ m/z). Calculated for $[M+H]^+$: 397.053624 , found: 397.642216 .

N-(3-(2-bromo-4,5-dimethoxyphenyl)propyl)-4-fluoroaniline (**1Gc6**)

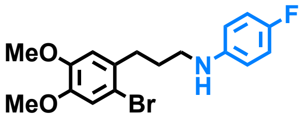

The compound was synthesized according to the General procedure (**2.3**). 4-fluoroaniline (23mg, 0.2 mmol) affords **1Gc6** (67 mg, 91%). A yellow oil was obtained after column chromatography (SiO_2, Pentane/EtOAc 70:30). **^{1}H NMR** (300 MHz, $CDCl_3$) δ 7.40 (d, J = 8.8 Hz, 2H), 7.00 (s, 1H), 6.68 (s, 1H), 6.53 (d, J = 8.8 Hz, 2H), 3.85 (s, 3H), 3.80 (s, 3H), 3.23 – 3.16 (m, 2H), 2.82 – 2.73 (m, 2H), 1.93 (m, 2H). **^{13}C NMR** (75 MHz, $CDCl_3$) δ 151.4, 148.6, 148.2, 133.8, 132.4, 120.6, 115.7, 114.1, 113.0 (d, J_{C-F} = 7.4 Hz), 112.3, 98.7, 56.3, 42.5, 33.2, 29.6. **HRMS** (ESI^+ m/z). Calculated for $[M+H]^+$: 367.062646, found: 367.243910.

N-(3-(2-bromo-4,5-dimethoxyphenyl)propyl)-3-fluoroaniline (**1Gc7**)

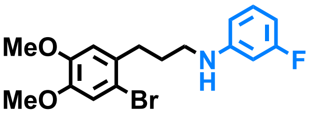

The compound was synthesized according to the General procedure (**2.3**). 3-fluoroaniline (23 mg, 0.2 mmol) affords **1Gc7** (69 mg, 94%). A yellow oil was obtained after column chromatography (SiO_2, Pentane/EtOAc 70:30). **^{1}H NMR** (300 MHz, $CDCl_3$) δ 7.15 – 6.98 (m, 2H), 6.70 (d, J = 1.8 Hz, 1H), 6.43 – 6.22 (m, 2H), 3.85 (s, 3H), 3.81 (s, 3H), 3.14 (m, 2H), 2.00 – 1.84 (m, 2H). **^{13}C NMR** (75 MHz, $CDCl_3$) δ 150.3, 148.1, 132.8, 130.4, 130.3, 115.7, 114.1, 113.0, 108.8, 103.7 (d, J_{C-F} = 21.7 Hz), 99.4 (d, J_{C-F} = 25.4 Hz), 56.30, 43.2, 33.3, 29.8. **HRMS** (ESI^+ m/z). Calculated for $[M+H]^+$: 367.062646, found: 367.661824.

N-(3-(2-bromo-4,5-dimethoxyphenyl)propyl)-4-(trifluoromethyl)aniline (**1Gc8**)

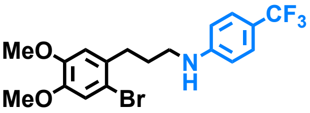

The compound was synthesized according to the General procedure (**2.3**). 4-trifluoromethyl-aniline (33 mg, 0.2 mmol) affords **1Gc8** (70 mg, 83%). A purple oil was obtained after column chromatography (SiO_2, Pentane/EtOAc 70:30). **^{1}H NMR** (300 MHz, $CDCl_3$) δ 7.44 – 7.34 (m, 2H), 7.01 (s, 1H), 6.69 (s, 1H), 6.58 (d, J = 8.5 Hz, 2H), 3.86 (s, 3H), 3.80 (s, 3H), 3.20 (t, J = 6.9 Hz, 2H), 2.78 (dd, J = 8.5, 6.9 Hz, 2H), 1.94 (dq, J = 8.5, 6.9 Hz, 2H). **^{13}C NMR** (75 MHz, $CDCl_3$) δ 150.8, 148.6, 148.1, 132.6, 126.7 (d, J_{C-F}= 3.8 Hz), 115.7, 114.1, 113.0, 111.8, 56.3, 42.8, 33.3, 29.7. **HRMS** (ESI^+ m/z). Calculated for $[M+H]^+$: 417.063529, found: 417.364821.

6,7-dimethoxy-1-(2-methoxyphenyl)-1,2,3,4-tetrahydroquinoline (**1Gd2**)

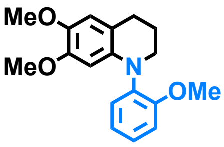

The compound was synthesized according to the General procedure (**2.4**). **1Gc2** (38 mg, 0.1 mmol) affords **1Gd2** (23 mg, 76%). A dark-red oil was obtained after column chromatography (SiO_2, Pentane/EtOAc 70:30). **^{1}H NMR** (300 MHz, $CDCl_3$) δ 7.01 (s, 1H), 6.87 (td, J = 7.9, 1.6 Hz, 1H), 6.77 (dd, J = 7.9, 1.6 Hz, 2H), 6.72 (s, 1H), 6.68 (dd, J = 7.9, 1.6 Hz, 1H), 6.64 – 6.53 (m, 2H), 3.85 (d, J = 2.1 Hz, 8H), 3.81 (d, J = 2.6 Hz, 3H), 3.18 (q, J = 6.7 Hz, 2H), 2.80 (dd, J = 8.5, 6.7 Hz, 2H), 2.02 – 1.93 (m, 2H). **^{13}C NMR** (75 MHz, $CDCl_3$) δ 148.5, 148.0, 147.0, 138.3, 133.1, 121.4, 116.5, 115.7, 114.2, 113.2, 110.0, 56.3, 55.6, 43.1, 33.4, 29.9. **HRMS** (ESI^+ m/z). Calculated for $[M+H]^+$: 299.172810, found: 299.372616.

1-(4-(6,7-dimethoxy-3,4-dihydroquinolin-1(2H)-yl)phenyl)ethan-1-one (**1Gd3**)

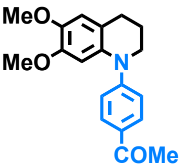

The compound was synthesized according to the General procedure (**2.4**). **1Gc3** (39 mg, 0.1 mmol) affords **1Gd3** (21 mg, 68%). A dark-yellow oil was obtained after column chromatography (SiO2, Pentane/EtOAc 70:30). **^{1}H NMR** (300 MHz, $CDCl_3$) δ 7.91 – 7.80 (m, 1H), 7.16 – 7.09 (m, 1H), 6.70 (s, 1H), 6.64 (s, 1H), 3.86 (s, 3H), 3.71 (s, 1H), 3.68 (t, J = 6.5 Hz, 1H), 2.71 (t, J = 6.5 Hz, 1H), 2.54 (s, 1H), 2.01 – 1.88 (m, 1H). **^{13}C NMR** (75 MHz, $CDCl_3$) δ 196.6, 152.9, 147.4, 144.7, 134.5, 130.1, 129.1, 128.3, 121.8, 118.2, 112.2, 105.2, 56.3, 49.3, 26.9, 23.7. **HRMS** (ESI^+ m/z). Calculated for $[M+H]^+$: 311.386641, found: 311.994612.

4-(6,7-dimethoxy-3,4-dihydroquinolin-1(2H)-yl)benzonitrile (**1Gd4**)

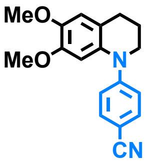

The compound was synthesized according to the General procedure (**2.4**). **1Gc4** (38 mg, 0.1 mmol) affords **1Gd4** (18 mg, 61%). A yellow solid was obtained after column chromatography (SiO2, Pentane/EtOAc 70:30).**^{1}H NMR** (300 MHz, $CDCl_3$) δ 7.28 – 7.26 (m, 1H), 7.24 (d, J = 2.2 Hz, 1H), 7.13 – 7.09 (m, 2H), 6.62 (s, 1H), 6.45 (s, 1H), 3.85 (s, 3H), 3.69 (s, 3H), 3.61 – 3.56 (m, 2H), 2.77 (t, J = 6.5 Hz, 2H), 2.00 – 1.90 (m, 3H). **^{13}C NMR** (75 MHz, $CDCl_3$) δ 148.2, 147.6, 143.3, 136.8, 129.2, 126.9, 123.8, 118.5, 112.8, 103.2, 56.4, 56.0, 51.0, 29.8, 27.1. **HRMS** (ESI$^+$ m/z). Calculated for $[M+H]^+$: 294.351418, found: 295.798511.

6,7-dimethoxy-1-(4-(methylthio)phenyl)-1,2,3,4-tetrahydroquinoline (**1Gd5**)

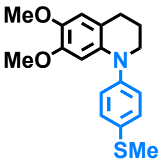

The compound was synthesized according to the General procedure (**2.4**). **1Gc5** (40 mg, 0.1 mmol) affords **1Gd5** (19 mg, 59%). A yellow oil was obtained after column chromatography (SiO_2, Pentane/EtOAc 70:30). **^{1}H NMR** (300 MHz, $CDCl_3$) δ 7.36 – 7.15 (m, 2H), 7.20 – 7.07 (m, 2H), 6.61 (s, 1H), 6.46 (s, 1H), 3.85 (s, 3H), 3.68 (s, 3H), 3.65 – 3.54 (m, 2H), 2.77 (m, 2H), 2.49 (s, 3H), 2.00 – 1.88 (m, 2H). **^{13}C NMR** (75 MHz, $CDCl_3$) δ 147.5, 147.4, 143.0, 137.1, 130.7, 128.8, 123.4, 118.0, 112.8, 103.0, 56.4, 56.0, 51.0, 27.1, 22.5, 17.1. **HRMS** (ESI$^+$ m/z). Calculated for $[M+H]^+$: 315.431320, found: 315.774021.

1-(4-fluorophenyl)-6,7-dimethoxy-1,2,3,4-tetrahydroquinoline (**1Gd6**)

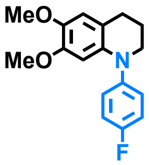

The compound was synthesized according to the General procedure (**2.4**). **1Gc6** (37 mg, 0.1 mmol) affords **1Gd6** (25 mg, 89%). A dark-red oil was obtained after column chromatography (SiO_2, Pentane/EtOAc 70:30). **^{1}H NMR** (300 MHz, $CDCl_3$) δ 7.33 – 7.18 (m, 2H), 7.21 – 7.05 (m, 2H), 6.62 (s, 1H), 6.45 (s, 1H), 3.85 (s, 3H), 3.69 (s, 3H), 3.63 – 3.54 (m, 1H), 2.77 (t, J = 6.5 Hz, 2H), 2.02 – 1.86 (m, 2H). **^{13}C NMR** (75 MHz, $CDCl_3$) δ 148.2, 147.6, 143.3, 136.8, 129.2, 126.9, 123.8, 112.8, 103.2, 56.4, 56.0, 51.0, 29.8, 27.1, 22.5. **HRMS** (ESI$^+$ m/z). Calculated for $[M+H]^+$: 287.331476, found: 287.291690.

1-(3-fluorophenyl)-6,7-dimethoxy-1,2,3,4-tetrahydroquinoline (**1Gd7**)

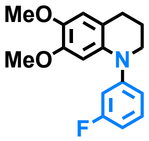

The compound was synthesized according to the General procedure (**2.4**). **1Gc7** (37 mg, 0.1 mmol) affords **1Gd7** (23 mg, 86%). A dark-purple oil was obtained after column chromatography (SiO_2, Pentane/EtOAc 70:30**)**. **^{1}H NMR** (300 MHz, $CDCl_3$) δ 7.20 (td, J = 8.2, 7.3, 6.5 Hz, 1H), 6.92 (ddd, J = 8.2, 2.5, 0.9 Hz, 1H), 6.84 (dt, J = 8.2, 2.5 Hz, 1H), 6.64 (ddd, J = 8.2, 2.5, 0.9 Hz, 1H), 6.61 (s, 1H), 6.56 (s, 1H), 3.85 (s, 3H), 3.70 (s, 3H), 3.65 – 3.55 (m, 2H), 2.73 (t, J = 6.5 Hz, 2H), 1.93 (p, J = 6.5 Hz, 3H). **^{13}C NMR** (75 MHz, $CDCl_3$) δ 147.5, 143.8, 136.1, 130.1, 123.9, 119.5, 117.0, 112.6, 108.6 (d, J_{C-F} = 21.5 Hz), 107.9, 104.1, 56.4, 56.1, 50.6, 27.0, 22.8. **HRMS** (ESI$^+$ m/z). Calculated for $[M+H]^+$: 287.331476, found: 288.100356.

2-bromo-6-methoxy-4-propylphenol (**2Ga**)

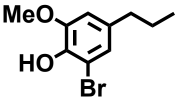

The compound was synthesized according to the General procedure (**2.1**). A yellow oil (99 mg, 82%) was obtained after column chromatography (SiO_2, Pentane/EtOAc 95:5). **^{1}H NMR** (300 MHz, $CDCl_3$) δ 6.91 (d, J = 1.8 Hz, 1H), 6.62 (d, J = 1.8 Hz, 1H), 5.76 (s, 1H), 3.88 (s, 3H), 2.52 – 2.45 (m, 2H), 1.60 (dq, J = 14.7, 7.3 Hz, 2H), 0.93 (t, J = 7.3 Hz, 3H). **^{13}C NMR** (75 MHz, $CDCl_3$) δ 147.0, 141.0, 135.5, 124.2, 110.4, 107.9, 56.3, 37.5, 24.7, 13.8. **HRMS** (ESI^+ m/z). Calculated for $[M+H]^+$: 244.011930, found: 245.192088.

2-bromo-4-ethyl-6-methoxyphenol (**3Ga**)

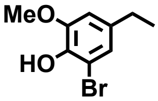

The compound was synthesized according to the General procedure (**2.1**). An off-white solid (83 mg, 73%) was obtained after column chromatography (SiO_2, Pentane/EtOAc 95:5). **^{1}H NMR** (300 MHz, $CDCl_3$) δ 6.93 (d, J = 1.8 Hz, 1H), 6.64 (d, J = 1.8 Hz, 1H), 5.75 (s, 1H), 3.89 (s, 3H), 2.56 (q, J = 7.6 Hz, 2H), 1.21 (t, J = 7.6 Hz, 3H). **^{13}C NMR** (75 MHz, $CDCl_3$) δ 147.0, 140.9, 137.0, 123.5, 109.9, 107.9, 56.2, 28.3, 15.7. **HRMS** (ESI^+ m/z). Calculated for $[M+H]^+$: 229.991618, found: 229.998458.

2-(2-bromo-6-methoxy-4-propylphenoxy)ethan-1-ol (**2Gb**)

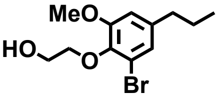

The compound was synthesized according to the General procedure (**2.2**). A yellow oil (231 mg, 80 %) was obtained after column chromatography (SiO_2, Pentane/EtOAc 70:30). **^{1}H NMR** (300 MHz, $CDCl_3$) δ 6.95 (d, J = 1.9 Hz, 1H), 6.66 (d, J = 1.9 Hz, 1H), 4.17 – 4.10 (m, 2H), 3.83 (s, 3H), 3.81 (m, 5H), 2.53 – 2.45 (m, 2H), 1.67 – 1.52 (m, 2H), 0.92 (t, J = 7.3 Hz, 3H). **^{13}C NMR** (75 MHz, $CDCl_3$) δ 153.0, 140.2, 124.5, 117.3, 111.9, 75.3, 61.8, 56.0, 37.5, 24.4, 13.7. **HRMS** (ESI^+ m/z). Calculated for $[M+H]^+$: 288.171340, found: 289.042988.

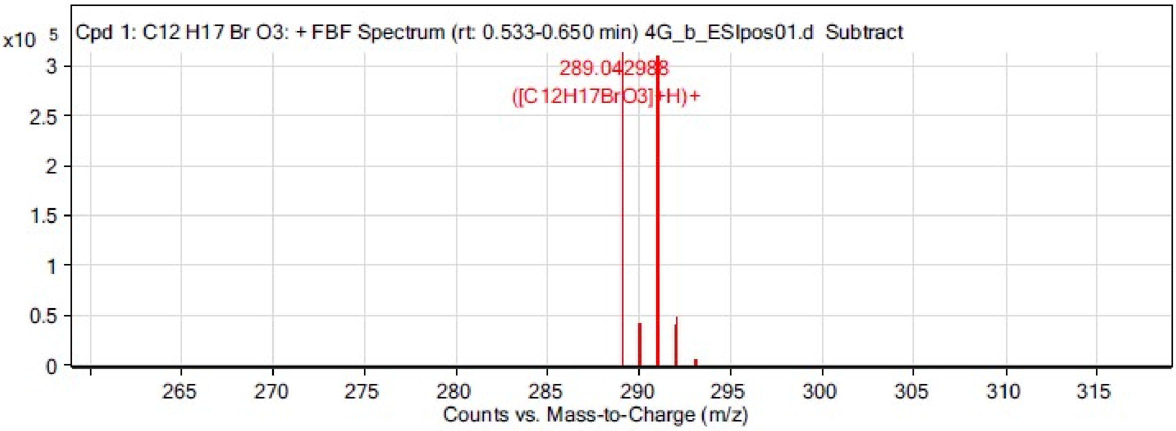

2-(2-bromo-4-ethyl-6-methoxyphenoxy)ethan-1-ol (**3Gb**)

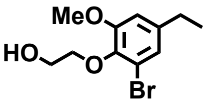

The compound was synthesized according to the General procedure (**2.2**). A white solid (195 mg, 71%) was obtained after column chromatography (SiO_2, Pentane/EtOAc 70:30).**^{1}H NMR** (400 MHz, $CDCl_3$) δ 6.98 (s, 1H), 6.69 (s, 1H), 4.18 – 4.13 (m, 2H), 3.86 (s, 3H), 3.84 – 3.80 (m, 2H), 2.82 (d, J = 68.5 Hz, 1H), 2.58 (q, J = 7.6 Hz, 2H), 1.22 (t, J = 7.6 Hz, 3H). **^{13}C NMR** (101 MHz, $CDCl_3$) δ 153.2, 143.1, 141.8, 124.1, 117.5, 111.5, 75.5, 61.9, 56.2, 28.6, 15.5. **HRMS** (ESI^+ m/z). Calculated for $[M+H]^+$: 274.911302, found: 275.027699.

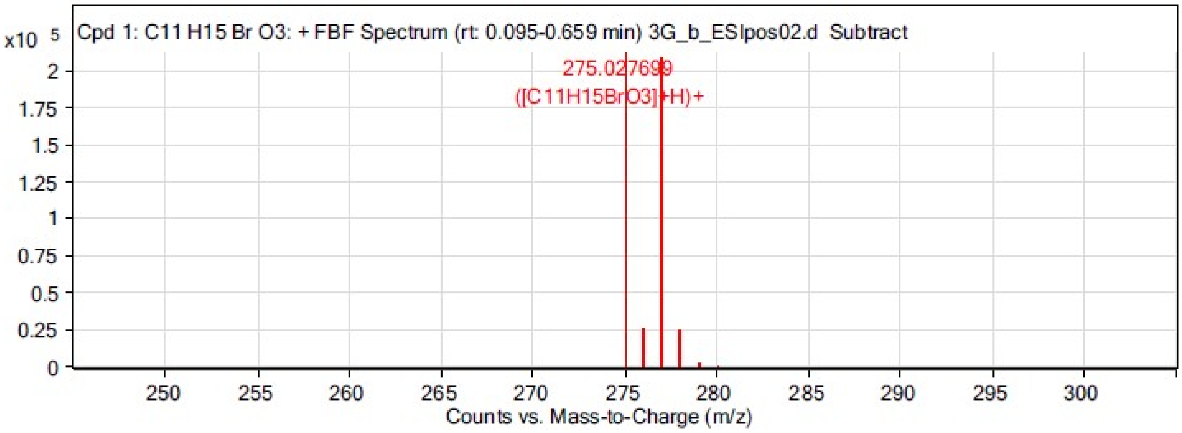

N-(2-(2-bromo-6-methoxy-4-propylphenoxy)ethyl)-4-methoxyaniline (**2Gc1**)

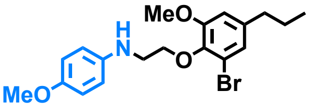

The compound was synthesized according to the General procedure (**2.3**). 4-Methoxyaniline (25 mg, 0.2 mmol) affords **2Gc1** (56 mg, 71%). A purple oil was obtained after column chromatography (SiO_2, Pentane/EtOAc 70:30). **^{1}H NMR** (400 MHz, $CDCl_3$). δ 6.95 (d, J = 1.8 Hz, 1H), 6.80 (d, J = 8.9 Hz, 2H), 6.74 – 6.57 (m, 3H), 4.27 – 4.17 (m, 2H), 3.83 (s, 3H), 3.75 (s, 3H), 3.41 (dd, J = 5.5, 4.5 Hz, 2H), 2.51 (dd, J = 8.6, 6.7 Hz, 2H), 1.69 – 1.53 (m, 2H), 0.94 (t, J = 7.3 Hz, 3H). **^{13}C NMR** (75 MHz, $CDCl_3$) δ 153.3, 152.3, 143.1, 142.7, 140.2, 124.5, 117.3, 114.9, 112.0, 72.0, 56.1, 45.2, 37.7, 24.5, 13.8. **HRMS** (ESI^+ m/z). Calculated for $[M+H]^+$: 393.102710, found: 393.093120.

N-(2-(2-bromo-4-ethyl-6-methoxyphenoxy)ethyl)-4-methoxyaniline (**3Gc1**)

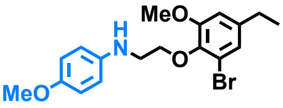

The compound was synthesized according to the General procedure (**2.3**). 4-Methoxyaniline (25 mg, 0.2 mmol) affords **3Gc1** (58 mg, 77%). A purple oil was obtained after column chromatography (SiO_2, Pentane/EtOAc 70:30). **^{1}H NMR** (400 MHz, $CDCl_3$) δ 6.98 (d, J = 1.9 Hz, 1H), 6.82 – 6.78 (m, 2H), 6.69 – 6.66 (m, 2H), 6.65 (d, J = 2.3 Hz, 1H), 4.24 – 4.21 (m, 2H), 3.84 (s, 3H), 3.76 (s, 3H), 3.43 – 3.39 (m, 2H), 2.58 (q, J = 7.6 Hz, 2H), 1.23 (t, J = 7.6 Hz, 3H). **^{13}C NMR** (75 MHz, $CDCl_3$) δ 153.2, 147.4, 142.9, 141.8, 131.9, 123.9, 117.4, 114.7, 111.5, 109.0, 71.5, 56.0, 44.0, 28.6, 15.5. **HRMS** (ESI^+ m/z). Calculated for $[M+H]^+$: 379.091011, found: 379.884302.

N-(2-(2-bromo-6-methoxy-4-propylphenoxy)ethyl)-2-methoxyaniline (**2Gc2**)

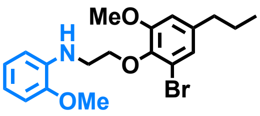

The compound was synthesized according to the General procedure (**2.3**). 2-Methoxyaniline (25 mg, 0.2 mmol) affords **2Gc2** (54 mg, 68%). A red oil was obtained after column chromatography (SiO_2, Pentane/EtOAc 70:30). **^{1}H NMR** (400 MHz, $CDCl_3$) δ 6.95 (d, J = 1.9 Hz, 1H), 6.91 – 6.84 (m, 1H), 6.81 – 6.77 (m, 1H), 6.72 – 6.62 (m, 3H), 4.30 – 4.20 (m, 2H), 3.86 (s, 3H), 3.84 (s, 3H), 3.49 (m, 2H), 2.60 – 2.37 (m, 2H), 1.62 (h, J = 7.3 Hz, 2H), 0.94 (t, J = 7.3 Hz, 3H). **^{13}C NMR** (75 MHz, $CDCl_3$) δ 147.3, 143.2, 140.1, 138.4, 124.4, 121.3, 117.5, 116.6, 112.0, 109.6, 71.8, 56.0, 43.8, 37.7, 24.5, 13.8. **HRMS** (ESI^+ m/z). Calculated for $[M+H]^+$: 393.102710, found: 393.596022.

N-(2-(2-bromo-4-ethyl-6-methoxyphenoxy)ethyl)-2-methoxyaniline (**3Gc2**)

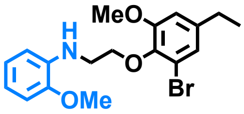

The compound was synthesized according to the General procedure (**2.3**). 2-Methoxyaniline (25 mg, 0.2 mmol) affords **3Gc2** (57 mg, 75%). A red oil was obtained after column chromatography (SiO_2, Pentane/EtOAc 70:30). **^{1}H NMR** (400 MHz, $CDCl_3$). ^{1}H NMR (300 MHz, $CDCl_3$) δ 7.00 – 6.95 (m, 1H), 6.88 (d, J = 1.5 Hz, 1H), 6.83 – 6.75 (m, 1H), 6.74 – 6.60 (m, 3H), 4.25 (m, 2H), 3.86 (d, J = 4.8 Hz, 7H), 3.49 (d, J = 4.8 Hz, 2H), 2.59 (q, J = 7.6 Hz, 2H), 1.23 (t, J = 7.6 Hz, 4H). **^{13}C NMR** (75 MHz, $CDCl_3$) δ 153.4, 147.3, 143.2, 141.6, 138.4, 123.8, 121.3, 117.5, 116.6, 111.5, 109.9, 71.8, 56.0, 55.5, 43.8, 28.6, 15.5. **HRMS** (ESI^+ m/z). Calculated for $[M+H]^+$: 379.091011, found: 379.312808.

1-(4-((2-(2-bromo-6-methoxy-4-propylphenoxy)ethyl)amino)phenyl)ethan-1-one (**2Gc3**)

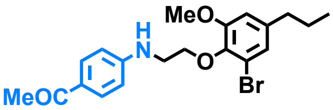

The compound was synthesized according to the General procedure (**2.3**). 4-aminoacetophenone (27 mg, 0.2 mmol) affords **2Gc3** (61 mg, 74%). A yellow oil was obtained after column chromatography (SiO_2, Pentane/EtOAc 70:30). **^{1}H NMR** (300 MHz, $CDCl_3$) δ 7.86 – 7.78 (m, 1H), 6.95 (d, J = 1.9 Hz, 1H), 6.67 (d, J = 1.9 Hz, 1H), 6.63 – 6.55 (m, 1H), 4.29 – 4.17 (m, 1H), 3.84 (d, J = 4.1 Hz, 2H), 3.51 (q, J = 4.1 Hz, 1H), 2.50 (d, J = 3.6 Hz, 3H), 1.61 (q, J = 7.5 Hz, 1H), 0.93 (t, J = 7.3 Hz, 3H). **^{13}C NMR** (75 MHz, $CDCl_3$) δ 196.4, 153.1, 152.3, 142.9, 140.4, 130.8, 126.8, 124.5, 117.3, 111.91, 71.3, 56.1, 43.3, 37.7, 25.2, 13.8. **HRMS** (ESI^+ m/z). Calculated for $[M+H]^+$: 405.602209, found: 405.329722.

1-(4-((2-(2-bromo-4-ethyl-6-methoxyphenoxy)ethyl)amino)phenyl)ethan-1-one (**3Gc3**)

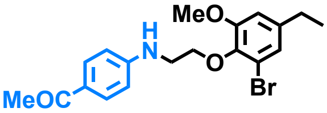

The compound was synthesized according to the General procedure (**2.3**). 4-aminoacetophenone (27 mg, 0.2 mmol) affords **3Gc3** (55 mg, 80%). A yellow oil was obtained after column chromatography (SiO_2, Pentane/EtOAc 70:30). **^{1}H NMR** (300 MHz, $CDCl_3$) δ 7.87 – 7.71 (m, 1H), 6.97 (d, J = 1.9 Hz, 1H), 6.69 (d, J = 1.9 Hz, 1H), 6.65 – 6.49 (m, 1H), 4.18 (dt, J = 19.8, 4.7 Hz, 2H), 3.85 (d, J = 3.7 Hz, 3H), 3.51 (d, J = 5.0 Hz, 2H), 2.65 – 2.53 (m, 2H), 2.49 (s, 3H), 1.2 (t, J = 7.5 Hz, 3H). **^{13}C NMR** (75 MHz, $CDCl_3$) δ 195.5, 152.3, 151.9, 146.0, 140.5, 132.3, 130.1, 125.9, 115.4, 112.9, 111.9, 70.2, 56.3, 43.3, 28.2, 26.4, 15.0. **HRMS** (ESI^+ m/z). Calculated for $[M+H]^+$: 391.213028, found: 391.953082.

4-((2-(2-bromo-6-methoxy-4-propylphenoxy)ethyl)amino)benzonitrile (**2Gc4**)

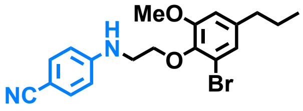

The compound was synthesized according to the General procedure (**2.3**). 4-aminobenzonitrile (24 mg, 0.2 mmol) affords **2Gc4** (50 mg, 65%). A red solid was obtained after column chromatography (SiO_2, Pentane/EtOAc 70:30). **^{1}H NMR** (300 MHz, $CDCl_3$) δ 7.49 – 7.35 (m, 2H), 6.96 (d, J = 1.9 Hz, 1H), 6.67 (d, J = 1.9 Hz, 1H), 6.65 – 6.55 (m, 2H), 4.21 (t, J = 5.0 Hz, 1H), 3.84 (s, 2H), 3.47 (q, J = 5.0 Hz, 1H), 2.51 (t, J = 7.6 Hz, 1H), 1.62 (h, J = 7.3 Hz, 2H), 0.94 (t, J = 7.3 Hz, 2H). **^{13}C NMR** (75 MHz, $CDCl_3$) δ 153.1, 151.5, 142.7, 140.6, 133.8, 124.6, 120.6, 117.3, 112.5, 98.8, 71.2, 56.1, 43.2, 37.7, 24.5, 13.8. **HRMS** (ESI$^+$ m/z). Calculated for $[M+H]^+$: 388.290210, found: 388.198062.

4-((2-(2-bromo-4-ethyl-6-methoxyphenoxy)ethyl)amino)benzonitrile (**3Gc4**)

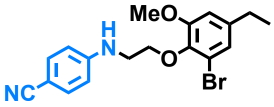

The compound was synthesized according to the General procedure (**2.3**). 4-aminobenzonitrile (24 mg, 0.2 mmol) affords **3Gc4** (53 mg, 71%). A red solid was obtained after column chromatography (SiO_2, Pentane/EtOAc 70:30). **^{1}H NMR** (300 MHz, $CDCl_3$) δ 7.41 (d, J = 8.7 Hz, 2H), 6.96 (s, 1H), 6.69 (s, 1H), 6.59 (d, J = 8.7 Hz, 2H), 4.20 (t, J = 5.0 Hz, 2H), 3.84 (s, 3H), 3.46 (q, J = 5.0 Hz, 2H), 2.58 (q, J = 7.6 Hz, 3H), 1.21 (t, J = 7.6 Hz, 3H). **^{13}C NMR** (75 MHz, $CDCl_3$) δ 153.1, 151.5, 142.7, 142.0, 133.7, 123.9, 120.6, 117.3, 112.4, 111.5, 98.7, 56.0, 43.2, 35.5. **HRMS** (ESI$^+$ m/z). Calculated for $[M+H]^+$: 374.270607, found: 375.029584.

N-(2-(2-bromo-6-methoxy-4-propylphenoxy)ethyl)-4-(methylthio)aniline (**2Gc5**)

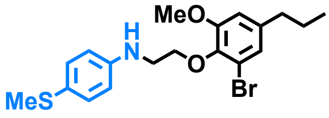

The compound was synthesized according to the General procedure (**2.3**). 4-(Methylthio)aniline (28 mg, 0.2 mmol) affords **2Gc5** (47 mg, 58%). A yellow oil was obtained after column chromatography (SiO_2, Pentane/EtOAc 70:30). **^{1}H NMR** (300 MHz, $CDCl_3$) δ 7.27 (d, J = 4.2 Hz, 1H), 7.24 (s, 0H), 6.97 (d, J = 1.9 Hz, 1H), 6.64 (d, J = 8.7 Hz, 1H), 4.28 – 4.19 (m, 1H), 3.85 (d, J = 1.7 Hz, 2H), 3.45 (t, J = 5.0 Hz, 1H), 2.61 – 2.47 (m, 2H), 2.43 (d, J = 1.3 Hz, 2H), 1.63 (tq, J = 10.9, 7.3, 5.2 Hz, 2H), 0.96 (t, J = 7.3 Hz, 2H). **^{13}C NMR** (75 MHz, $CDCl_3$) δ 153.2, 147.4, 143.0, 140.3, 131.6, 124.5, 117.3, 113.8, 112.0, 71.7, 56.1, 44.1, 37.7, 24.5, 19.3, 13.8. **HRMS** (ESI$^+$ m/z). Calculated for $[M+H]^+$: 411.374320, found: 411.213138.

N-(2-(2-bromo-4-ethyl-6-methoxyphenoxy)ethyl)-4-(methylthio)aniline (**3Gc5**)

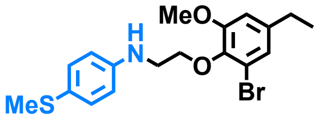

The compound was synthesized according to the General procedure (**2.3**). 4-(Methylthio)aniline (28 mg, 0.2 mmol) affords **3Gc5** (52 mg, 66%). A dark yellow oil was obtained after column chromatography (SiO_2, Pentane/EtOAc 70:30). **^{1}H NMR** (300 MHz, $CDCl_3$) δ 7.23 (d, J = 8.6 Hz, 2H), 6.97 (d, J = 1.9 Hz, 1H), 6.68 (d, J = 1.9 Hz, 1H), 6.62 (d, J = 8.6 Hz, 2H), 4.24 – 4.19 (m, 2H), 3.83 (s, 3H), 3.43 (t, J = 5.0 Hz, 2H), 2.58 (q, J = 7.6 Hz, 2H), 2.41 (s, 3H), 1.22 (t, J = 7.6 Hz, 3H). **^{13}C NMR** (75 MHz, $CDCl_3$) δ 153.3, 147.3, 143.0, 141.8, 131.6, 124.4, 123.9, 117.4, 113.8, 111.6, 71.7, 56.1, 44.1, 28.6, 19.3, 15.5. **HRMS** (ESI$^+$ m/z). Calculated for $[M+H]^+$: 397.860593, found: 397.110634.

N-(2-(2-bromo-6-methoxy-4-propylphenoxy)ethyl)-4-fluoroaniline (**2Gc6**)

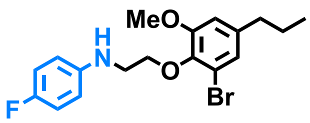

The compound was synthesized according to the General procedure (**2.3**). 4-Fluoroaniline (22 mg, 0.2 mmol) affords **2Gc6** (65 mg, 85%). A red oil was obtained after column chromatography (SiO_2, Pentane/EtOAc 70:30). **^{1}H NMR** (300 MHz, $CDCl_3$) δ 7.00 – 6.85 (m, 3H), 6.68 – 6.56 (m, 3H), 4.27 – 4.17 (m, 2H), 3.83 (s, 3H), 3.45 – 3.36 (m, 2H), 2.56 – 2.46 (m, 2H), 1.62 (dq, J = 14.7, 7.4 Hz, 2H), 0.95 (t, J = 7.4 Hz, 3H). **^{13}C NMR** (75 MHz, $CDCl_3$) δ 157.5, 154.4, 153.2, 144.8, 143.0, 140.3, 124.5, 117.4, 115.8, 115.5, 114.1 (d, $J_{C\text{-}F}$ = 7.4 Hz), 112.0, 56.0, 37.7, 24.5, 13.8. **HRMS** (ESI^+ m/z). Calculated for $[M+H]^+$: 381.097384, found: 381.911068.

N-(2-(2-bromo-4-ethyl-6-methoxyphenoxy)ethyl)-4-fluoroaniline (**3Gc6**)

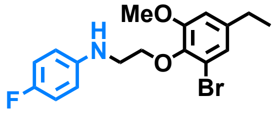

The compound was synthesized according to the General procedure (**2.3**). 4-Fluoroaniline (22 mg, 0.2 mmol) affords **3Gc6** (58 mg, 80%). A red oil was obtained after column chromatography (SiO_2, Pentane/EtOAc 70:30). **^{1}H NMR** (300 MHz, $CDCl_3$) δ 7.00 – 6.85 (m, 3H), 6.69 (d, J = 1.9 Hz, 1H), 6.65 – 6.58 (m, 2H), 4.25 – 4.18 (m, 2H), 3.84 (s, 3H), 3.40 (d, J = 10.0 Hz, 2H), 2.65 – 2.52 (m, 2H), 1.22 (t, J = 7.6 Hz, 3H). **^{13}C NMR** (75 MHz, $CDCl_3$) δ 153.3, 144.8, 143.0, 141.8, 123.9, 117.4, 115.8, 115.5, 114.1 (d, $J_{C\text{-}F}$ = 7.5 Hz), 114.0, 111.5, 71.7, 56.1, 44.8, 28.6, 15.5. **HRMS** (ESI^+ m/z). Calculated for $[M+H]^+$: 368.250613, found: 368.149534.

N-(2-(2-bromo-6-methoxy-4-propylphenoxy)ethyl)-3-fluoroaniline (**2Gc7**)

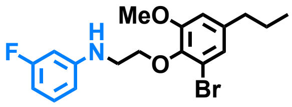

The compound was synthesized according to the General procedure (**2.3**). 3-Fluoroaniline (22 mg, 0.2 mmol) affords **2Gc7** (68 mg, 89%). A light-red oil was obtained after column chromatography (SiO_2, Pentane/EtOAc 70:30). **^{1}H NMR** (300 MHz, $CDCl_3$) δ 7.41 (m, 1H), 6.99 (m, 1H), 6.75 – 6.61 (m, 1H), 4.27 – 4.18 (m, 1H), 3.84 (s, 1H), 3.48 (m, 1H), 2.52 (m, 1H), 1.61 (m, 1H), 0.95 (m, 3H). **^{13}C NMR** (75 MHz, $CDCl_3$) δ 153.2, 150.9, 142.9, 140.4, 126.7, 126.5, 124.6, 119.4, 117.3, 112.1 (d, $J_{C\text{-}F}$ = 7.5 Hz), 71.4, 56.1, 43.6, 37.7, 24.5, 13.8. **HRMS** (ESI^+ m/z). Calculated for $[M+H]^+$: 381.097384, found: 381.606421.

N-(2-(2-bromo-4-ethyl-6-methoxyphenoxy)ethyl)-3-fluoroaniline (**3Gc7**)

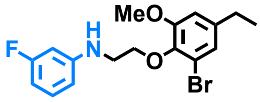

The compound was synthesized according to the General procedure (**2.3**). 3-Fluoroaniline (22 mg, 0.1 mmol) affords **3Gc7** (70 mg, 92%). A light-red oil was obtained after column chromatography (SiO_2, Pentane/EtOAc 70:30). **^{1}H NMR** (300 MHz, $CDCl_3$) δ 7.05 (td, J = 8.1, 6.8 Hz, 1H), 6.93 (d, J = 1.9 Hz, 1H), 6.64 (d, J = 1.9 Hz, 1H), 6.41 – 6.27 (m, 3H), 4.20 – 4.14 (m, 2H), 3.79 (s, 3H), 3.37 (t, J = 5.0 Hz, 2H), 2.54 (q, J = 7.6 Hz, 2H), 1.18 (t, J = 7.6 Hz, 3H). **^{13}C NMR** (75 MHz, $CDCl_3$) δ 165.8, 153.2, 150.3, 130.3, 123.8, 117.3, 111.5, 109.0, 103.8, 99.7 (d, $J_{C\text{-}F}$ = 7.5 Hz),, 71.5, 55.9, 43.9, 28.5, 15.4. **HRMS** (ESI^+ m/z). Calculated for $[M+H]^+$: 368.250613, found: 368.149534.

N-(2-(2-bromo-6-methoxy-4-propylphenoxy)ethyl)-4-(trifluoromethyl)aniline (**2Gc8**)

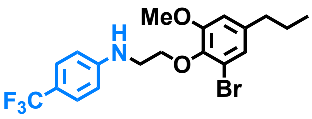

The compound was synthesized according to the General procedure (**2.3**). 4-(trifluoromethyl) aniline (32 mg, 0.2 mmol) affords **2Gc8** (66 mg, 77%). A purple oil was obtained after column chromatography (SiO_2, Pentane/EtOAc 70:30). **^{1}H NMR** (300 MHz, $CDCl_3$) δ 7.41 (d, J = 8.4 Hz, 1H), 6.96 (d, J = 1.8 Hz, 2H), 6.75 – 6.61 (m, 2H), 4.22 (t, J = 5.2 Hz, 1H), 3.84 (s, 3H), 3.48 (q, J = 5.2 Hz, 1H), 2.60 – 2.46 (m, 1H), 1.68 – 1.59 (m, 1H), 0.94 (t, J = 7.3 Hz, 3H). **^{13}C NMR** (75 MHz, $CDCl_3$) δ 165.8, 162.6, 153.2, 150.2, 143.0, 140.3, 130.4, 124.5, 117.3, 112.1, 109.1 (d, J = 2.3 Hz), 103.8 (d, J = 21.5 Hz), 99.7 (d, J = 25.2 Hz), 71.6, 56.0, 44.0, 37.7, 24.5, 13.8. **HRMS** (ESI^+ m/z). Calculated for $[M+H]^+$: 432.201621, found: 432.083528.

N-(2-(2-bromo-4-ethyl-6-methoxyphenoxy)ethyl)-4-(trifluoromethyl)aniline (**3Gc8**)

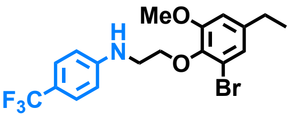

The compound was synthesized according to the General procedure (**2.3**). 4-(trifluoromethyl) aniline (32 mg, 0.2 mmol) affords **2Gc8** (66 mg, 79%). A purple oil was obtained after column chromatography (SiO_2, Pentane/EtOAc 70:30). **^{1}H NMR** (300 MHz, $CDCl_3$) δ 7.36 (d, J = 8.3 Hz, 2H), 6.93 (s, 1H), 6.65 (s, 1H), 6.60 (d, J = 8.3 Hz, 2H), 4.20 – 4.15 (m, 2H), 3.79 (s, 3H), 3.42 (t, J = 5.0 Hz, 2H), 2.54 (q, J = 7.6 Hz, 2H), 1.18 (t, J = 7.6 Hz, 3H). **^{13}C NMR** (75 MHz, $CDCl_3$) δ 153.2, 150.9, 142.8, 141.9, 141.6, 126.5, 123.8, 118.9, 118.4, 112.1, 111.4, 71.4, 55.9, 43.5, 28.5. **HRMS** (ESI^+ m/z). Calculated for $[M+H]^+$: 417.369613, found: 417.163534.

8-methoxy-4-(4-methoxyphenyl)-6-propyl-3,4-dihydro-2H-benzo[b][1,4]oxazine (**2Gd1**)

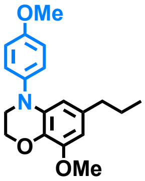

The compound was synthesized according to the General procedure (**2.4**). **2Gc1** (39 mg, 0.1 mmol) affords **2Gd1** (24 mg, 79%). A dark-red oil was obtained after column chromatography (SiO_2, Pentane/EtOAc 70:30). **^{1}H NMR** (300 MHz, $CDCl_3$) δ 7.20 – 7.11 (m, 1H), 6.96 – 6.86 (m, 1H), 6.22 (d, J = 1.9 Hz, 1H), 6.16 (d, J = 1.9 Hz, 1H), 4.34 (dd, J = 5.0, 3.6 Hz, 1H), 3.88 (s, 2H), 3.82 (s, 2H), 3.68 – 3.59 (m, 1H), 2.35 (dd, J = 9.1, 6.6 Hz, 1H), 1.51 (dt, J = 9.1, 6.6 Hz, 1H), 0.88 (t, J = 7.3 Hz, 3H). **^{13}C NMR** (75 MHz, $CDCl_3$) δ 156.6, 148.6, 140.8, 134.6, 133.9, 131.9, 126.3, 114.8, 109.2, 102.8, 64.6, 56.0, 55.6, 49.8, 38.2, 24.9, 14.0. **HRMS** (ESI^+ m/z). Calculated for $[M+H]^+$: 313.182652, found: 314.022981.

6-ethyl-8-methoxy-4-(4-methoxyphenyl)-3,4-dihydro-2H-benzo[b][1,4]oxazine (**3Gd1**)

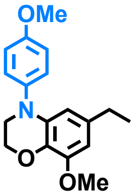

The compound was synthesized according to the General procedure (**2.4**). **3Gc1** (38 mg, 0.1 mmol) affords **3Gd1** (23 mg, 77%). A dark-red oil was obtained after column chromatography (SiO_2, Pentane/EtOAc 70:30). **^{1}H NMR** (300 MHz, $CDCl_3$) δ 7.16 (d, J = 8.9 Hz, 2H), 6.91 (d, J = 8.9 Hz, 2H), 6.24 (d, J = 1.9 Hz, 1H), 6.18 (d, J = 1.9 Hz, 1H), 4.37 – 4.31 (m, 2H), 3.88 (s, 3H), 3.82 (s, 3H), 3.66 – 3.62 (m, 2H), 2.42 (q, J = 7.6 Hz, 2H), 1.12 (t, J = 7.6 Hz, 3H). **^{13}C NMR** (75 MHz, $CDCl_3$) δ 156.6, 148.6, 140.7, 136.2, 134.0, 131.8, 126.4, 114.8, 108.4, 102.2, 64.6, 56.0, 55.6, 49.7, 29.0, 15.9. **HRMS** (ESI^+ m/z). Calculated for $[M+H]^+$: 299.162532 , found: 300.283226.

8-methoxy-4-(2-methoxyphenyl)-6-propyl-3,4-dihydro-2H-benzo[b][1,4]oxazine (**2Gd2**)

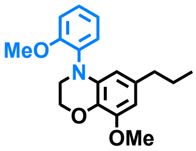

The compound was synthesized according to the General procedure (**2.4**). **2Gc2** (39 mg, 0.1 mmol) affords **2Gd2** (28 mg, 90%). A dark-red oil was obtained after column chromatography (SiO_2, Pentane/EtOAc 70:30). **^{1}H NMR** (300 MHz, $CDCl_3$) δ 6.96 (d, J = 1.9 Hz, 1H), 6.88 (td, J = 7.6, 1.5 Hz, 1H), 6.79 (dd, J = 7.6, 1.5 Hz, 1H), 6.80 – 6.60 (m, 3H), 4.26 (t, J = 5.2 Hz, 2H), 3.87 (s, 3H), 3.85 (s, 3H), 3.50 (t, J = 5.2 Hz, 2H), 2.52 (dd, J = 8.6, 6.7 Hz, 2H), 1.72 – 1.54 (m, 2H), 0.95 (t, J = 7.6 Hz, 3H). **^{13}C NMR** (75 MHz, $CDCl_3$) δ 153.4, 147.3, 143.3, 140.1, 138.4, 124.5, 121.3, 117.0, 112.6, 110.0, 109.7, 107.0, 71.8, 55.8, 43.8, 37.7, 24.5, 13.8. **HRMS** (ESI^+ m/z). Calculated for $[M+H]^+$: 313.182652, found: 313.962145

6-ethyl-8-methoxy-4-(2-methoxyphenyl)-3,4-dihydro-2H-benzo[b][1,4]oxazine (**3Gd2**)

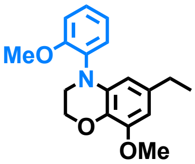

The compound was synthesized according to the General procedure (**2.4**). **3Gc2** (37.9 mg, 0.1 mmol) affords **3Gd2** (25 mg, 82%). A purple oil was obtained after column chromatography (SiO_2, Pentane/EtOAc 70:30). **^{1}H NMR** (300 MHz, $CDCl_3$) δ 7.27 – 7.14 (m, 1H), 6.96 (ddd, J = 15.2, 8.1, 1.9 Hz, 1H), 6.22 (d, J = 1.9 Hz, 1H), 6.01 (d, J = 1.9 Hz, 1H), 4.34 – 4.32 (m, 1H), 3.88 (s, 2H), 3.83 (s, 3H), 3.66 – 3.50 (m, 2H), 1.09 (t, J = 7.6 Hz, 3H). **^{13}C NMR** (75 MHz, $CDCl_3$) δ 155.3, 151.1, 149.0, 138.0, 135.4, 129.0, 125.9, 121.2, 113.5, 108.5, 102.4, 64.6, 56.0, 55.7, 48.3, 29.4, 16.0. **HRMS** (ESI^+ m/z). Calculated for $[M+H]^+$: 299.162532 , found: 299.742676.

1-(4-(8-methoxy-6-propyl-2,3-dihydro-4H-benzo[b][1,4]oxazin-4-yl)phenyl)ethan-1-one (**2Gd3**)

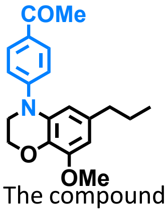

The compound was synthesized according to the General procedure (**2.4**). **2Gc3** (40 mg, 0.1 mmol) affords **2Gd3** (21 mg, 67%). A yellow oil was obtained after column chromatography (SiO_2, Pentane/EtOAc 70:30). **^{1}H NMR** (300 MHz, $CDCl_3$) δ 7.84 (d, J = 8.8 Hz, 1H), 6.96 (d, J = 1.9 Hz, 1H), 6.67 (d, J = 1.9 Hz, 1H), 6.61 (d, J = 8.8 Hz, 1H), 4.22 (t, J = 5.1 Hz, 1H), 3.84 (s, 1H), 3.51 (q, J = 5.1 Hz, 1H), 2.63 – 2.35 (m, 2H), 1.62 (d, J = 7.6 Hz, 3H), 0.94 (t, J = 7.3 Hz, 3H). **^{13}C NMR** (75 MHz, $CDCl_3$) δ 196.5, 153.1, 152.3, 142.8, 140.5, 130.9, 126.8, 124.5, 117.3, 112.0, 111.7, 71.4, 56.1, 37.7, 26.1, 24.5, 13.8. **HRMS** (ESI^+ m/z). Calculated for $[M+H]^+$: 325.176410, found: 325.886745.

1-(4-(6-ethyl-8-methoxy-2,3-dihydro-4H-benzo[b][1,4]oxazin-4-yl)phenyl)ethan-1-one (**3Gd3**)

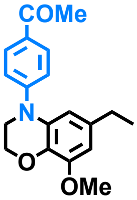

The compound was synthesized according to the General procedure (**2.4**). **3Gc3** (39 mg, 0.1 mmol) affords **3Gd3** (20 mg, 64%). A yellow oil was obtained after column chromatography (SiO_2, Pentane/EtOAc 70:30). **^{1}H NMR** (300 MHz, $CDCl_3$) δ 7.89 – 7.76 (m, 1H), 6.98 (d, J = 1.9 Hz, 1H), 6.69 (d, J = 1.9 Hz, 1H), 6.67 – 6.56 (m, 1H), 4.22 (dd, J = 5.5, 4.4 Hz, 1H), 3.85 (s, 1H), 3.52 (q, J = 5.5 Hz, 1H), 2.65 – 2.49 (m, 2H), 1.22 (t, J = 7.6 Hz, 3H). **^{13}C NMR** (75 MHz, $CDCl_3$) δ 196.5, 152.8, 142.0, 130.9, 126.9, 124.0, 117.4, 111.7, 111.6, 71.4, 56.1, 43.4, 28.6, 26.1, 15.5. **HRMS** (ESI^+ m/z). Calculated for $[M+H]^+$: 311.396232 , found: 311.269776.

4-(8-methoxy-6-propyl-2,3-dihydro-4H-benzo[b][1,4]oxazin-4-yl)benzonitrile (**2Gd4**)

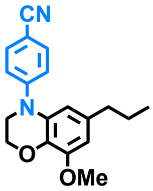

The compound was synthesized according to the General procedure (**2.4**). **2Gc4** (40 mg, 0.1 mmol) affords **2Gd4** (26 mg, 84%). A red oil was obtained after column chromatography (SiO_2, Pentane/EtOAc 70:30). **^{1}H NMR** (300 MHz, $CDCl_3$) δ 7.44 (d, J = 8.7 Hz, 1H), 6.96 (d, J = 1.9 Hz, 1H), 6.71 – 6.59 (m, 2H), 4.21 (dd, J = 5.5, 4.4 Hz, 1H), 3.84 (s, 2H), 3.52 – 3.43 (m, 2H), 2.52 (dd, J = 8.7, 7.3 Hz, 2H), 1.71 – 1.54 (m, 2H), 0.94 (t, J = 7.3 Hz, 3H). **^{13}C NMR** (75 MHz, $CDCl_3$) δ 150.6, 148.8, 139.4, 137.6, 136.2, 132.7, 118.2, 117.0, 112.7, 105.2, 103.8, 64.6, 56.6, 42.4, 36.4, 24.5, 13.6. **HRMS** (ESI^+ m/z). Calculated for $[M+H]^+$: 308.283212 , found: 309.071224.

4-(6-ethyl-8-methoxy-2,3-dihydro-4H-benzo[b][1,4]oxazin-4-yl)benzonitrile (**3Gd4**)

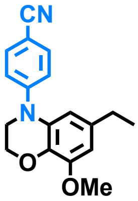

The compound was synthesized according to the General procedure (**2.4**). **3Gc4** (37 mg, 0.1 mmol) affords **3Gd4** (23 mg, 80%). A red oil was obtained after column chromatography (SiO_2, Pentane/EtOAc 70:30). **^{1}H NMR** (300 MHz, $CDCl_3$) δ 7.61 – 7.50 (m, 1H), 7.25 – 7.19 (m, 1H), 6.58 (d, J = 1.9 Hz, 1H), 6.42 (d, J = 1.9 Hz, 1H), 4.36 – 4.27 (m, 1H), 3.90 (d, J = 4.0 Hz, 2H), 3.81 – 3.72 (m, 1H), 2.58 – 2.40 (m, 1H), 1.17 (t, J = 7.6 Hz, 3H). **^{13}C NMR** (75 MHz, $CDCl_3$) δ 150.8, 149.2, 136.1, 133.6, 129.0, 120.6, 111.0, 105.4, 104.0, 64.8, 56.1, 47.8, 28.9, 15.9. **HRMS** (ESI^+ m/z). Calculated for $[M+H]^+$: 294.142632 , found: 294.296671.

4-(4-fluorophenyl)-8-methoxy-6-propyl-3,4-dihydro-2H-benzo[b][1,4]oxazine (**2Gd6**)

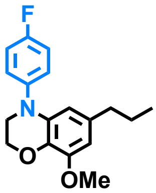

The compound was synthesized according to the General procedure (**2.4**). **3Gc6** (38 mg, 0.1 mmol) affords **3Gd7** (25 mg, 85%). A dark-red oil was obtained after column chromatography (SiO_2, Pentane/EtOAc 70:30). **^{1}H NMR** (300 MHz, $CDCl_3$) δ 7.22 – 7.14 (m, 2H), 7.10 – 7.00 (m, 2H), 6.24 (dd, J = 14.7, 1.9 Hz, 2H), 4.36 – 4.30 (m, 2H), 3.88 (s, 3H), 3.68 – 3.62 (m, 2H), 2.42 – 2.34 (m, 2H), 1.60 – 1.47 (m, 2H), 0.88 (d, J = 14.7 Hz, 3H). **^{13}C NMR** (75 MHz, $CDCl_3$) δ 157.8, 143.8, 132.9, 132.3, 126.0, 116.1 (d, J_{C-F} = 22.4 Hz),, 109.6, 103.5, 64.4, 56.0, 49.6, 38.2, 24.8, 13.9. **HRMS** (ESI^+ m/z). Calculated for $[M+H]^+$: 301.156212 , found: 302.154884.

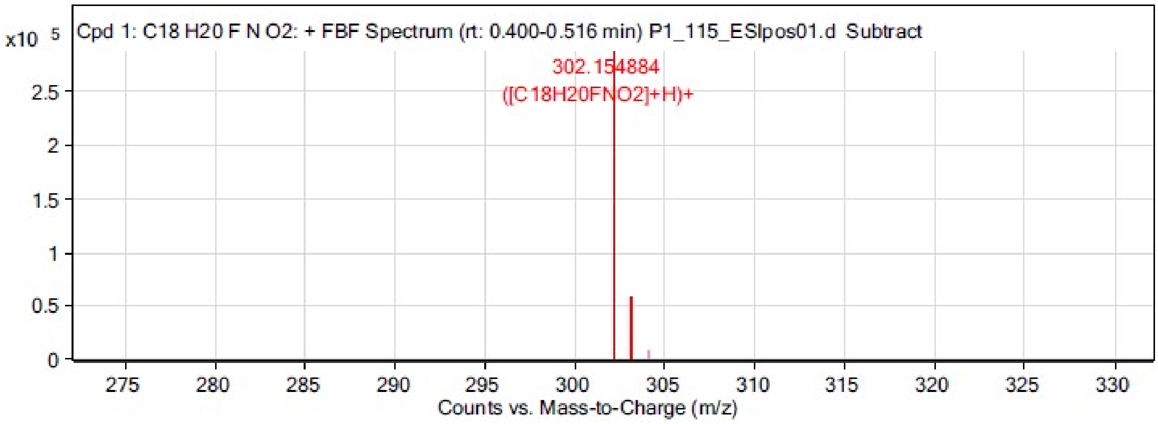

6-ethyl-4-(4-fluorophenyl)-8-methoxy-3,4-dihydro-2H-benzo[b][1,4]oxazine (**3Gd6**)

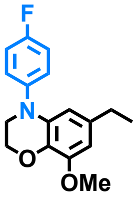

The compound was synthesized according to the General procedure (**2.4**). **3Gc6** (36 mg, 0.1 mmol) affords **3Gd7** (23 mg, 81%). A dark-purple oil was obtained after column chromatography (SiO_2, Pentane/EtOAc 70:30). **^{1}H NMR** (300 MHz, CDCl3) δ 7.22 – 7.14 (m, 2H), 7.10 – 6.98 (m, 2H), 6.26 (dd, J = 15.0, 1.9 Hz, 2H), 4.37 – 4.30 (m, 2H), 3.89 (s, 3H), 3.70 – 3.61 (m, 2H), 2.44 (q, J = 7.6 Hz, 2H), 1.12 (t, J = 7.6 Hz, 3H). **^{13}C NMR** (75 MHz, $CDCl_3$) δ 148.8, 136.3, 133.1, 132.2, 126.1, 125.9, 116.1 (d, J_{C-F} = 22.5 Hz),, 108.8, 102.9, 64.5, 56.0, 49.6, 29.0, 15.9. **HRMS** (ESI^+ m/z). Calculated for $[M+H]^+$: 287.736511 , found: 287.968814.

4-(3-fluorophenyl)-8-methoxy-6-propyl-3,4-dihydro-2H-benzo[b][1,4]oxazine (**2Gd7**)

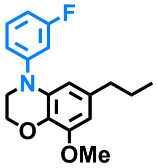

The compound was synthesized according to the General procedure (**2.4**). **2Gc7** (38 mg, 0.1 mmol) affords **2Gd8** (27 mg, 90%). A dark-red oil was obtained after column chromatography (SiO_2, Pentane/EtOAc 70:30). **^{1}H NMR** (300 MHz, $CDCl_3$) δ 7.33 – 7.19 (m, 1H), 7.02 – 6.84 (m, 2H), 6.79 – 6.66 (m, 1H), 6.47 (d, J = 1.9 Hz, 1H), 6.32 (d, J = 1.9 Hz, 1H), 4.36 – 4.26 (m, 1H), 3.92 – 3.86 (m, 3H), 3.75 – 3.66 (m, 2H), 2.41 (dd, J = 8.9, 6.6 Hz, 2H), 1.55 (dt, J = 8.9, 7.4 Hz, 2H), 0.90 (t, J = 7.4 Hz, 3H). **^{13}C NMR** (75 MHz, $CDCl_3$) δ 165.2, 150.7, 147.4, 139.1, 136.1, 130.5, 116.8, 112.9, 109.4, 107.4, 105.2 (d, J_{C-F} = 23.0 Hz), 64.7, 56.3, 42.4, 36.9, 24.5, 13.9. **HRMS** (ESI^+ m/z). Calculated for $[M+H]^+$: 301.156212 , found: 301.225811.

6-ethyl-4-(3-fluorophenyl)-8-methoxy-3,4-dihydro-2H-benzo[b][1,4]oxazine (**3Gd7**)

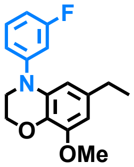

The compound was synthesized according to the General procedure (**2.4**). **3Gc7** (36 mg, 0.1 mmol) affords **3Gd8** (25 mg, 89%). A purple oil was obtained after column chromatography (SiO_2, Pentane/EtOAc 70:30). **^{1}H NMR** (300 MHz, $CDCl_3$) δ 7.33 – 7.19 (m, 1H), 7.03 – 6.93 (m, 1H), 6.90 (d, J = 2.3 Hz, 1H), 6.73 (dd, J = 2.3, 1H), 6.49 (d, J = 1.8 Hz, 1H), 6.34 (d, J = 1.8 Hz, 1H), 4.35 – 4.26 (m, 2H), 3.90 (s, 2H), 3.76 – 3.66 (m, 2H), 2.48 (q, J = 7.6 Hz, 2H), 1.15 (t, J = 7.6 Hz, 3H). **^{13}C NMR** (75 MHz, $CDCl_3$) δ 153.3, 150.3, 141.9, 130.4, 124.0, 117.4, 111.6, 109.1, 103.7, 99.75 (d, J_{C-F} = 25.0 Hz), 71.6, 44.0, 28.6, 15.5. **HRMS** (ESI^+ m/z). Calculated for $[M+H]^+$: 287.736511 , found: 287.196922.

4. NMR Spectra

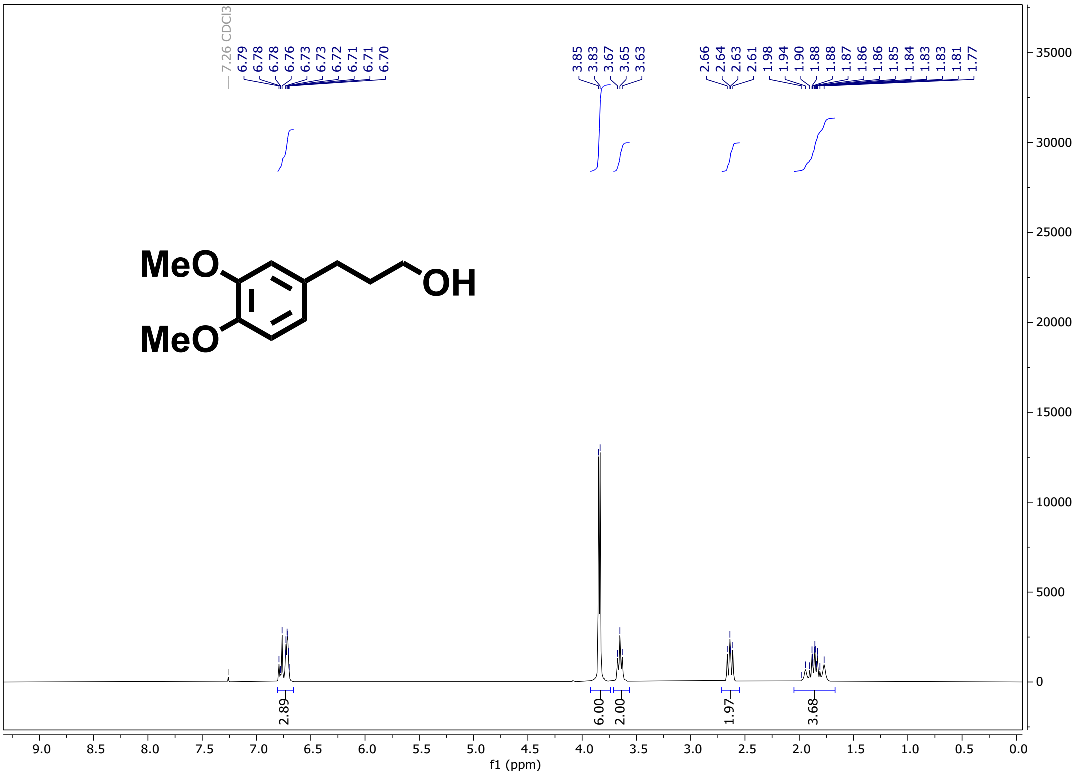

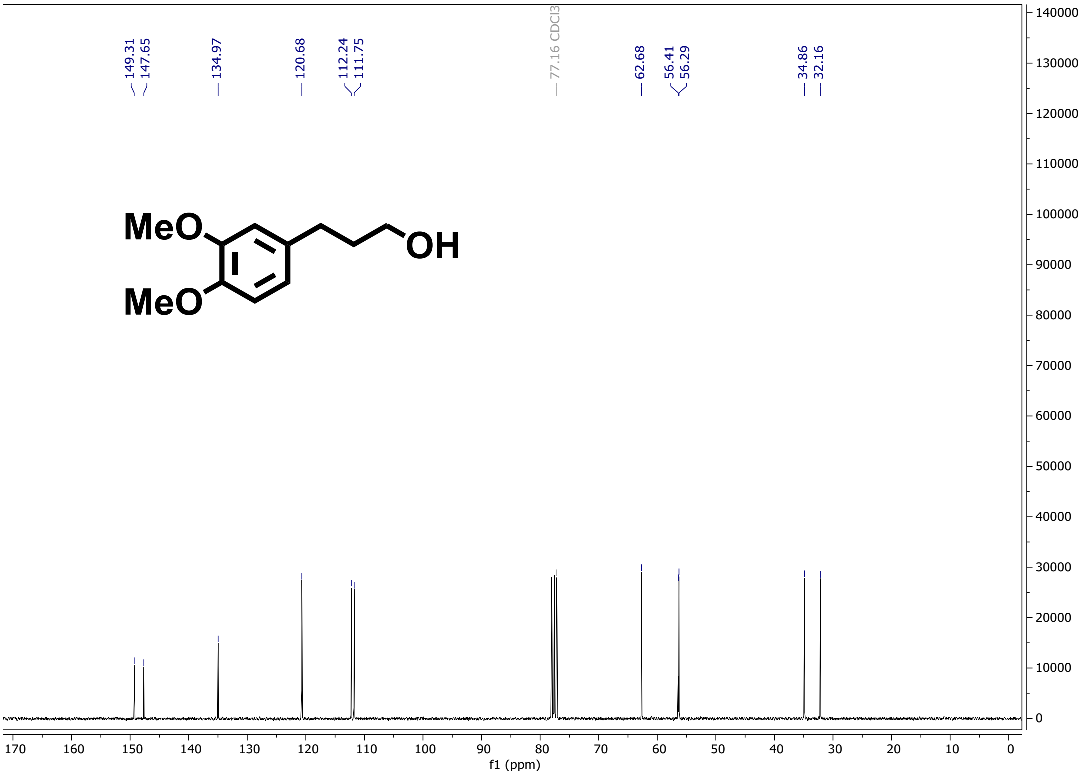

Fig. S5 ^{1}H and ^{13}C NMR spectra of compound **1Ga**.

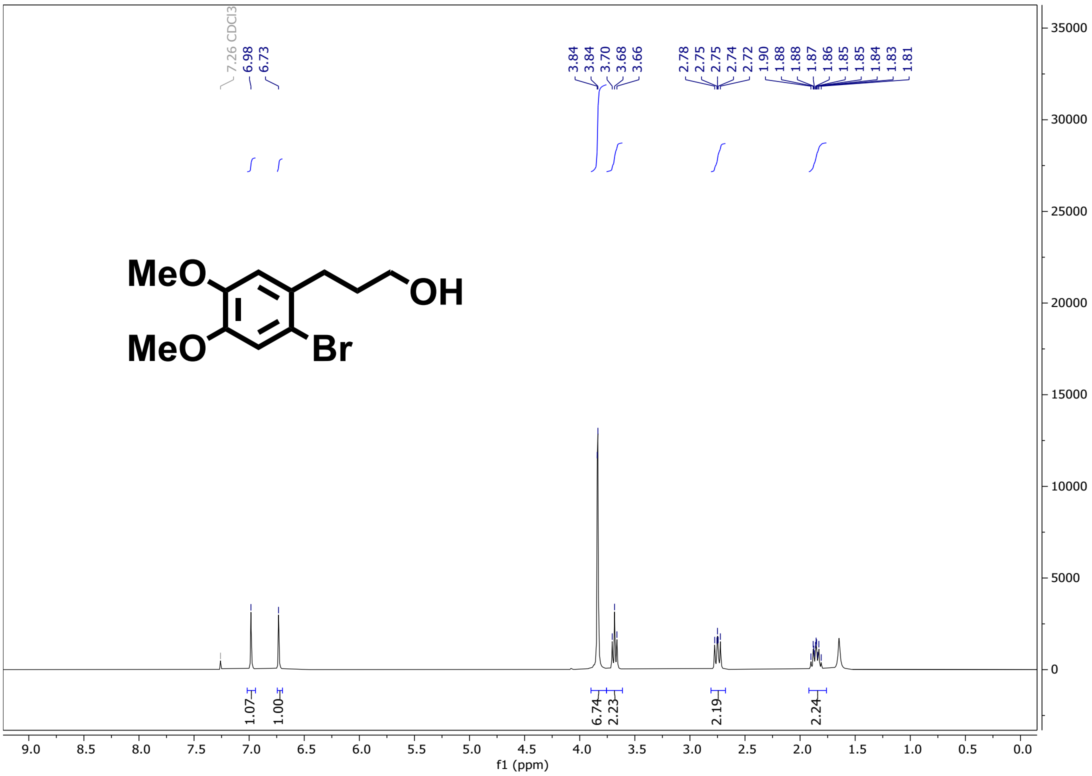

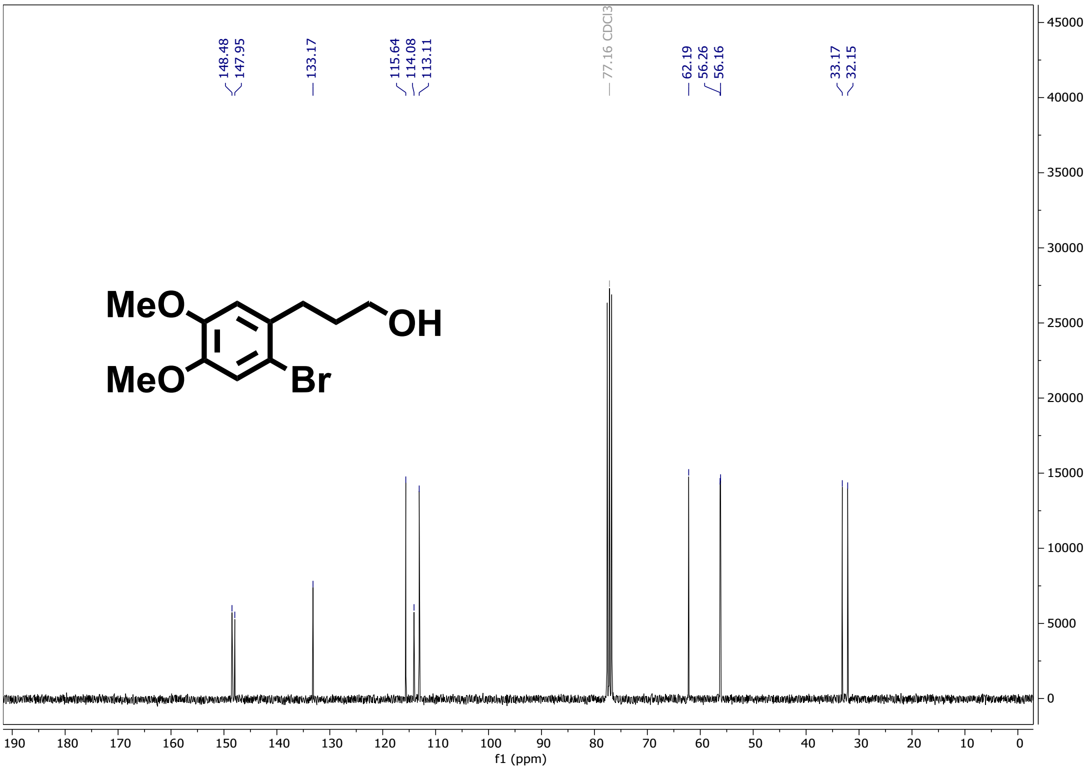

Fig. S6 ^{1}H and ^{13}C NMR spectra of compound **1Gb**.

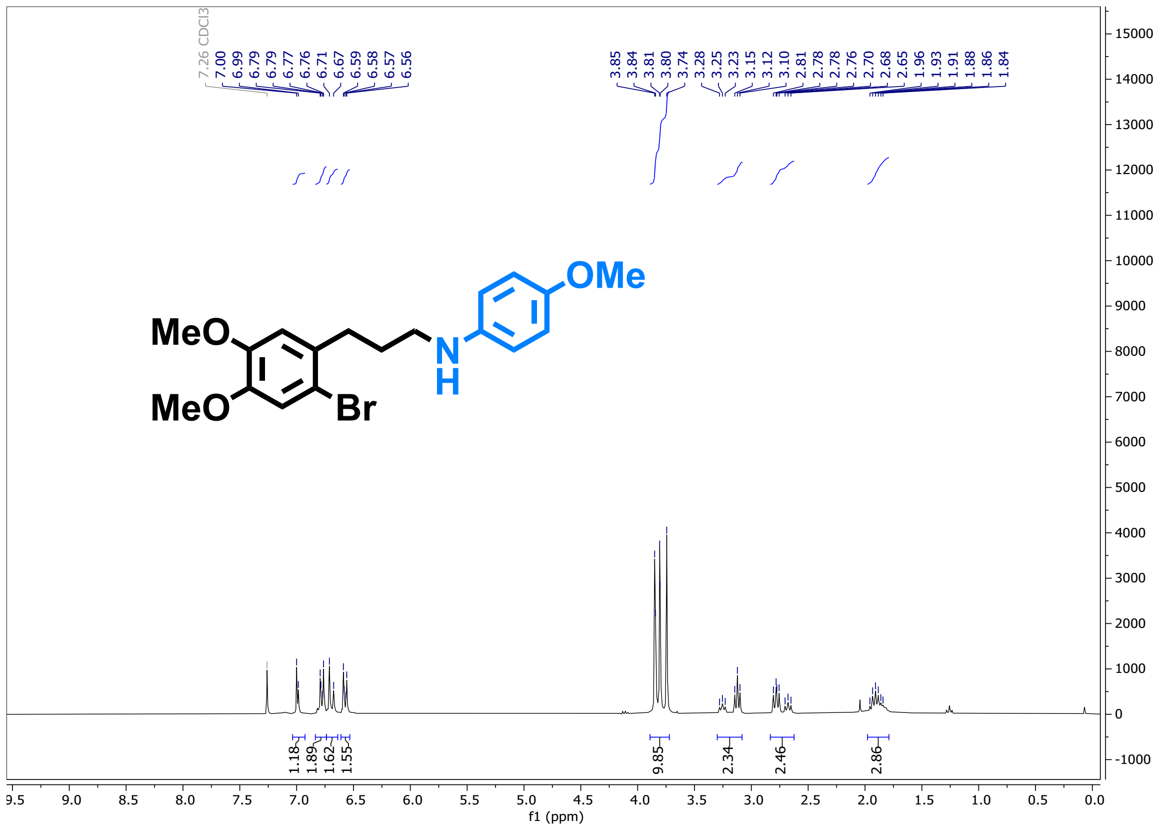

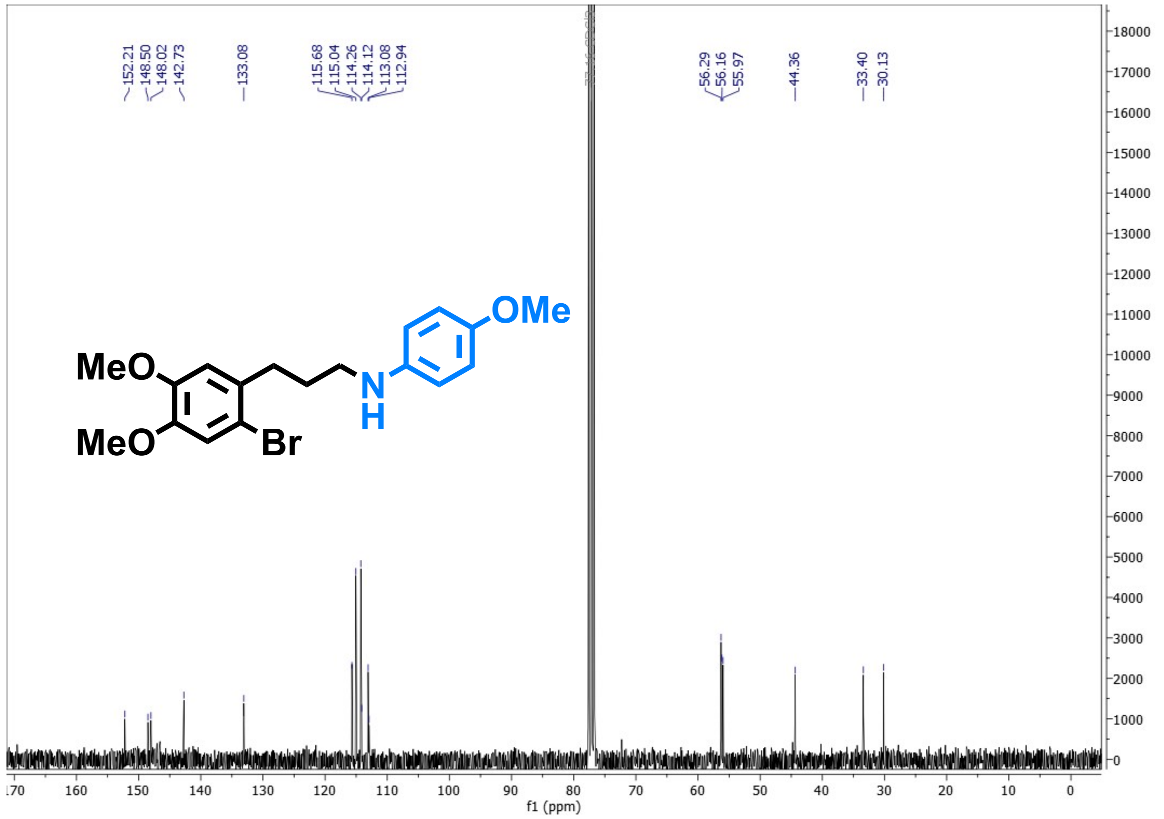

Fig. S7 ^{1}H and ^{13}C NMR spectra of compound **1Gc1**.

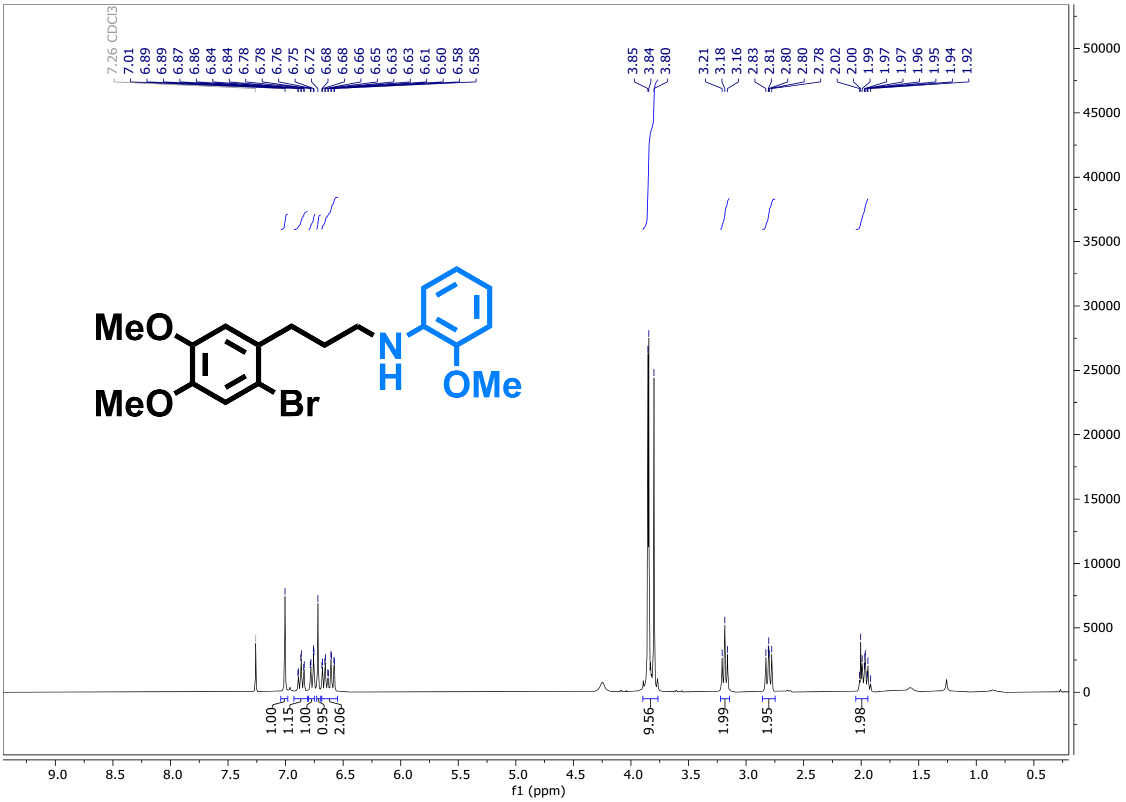

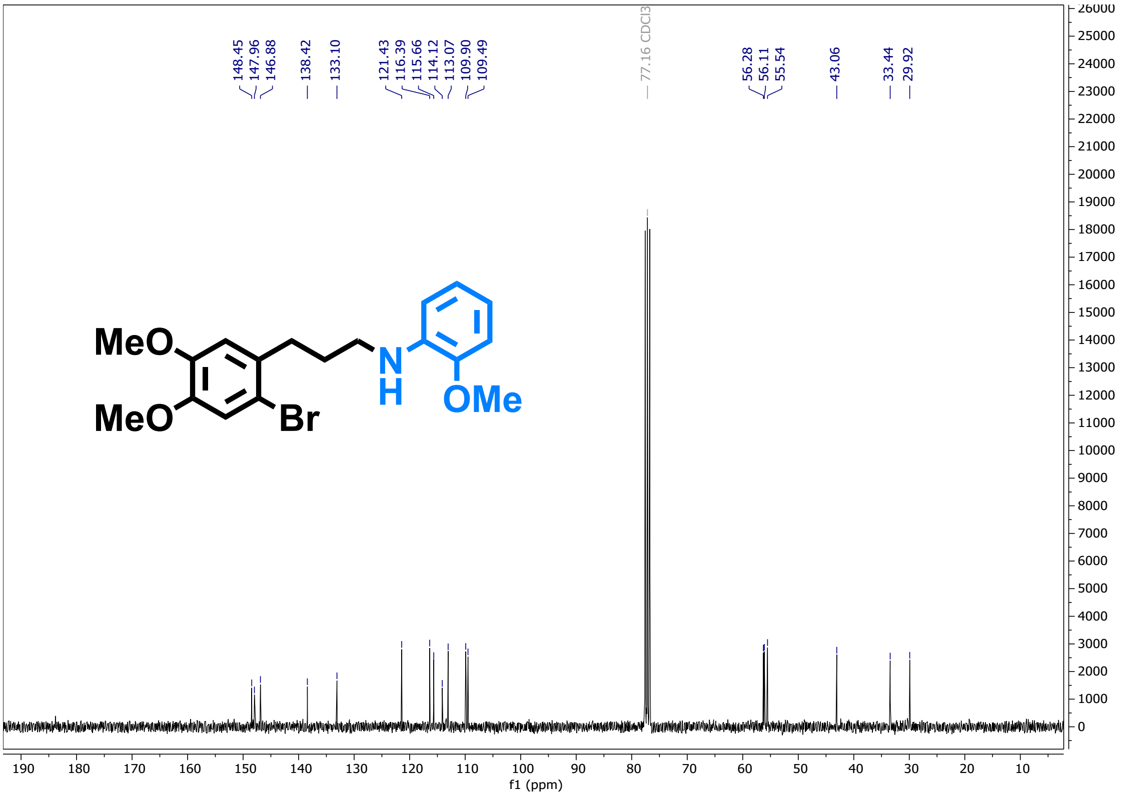

Fig. S8 ^{1}H and ^{13}C NMR spectra of compound **1Gc2**.

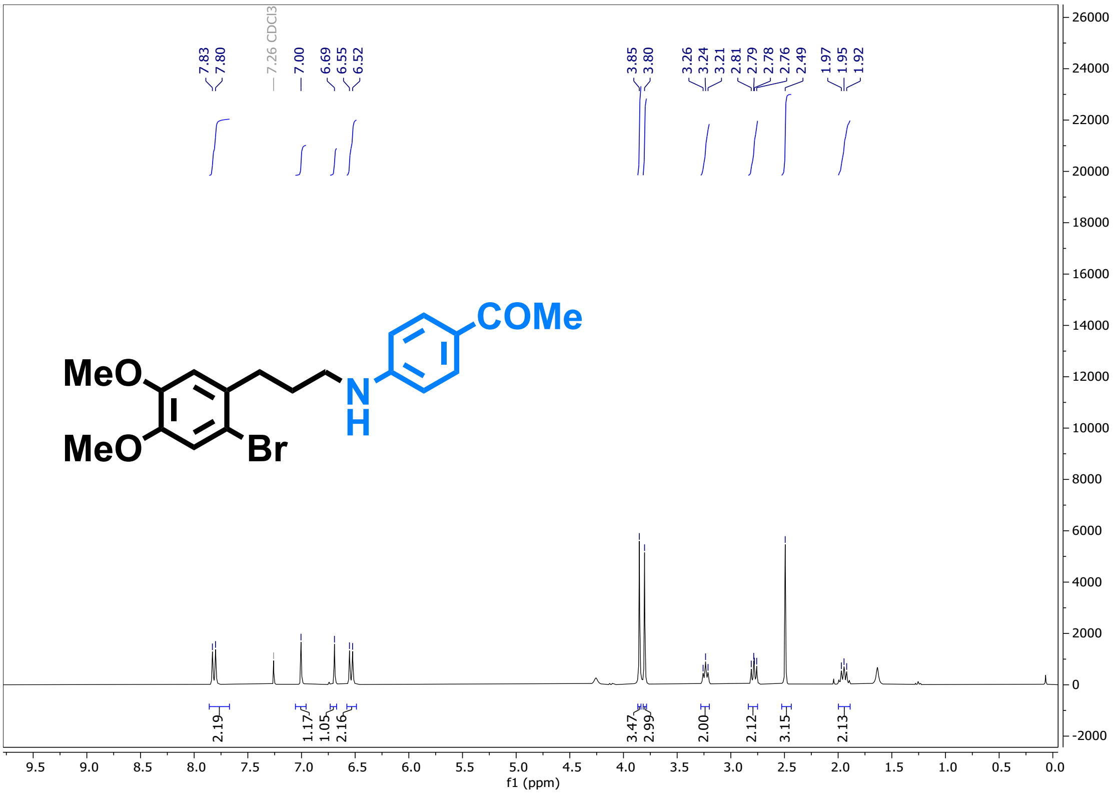

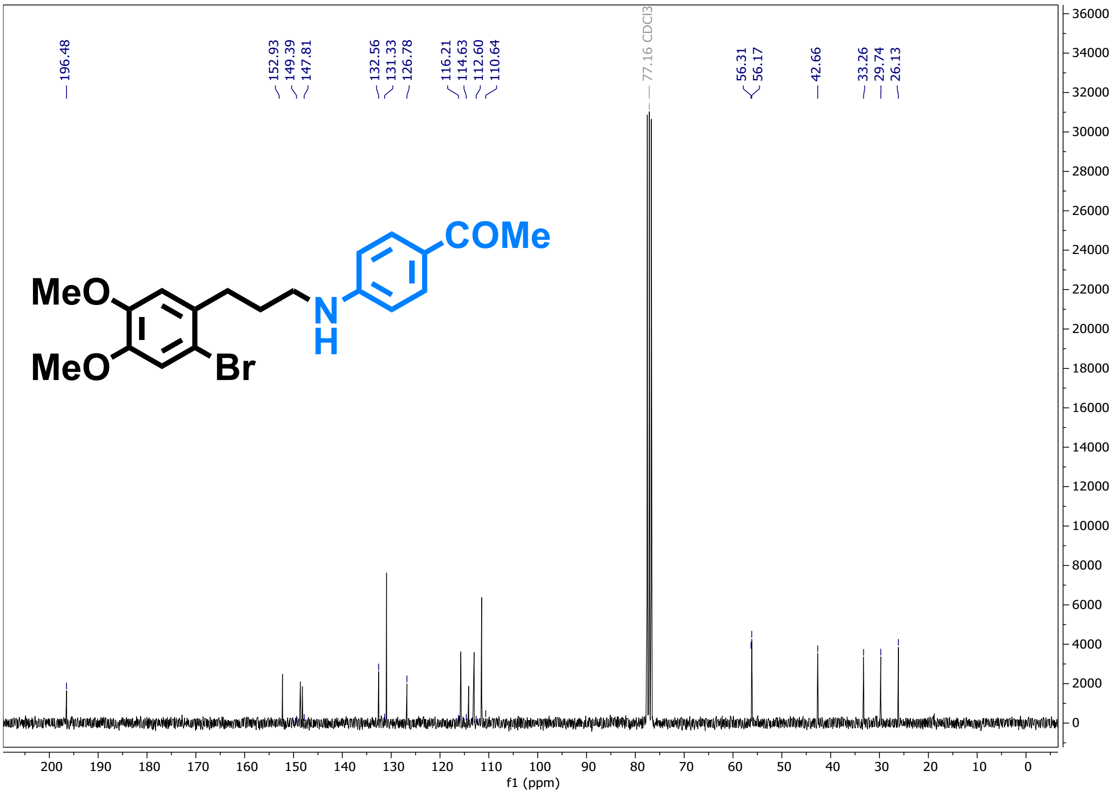

Fig. S9 ^{1}H and ^{13}C NMR spectra of compound **1Gc3**.

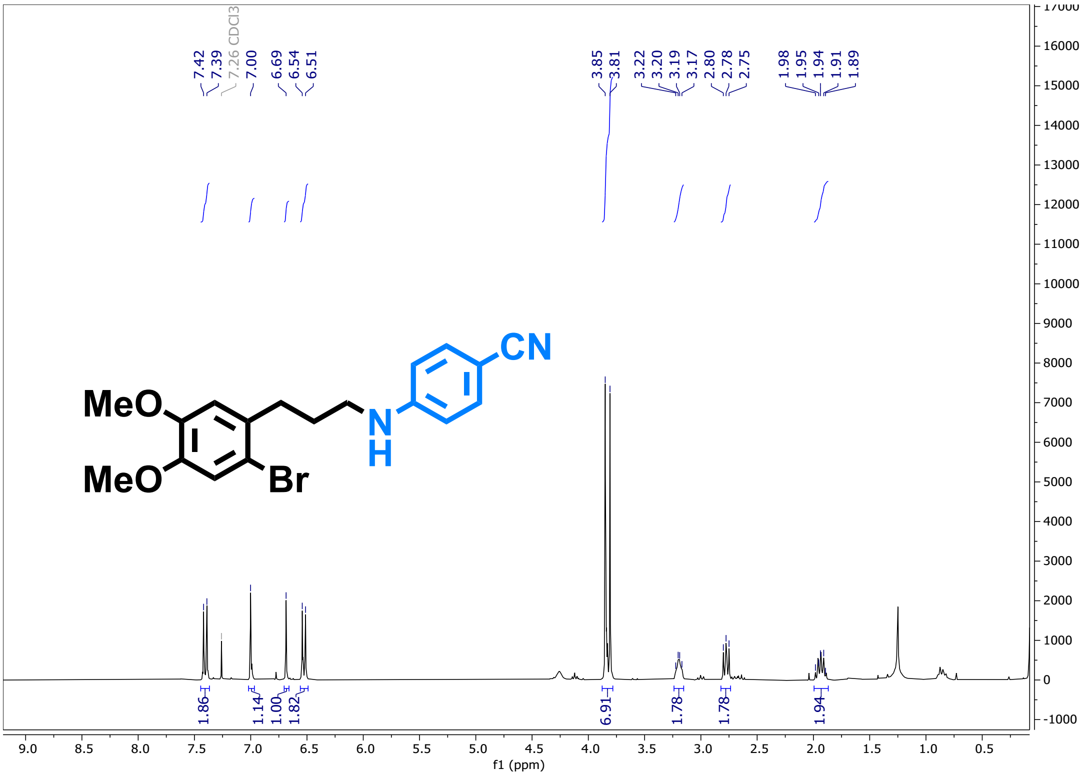

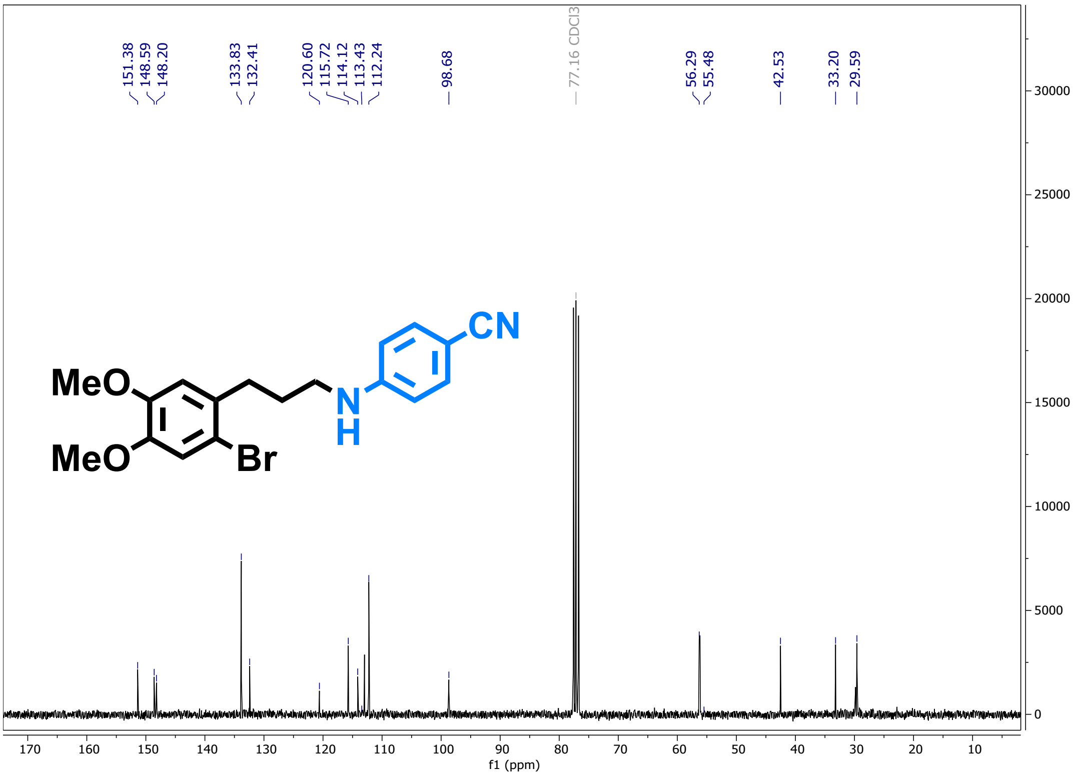

Fig. S10 ^{1}H and ^{13}C NMR spectra of compound **1Gc4**.

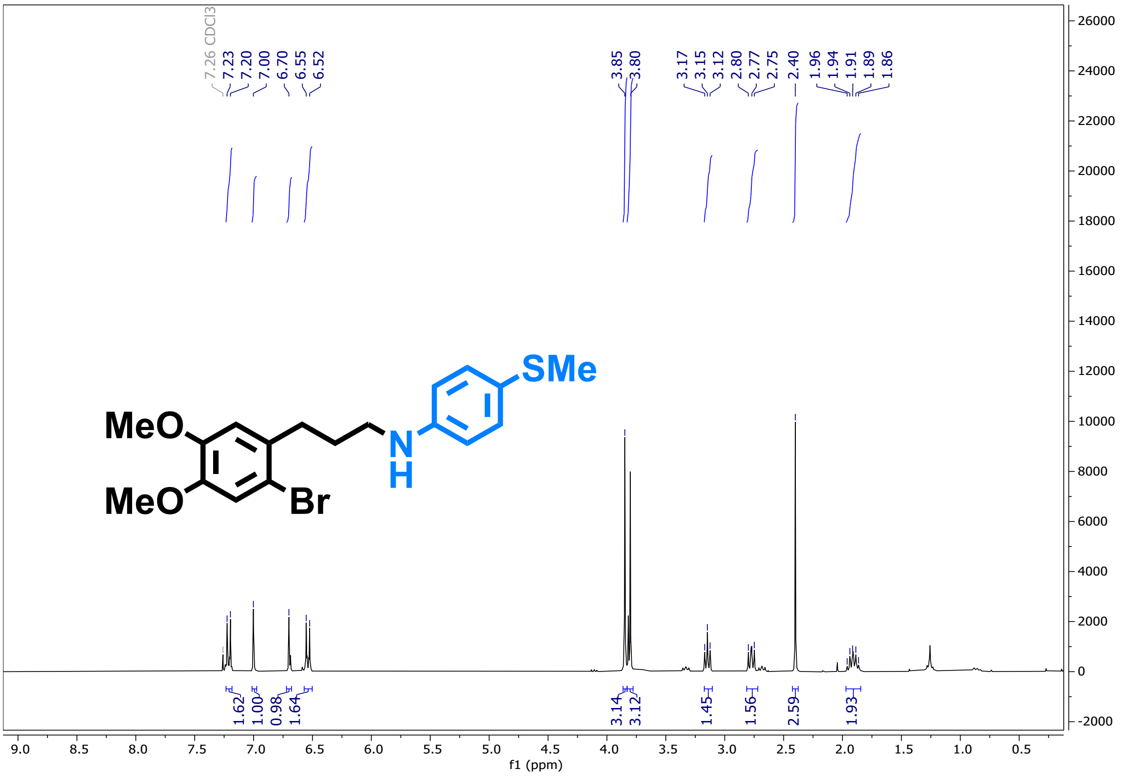

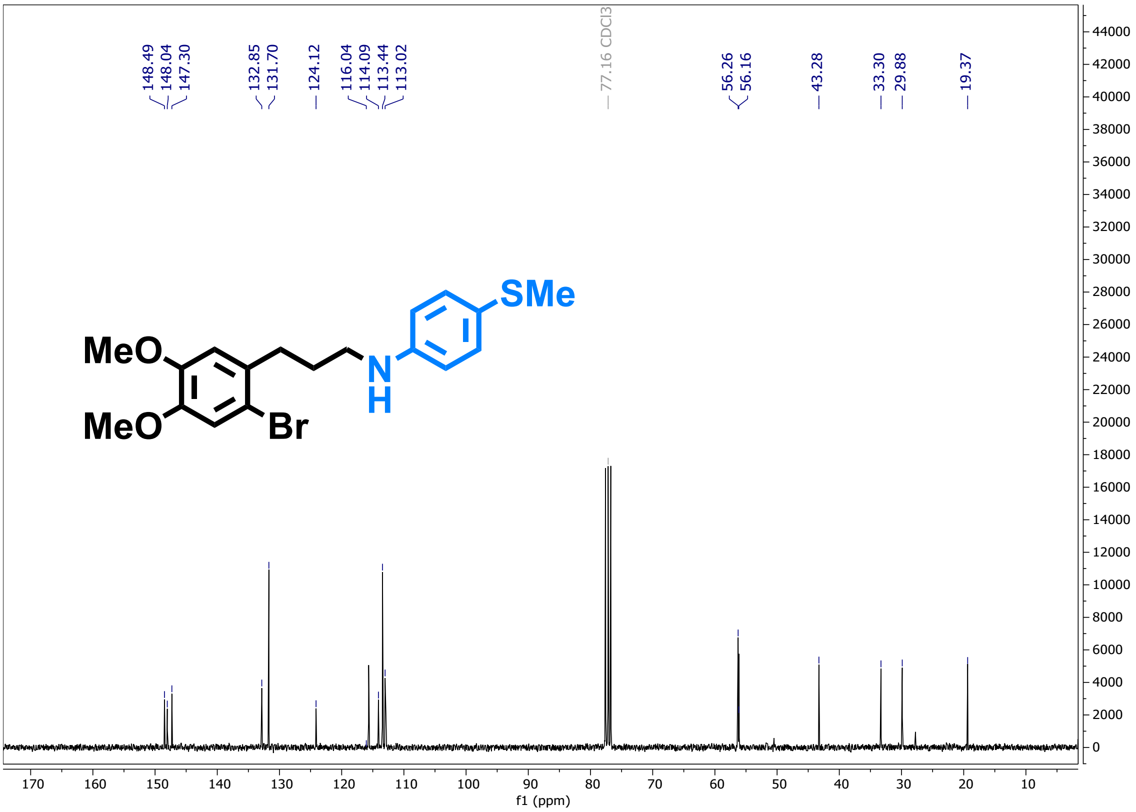

Fig. S11 ^{1}H and ^{13}C NMR spectra of compound **1Gc5**.

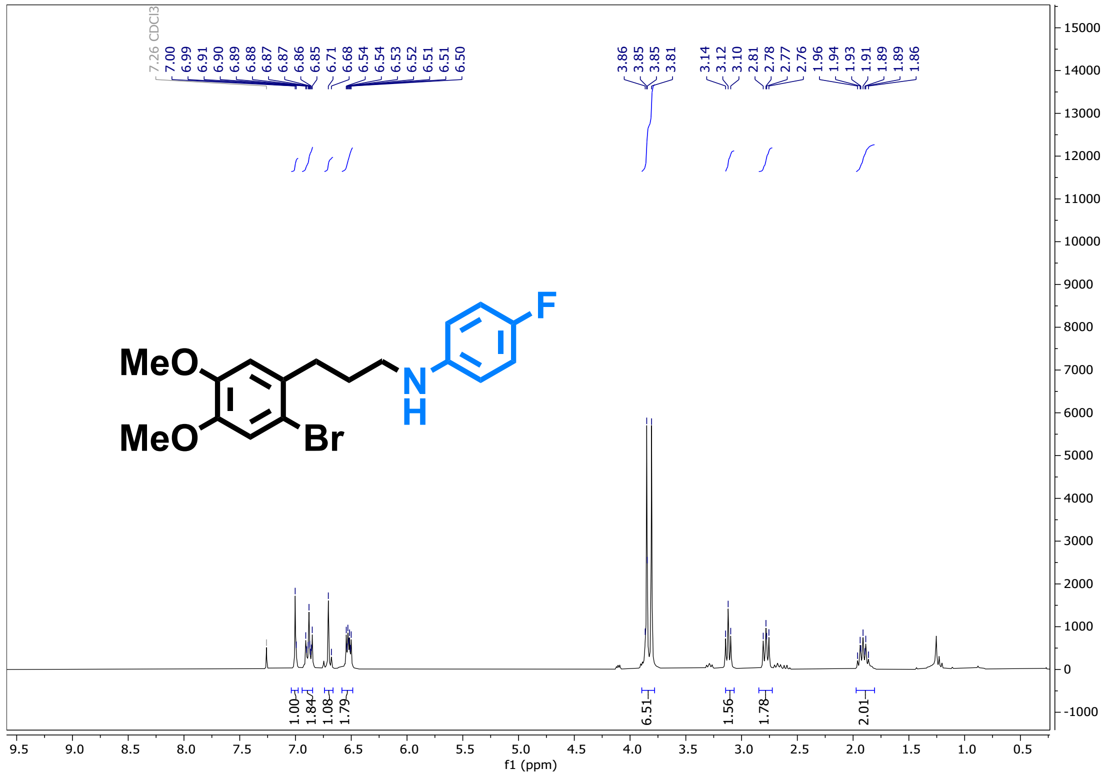

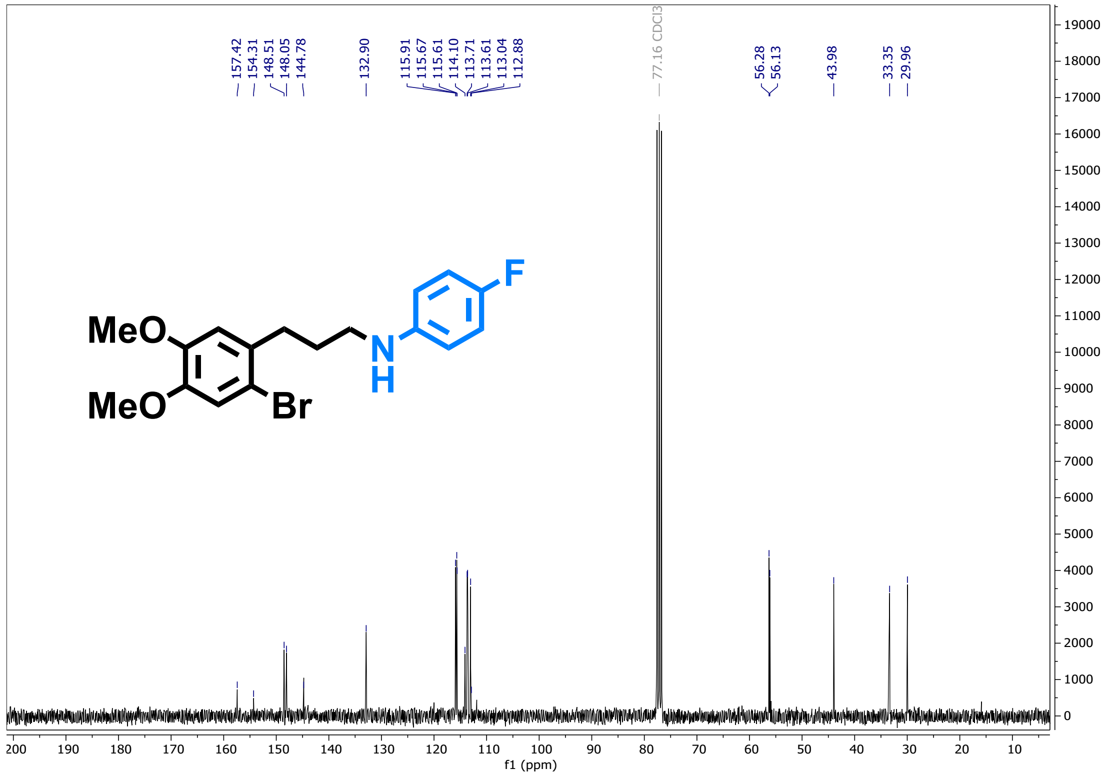

Fig. S12 ^{1}H and ^{13}C NMR spectra of compound **1Gc6**.

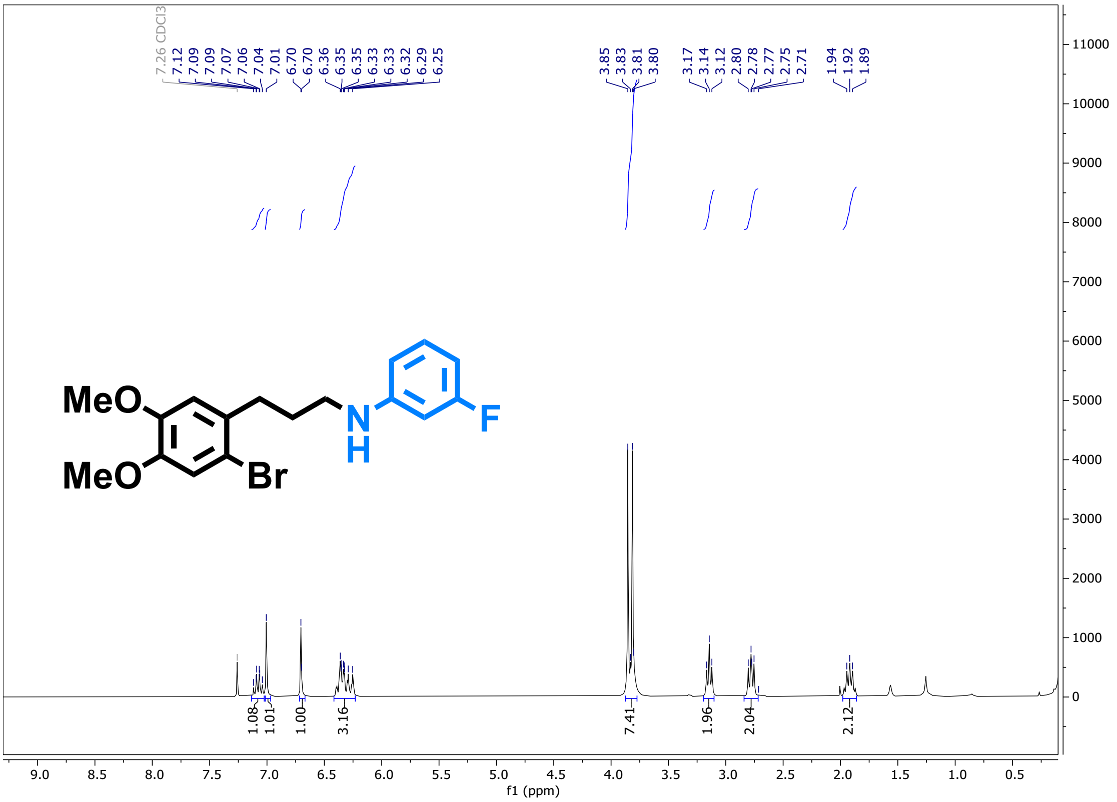

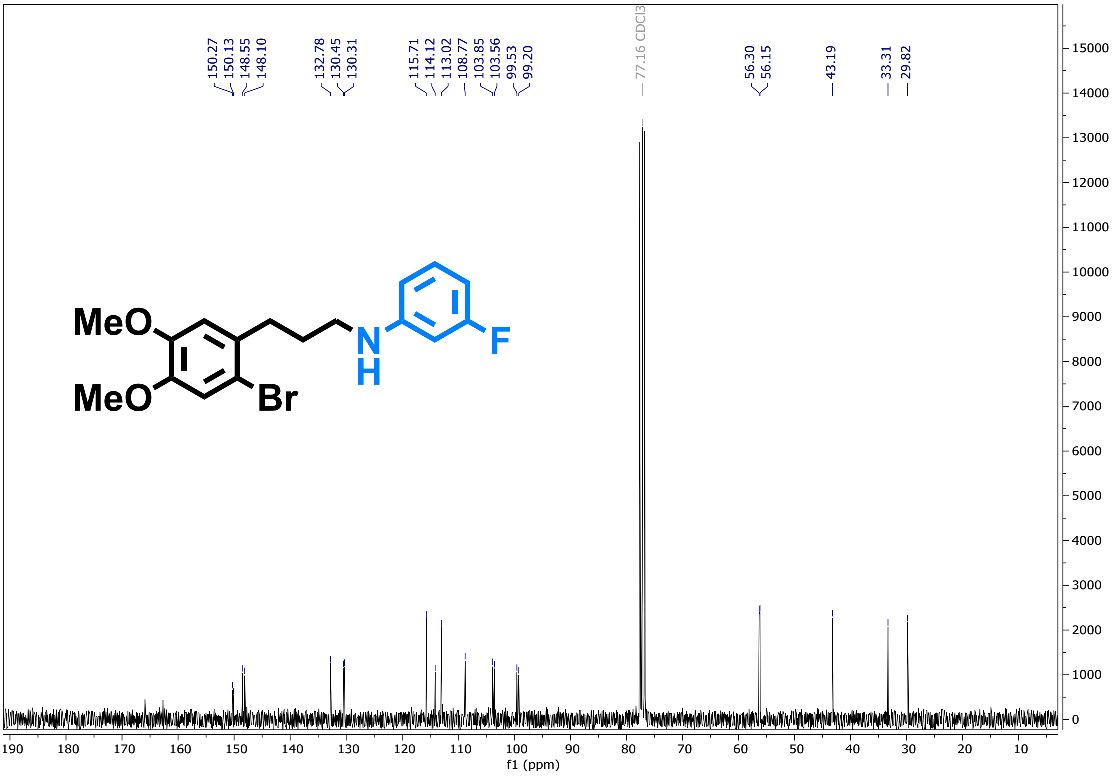

Fig. S13 ^{1}H and ^{13}C NMR spectra of compound **1Gc7**.

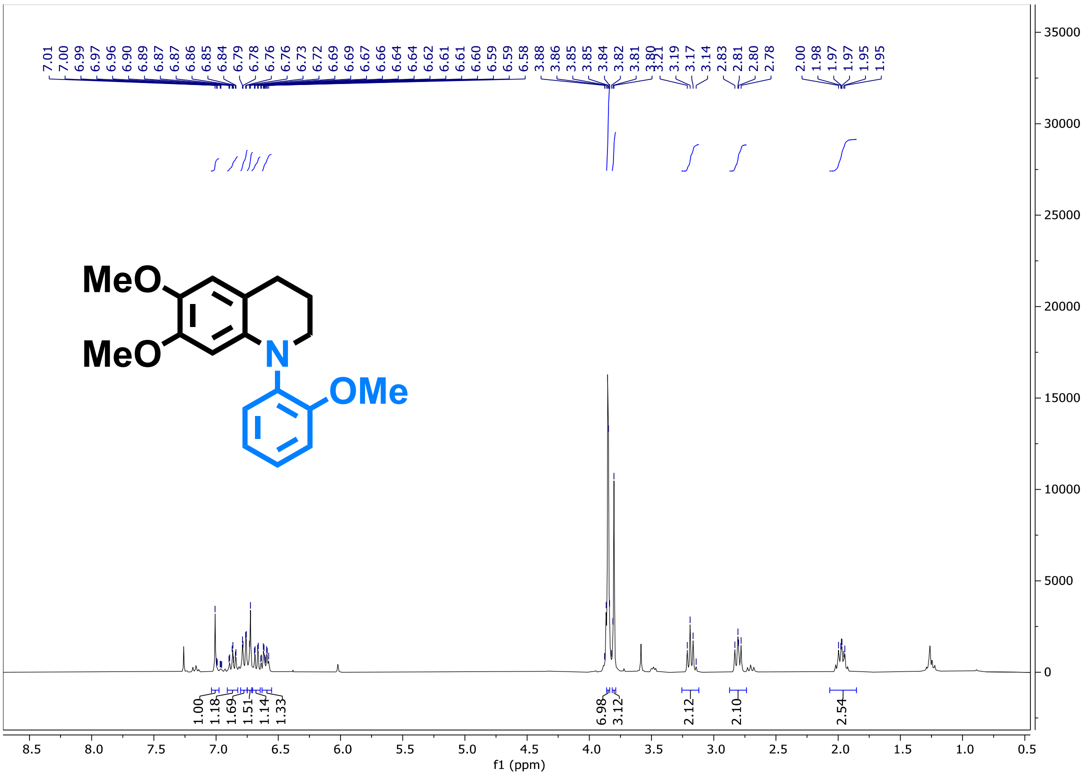

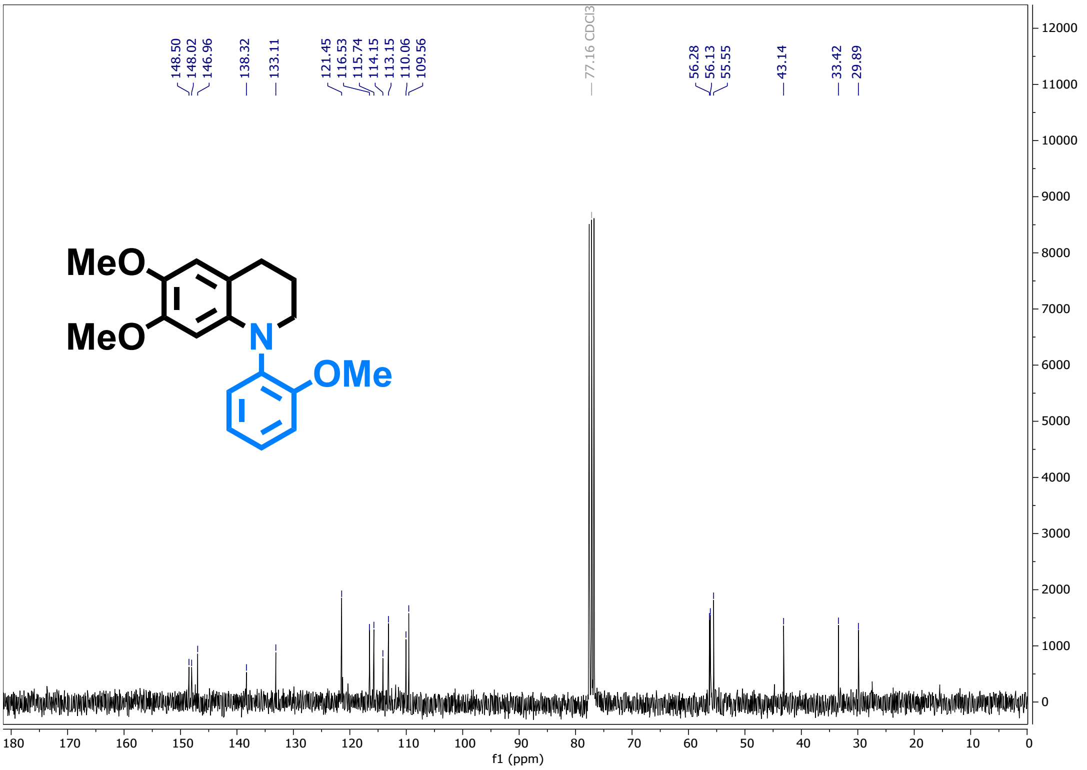

Fig. S14 ^{1}H and ^{13}C NMR spectra of compound **1Gd1**.

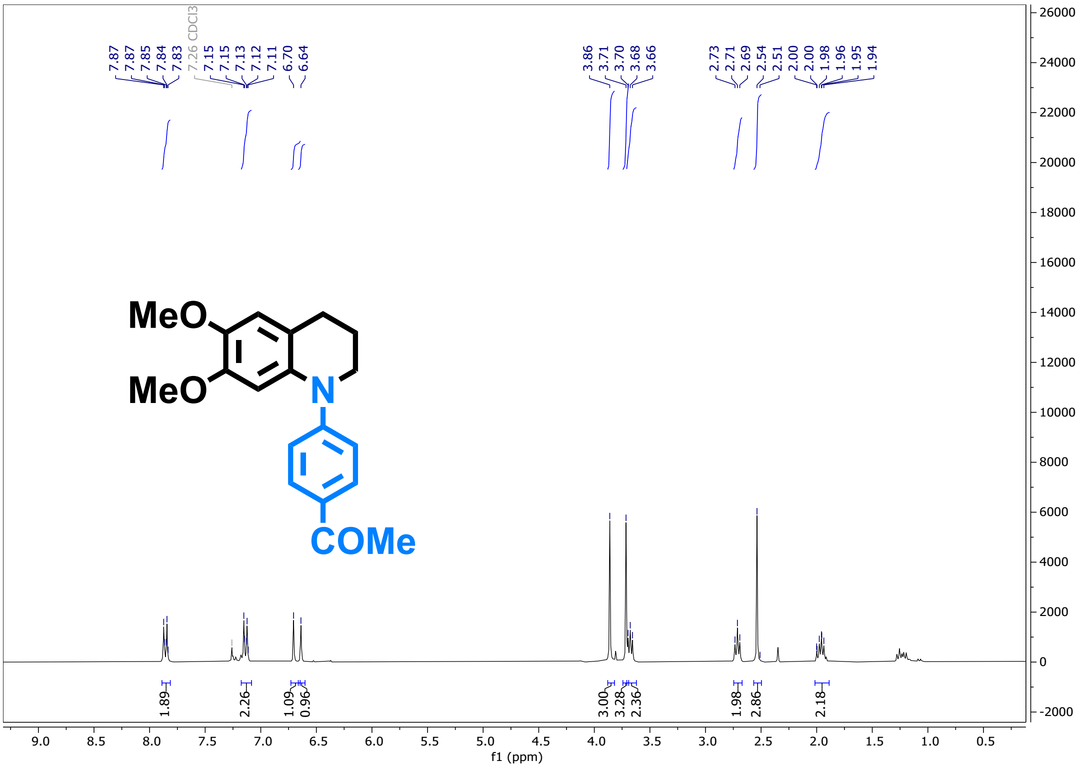

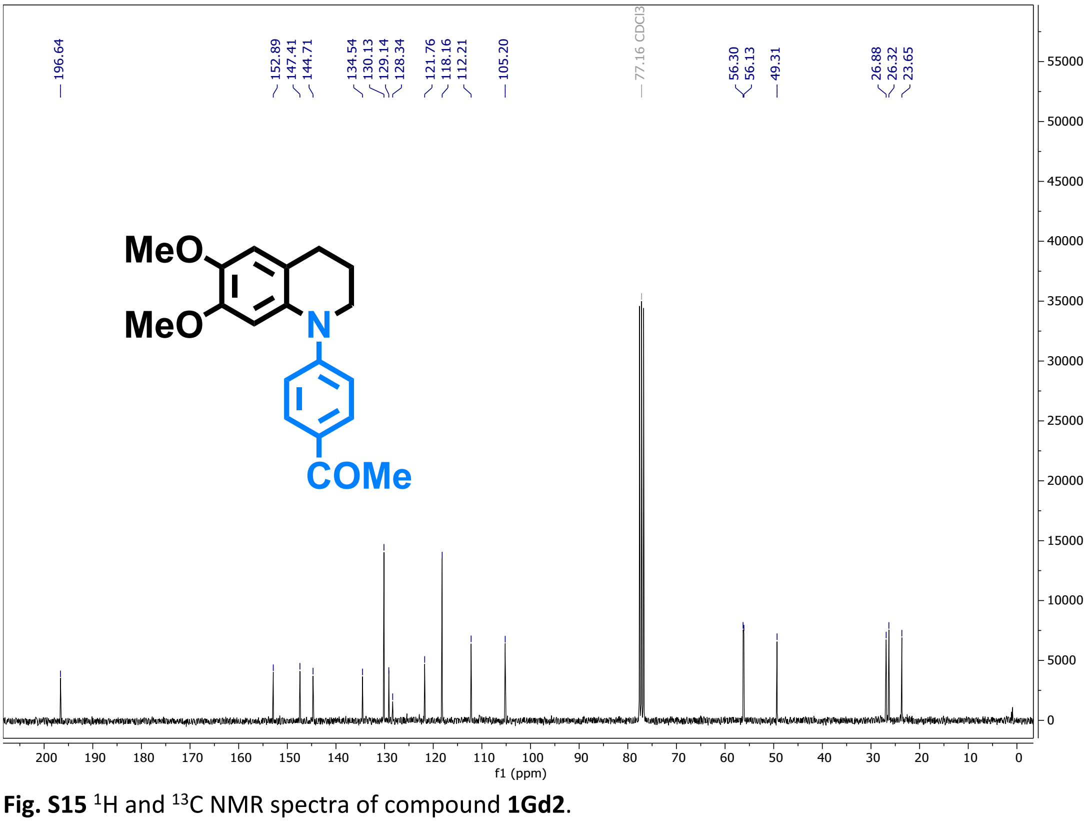

Fig. S15 ^{1}H and ^{13}C NMR spectra of compound **1Gd2**.

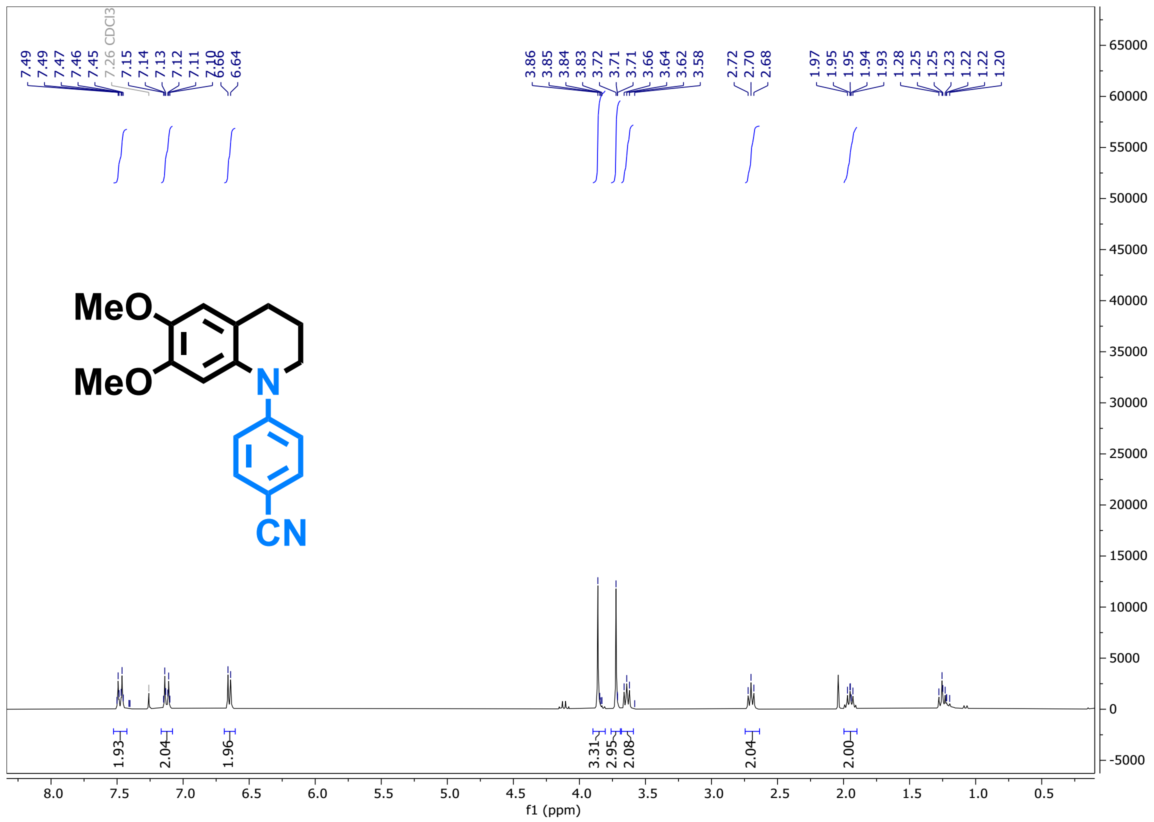

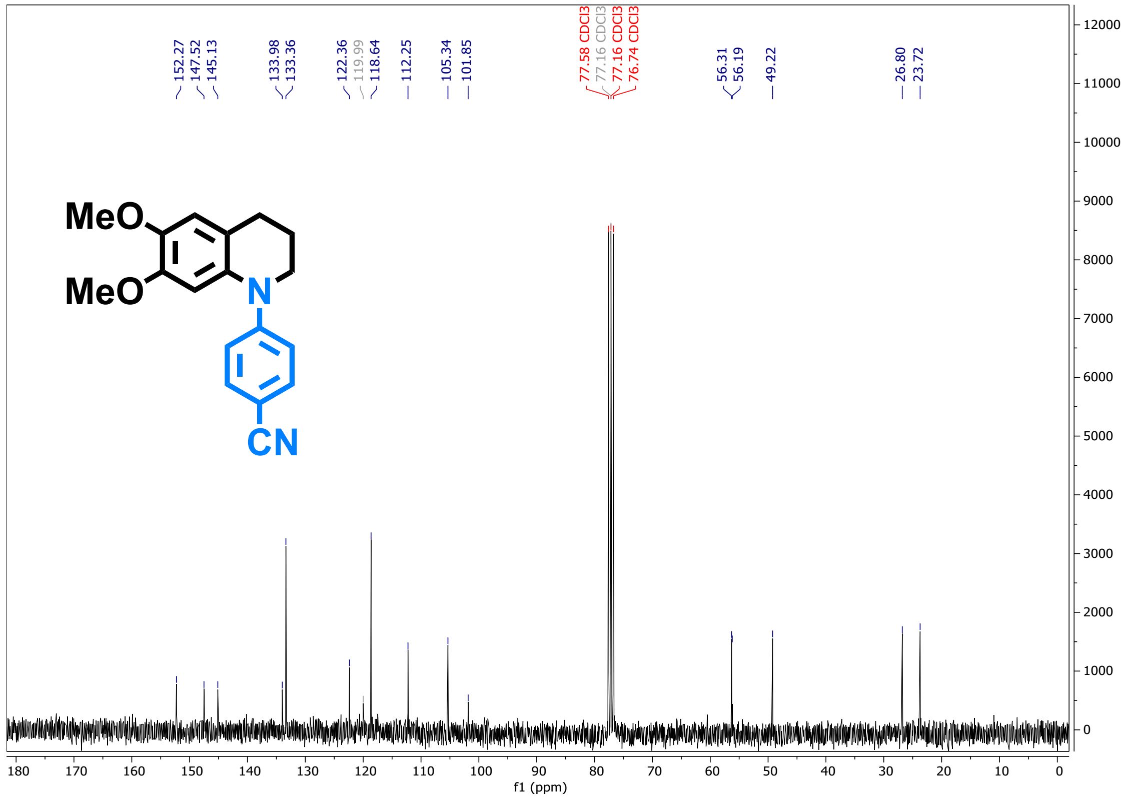

Fig. S16 ^{1}H and ^{13}C NMR spectra of compound **1Gd3**.

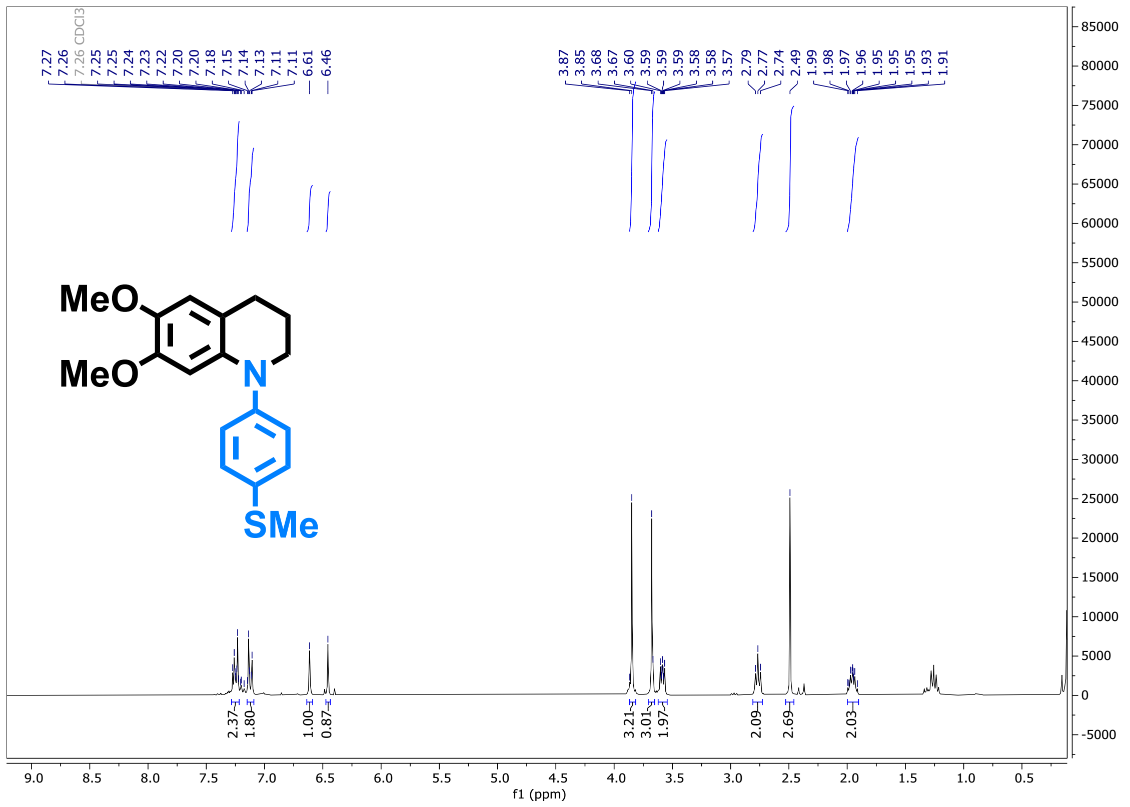

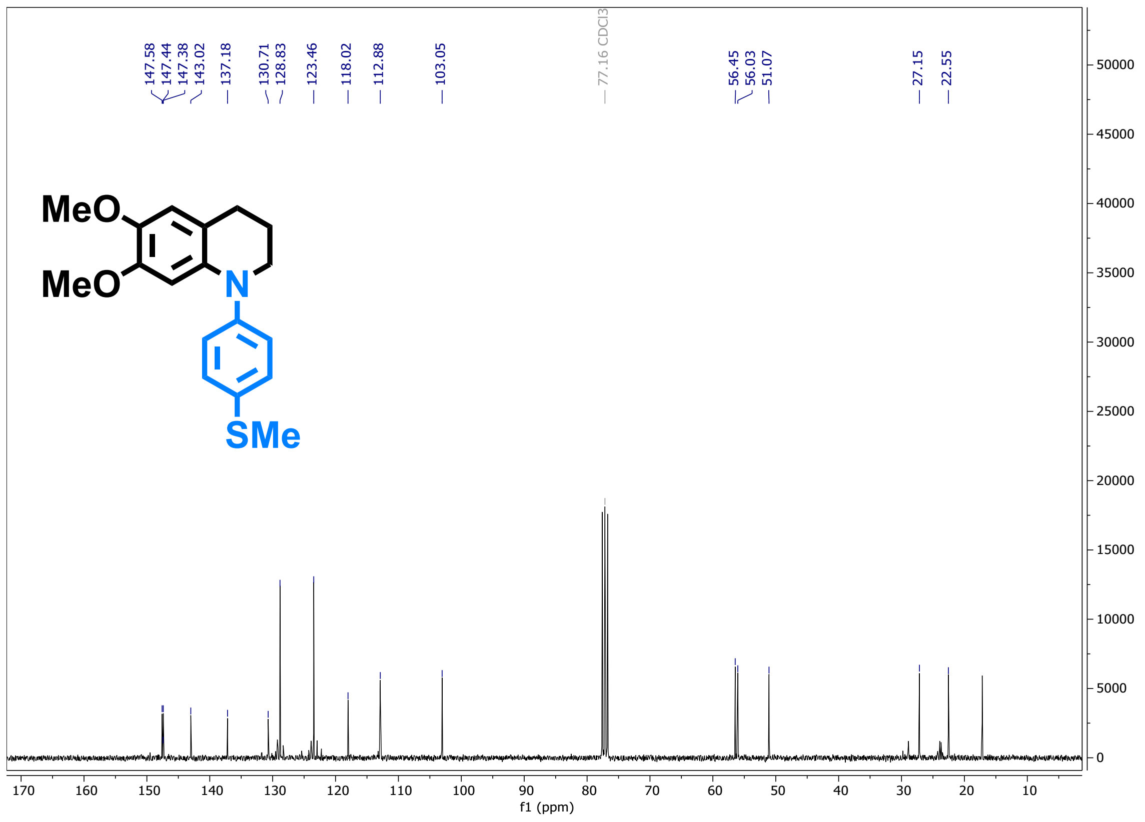

Fig. S17 ^{1}H and ^{13}C NMR spectra of compound **1Gd5**.

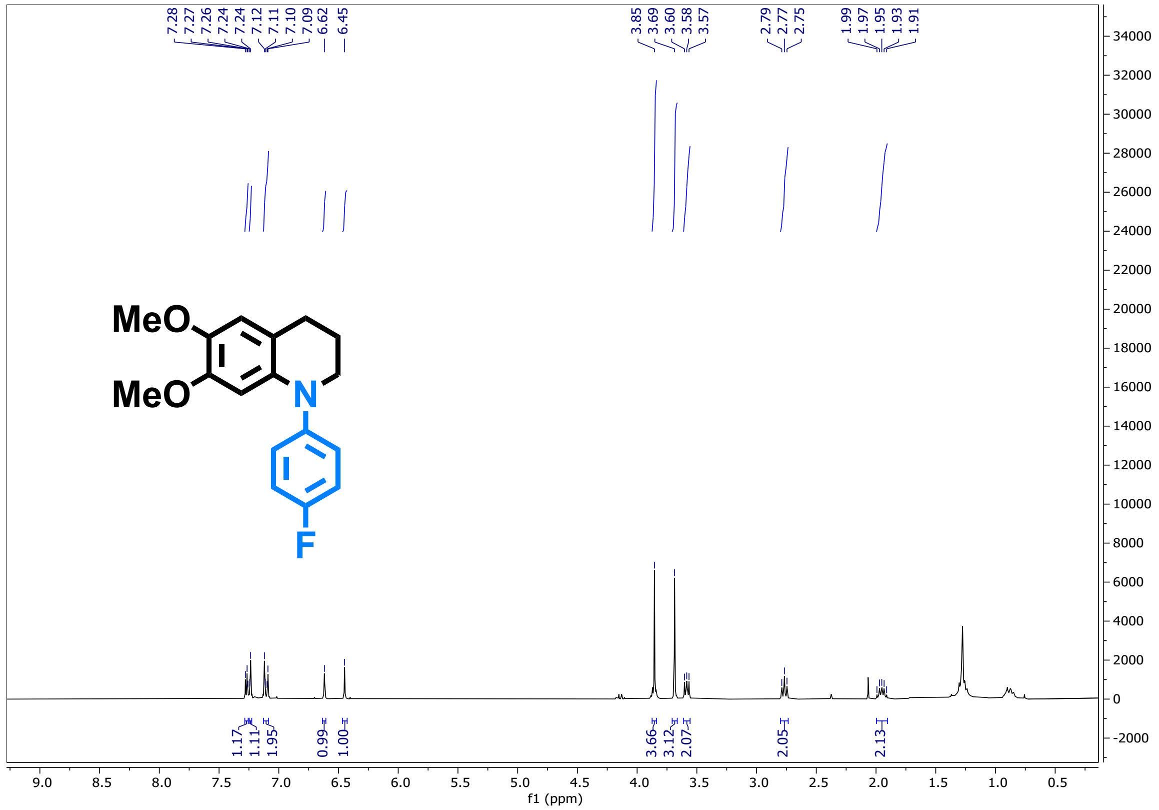

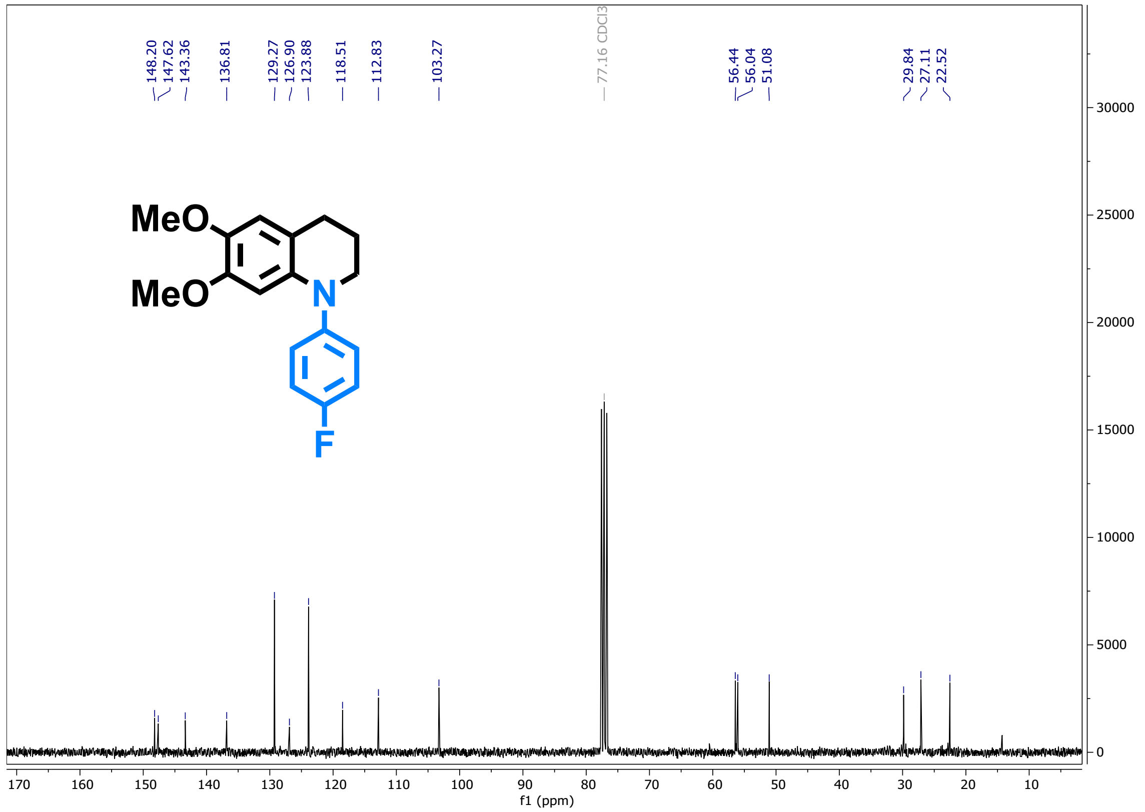

Fig. S18 ^{1}H and ^{13}C NMR spectra of compound **1Gd6**.

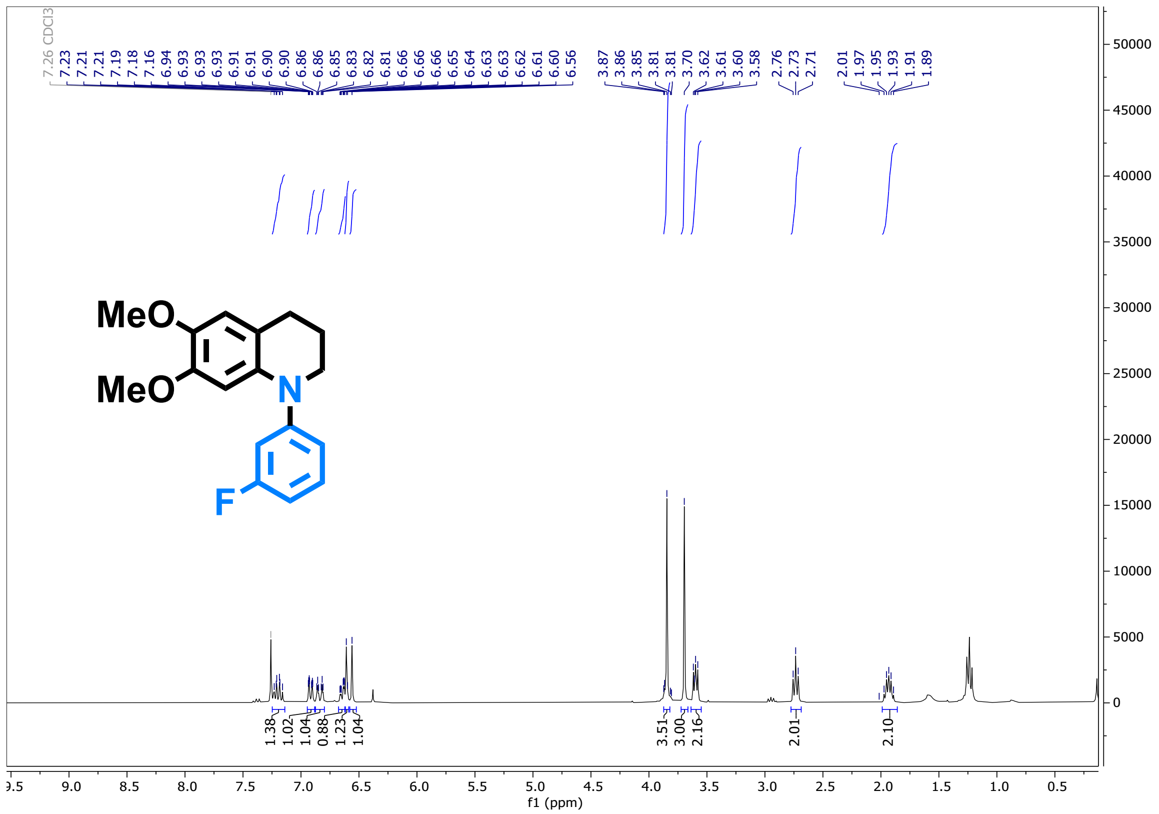

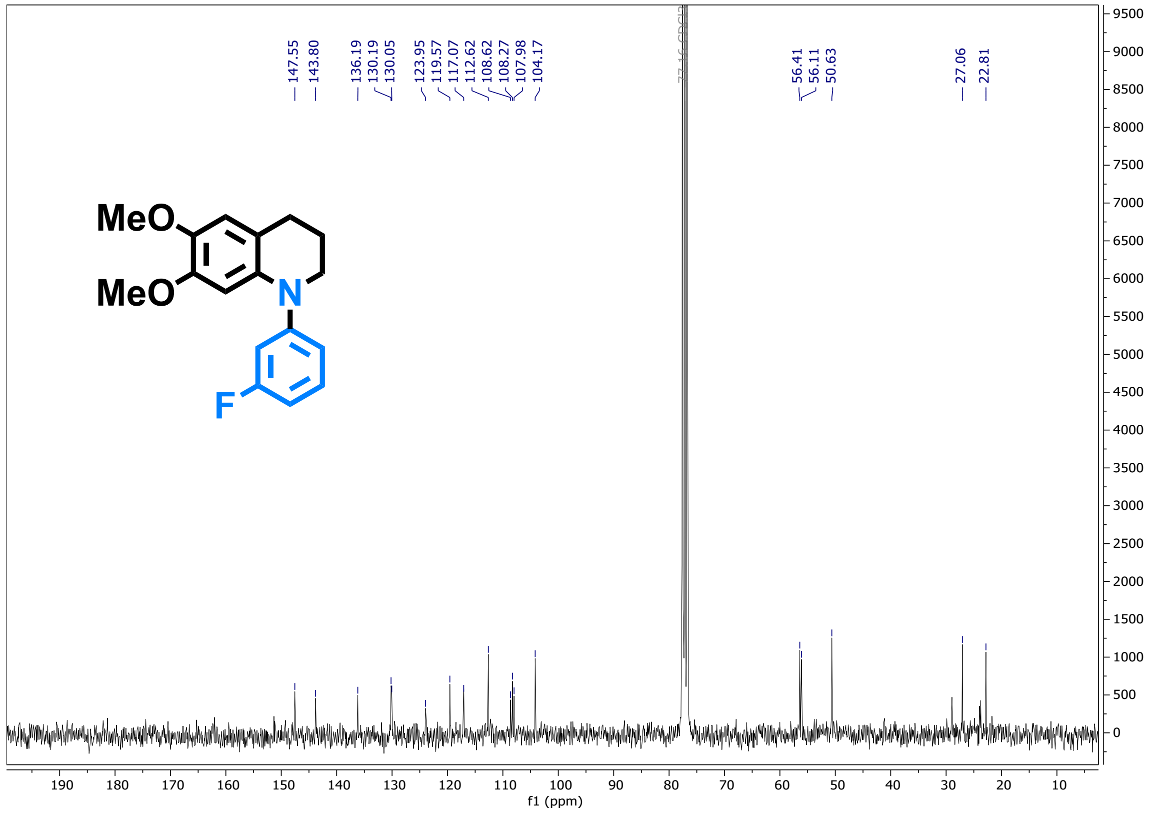

Fig. S19 ^{1}H and ^{13}C NMR spectra of compound **1Gd7**.

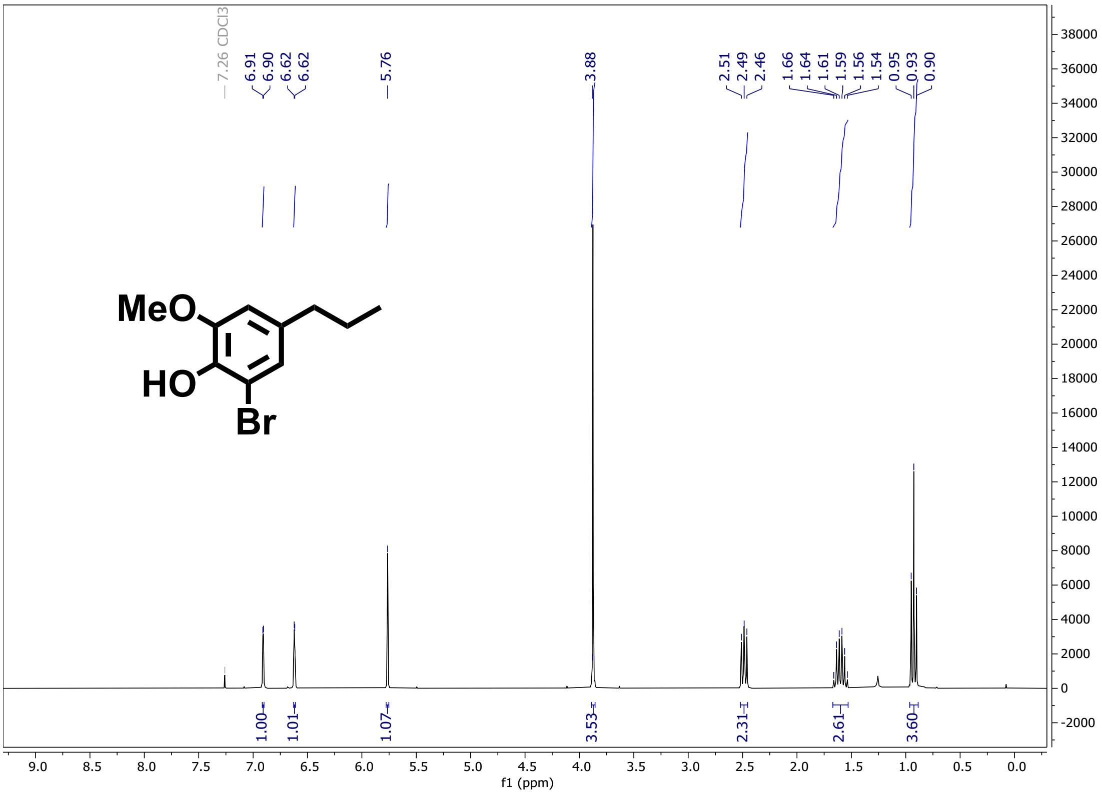

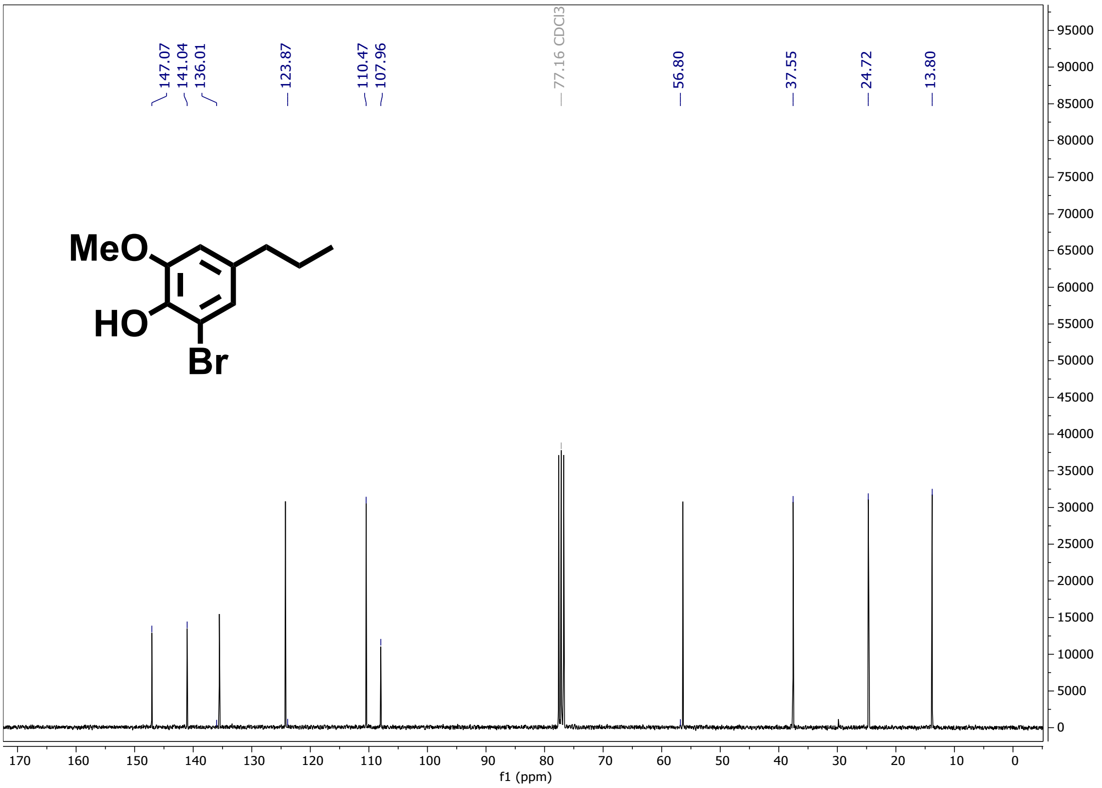

Fig. S20 ^{1}H and ^{13}C NMR spectra of compound **2Ga**.

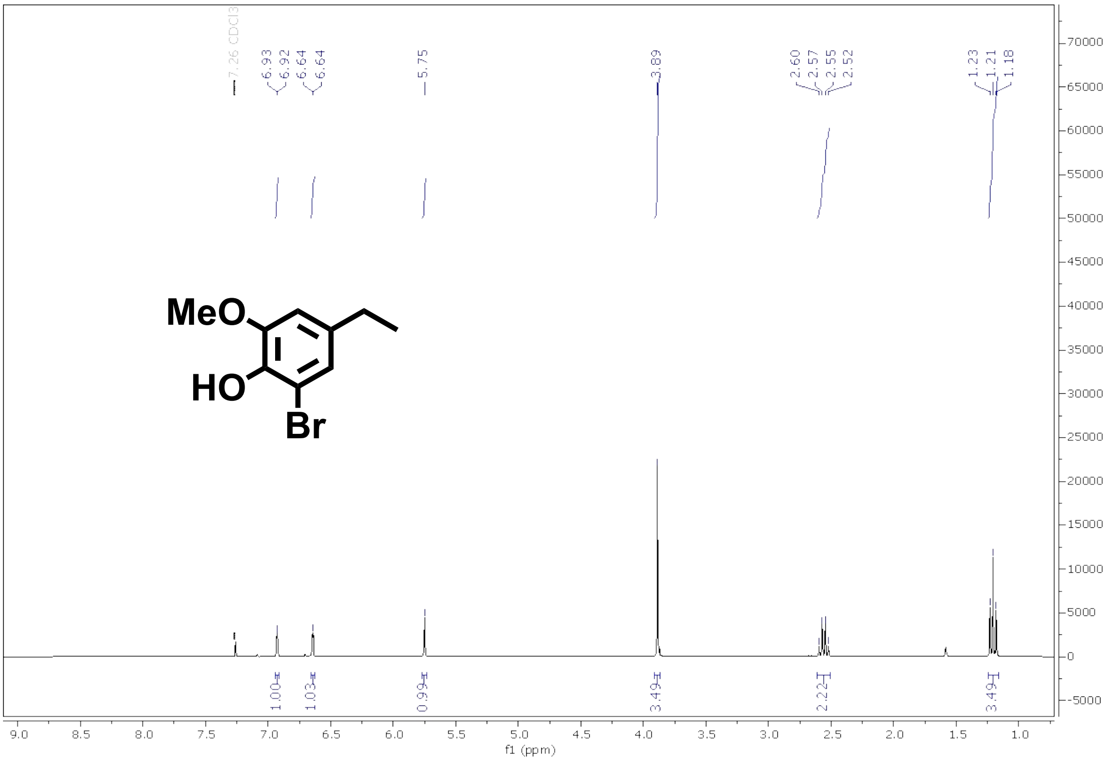

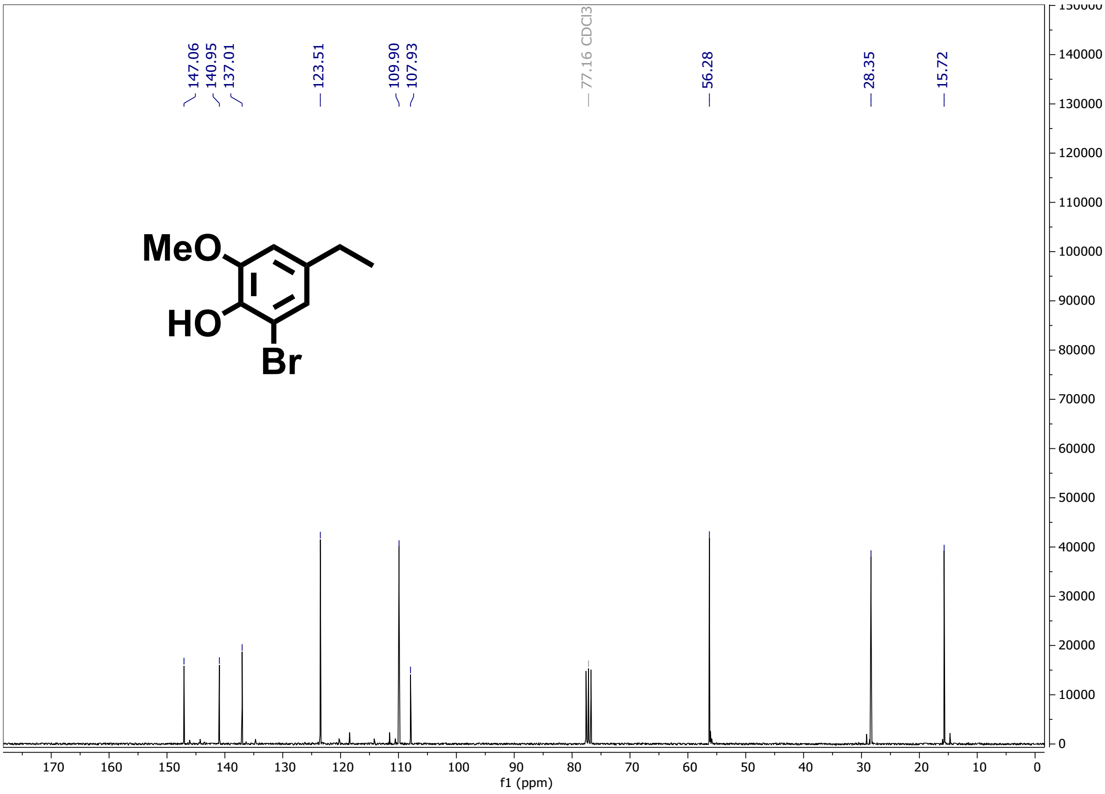

Fig. S21 ^{1}H and ^{13}C NMR spectra of compound **3Ga**.

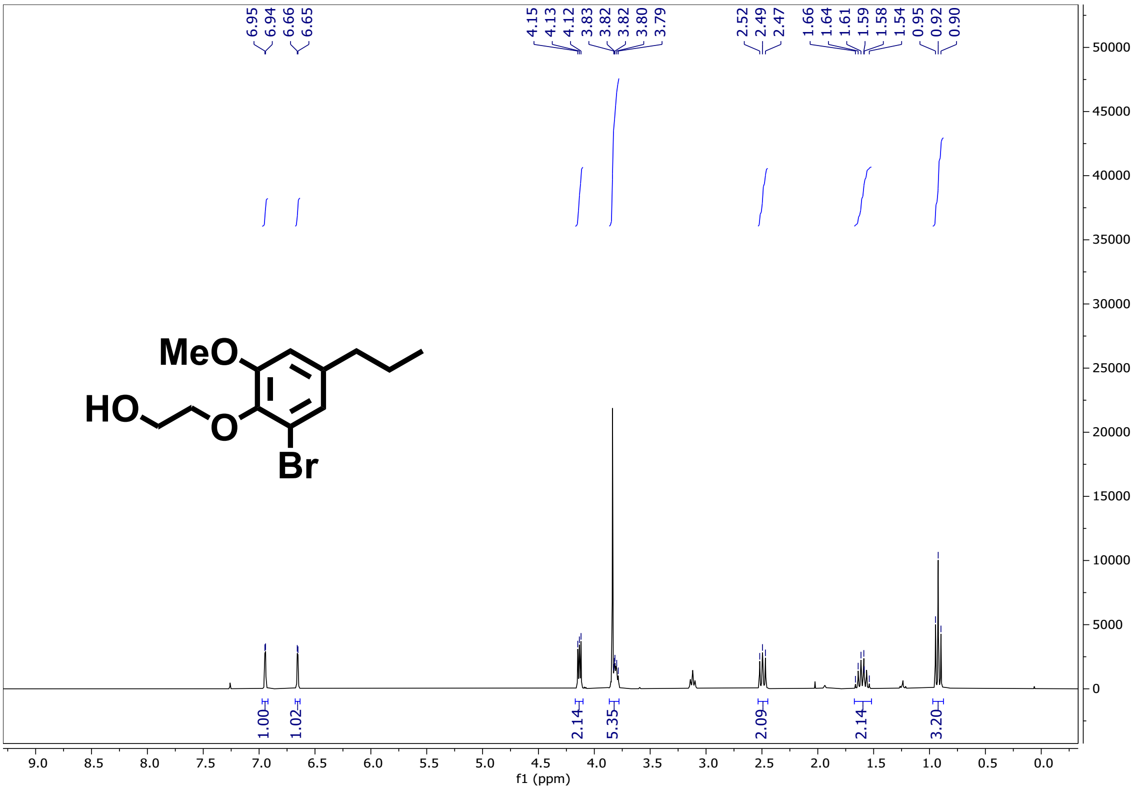

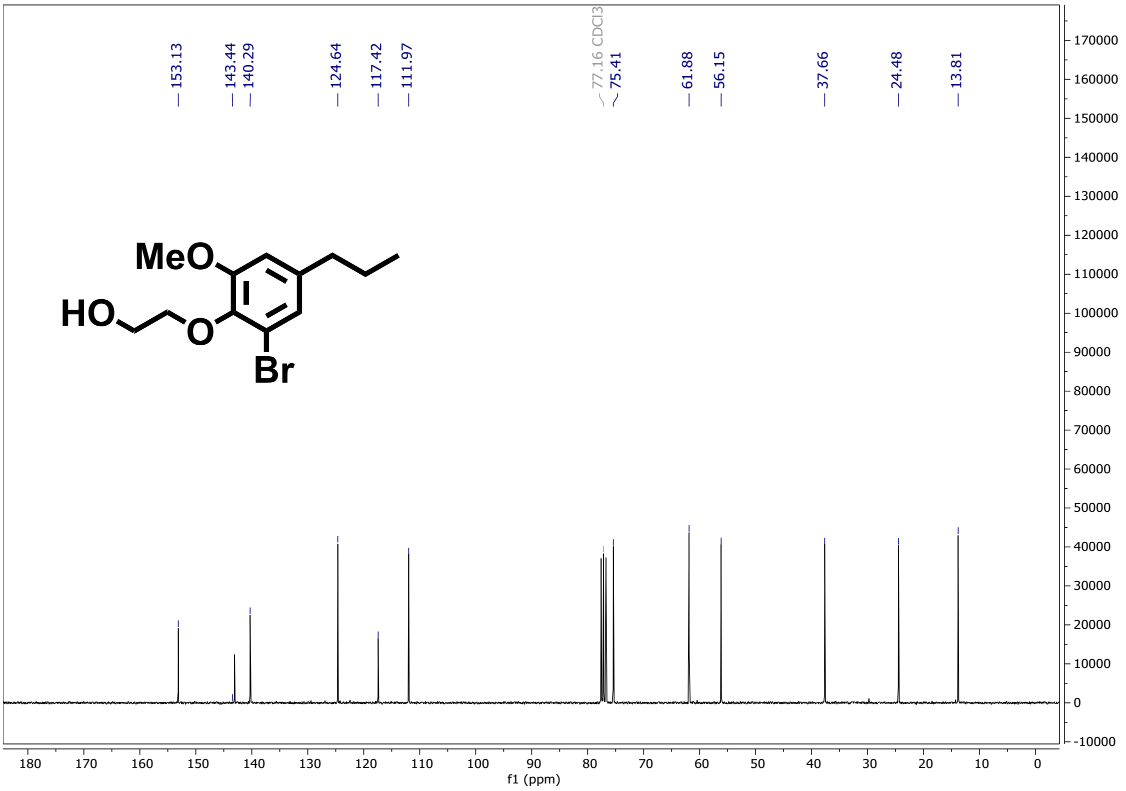

Fig. S22 ^{1}H and ^{13}C NMR spectra of compound **2Gb**.

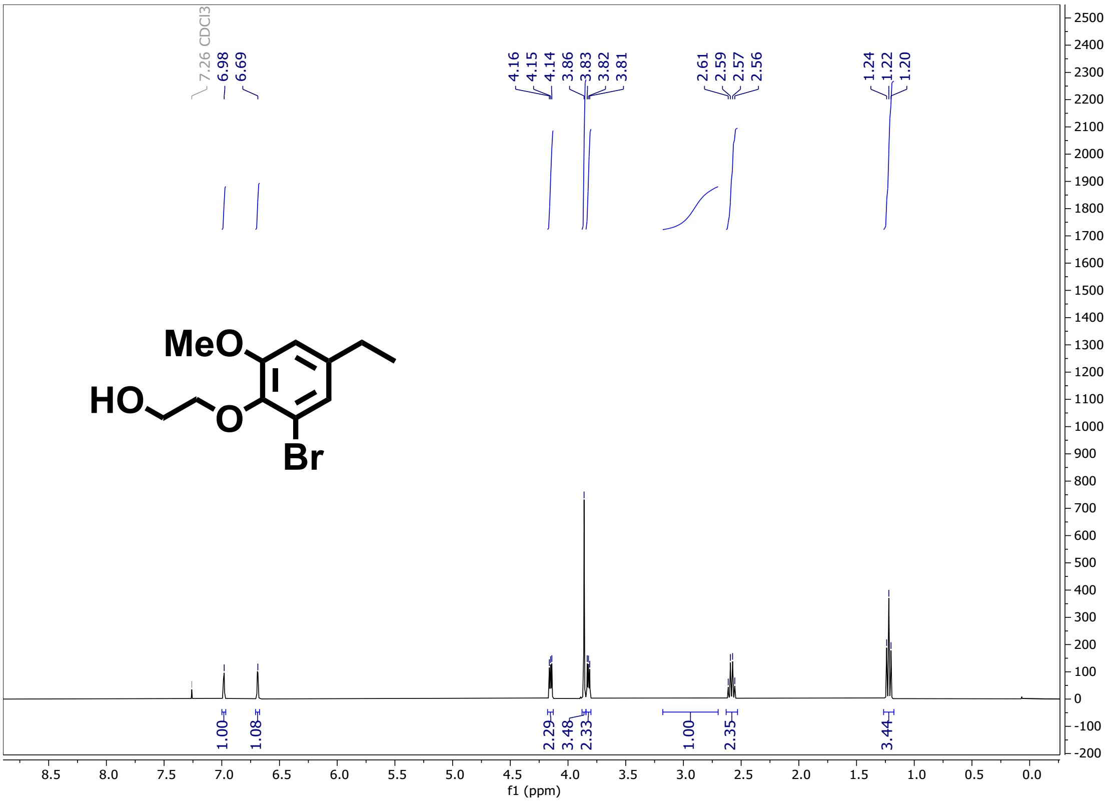

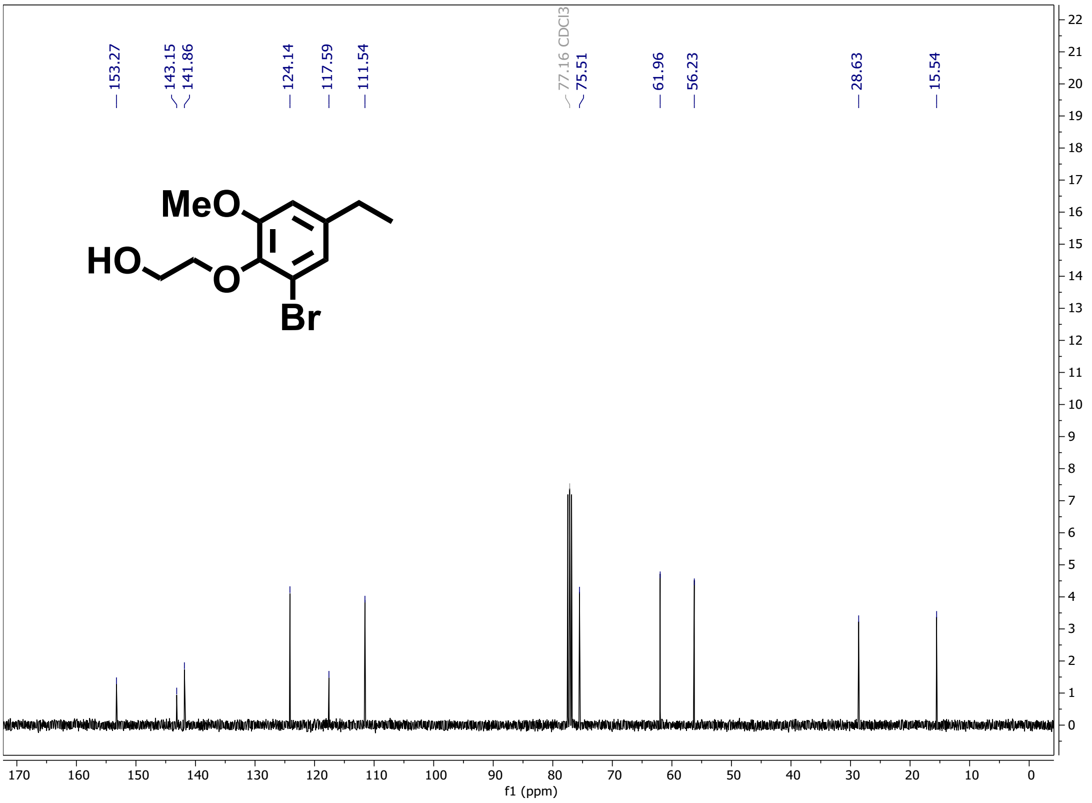

Fig. S23 ^{1}H and ^{13}C NMR spectra of compound **3Gb**.

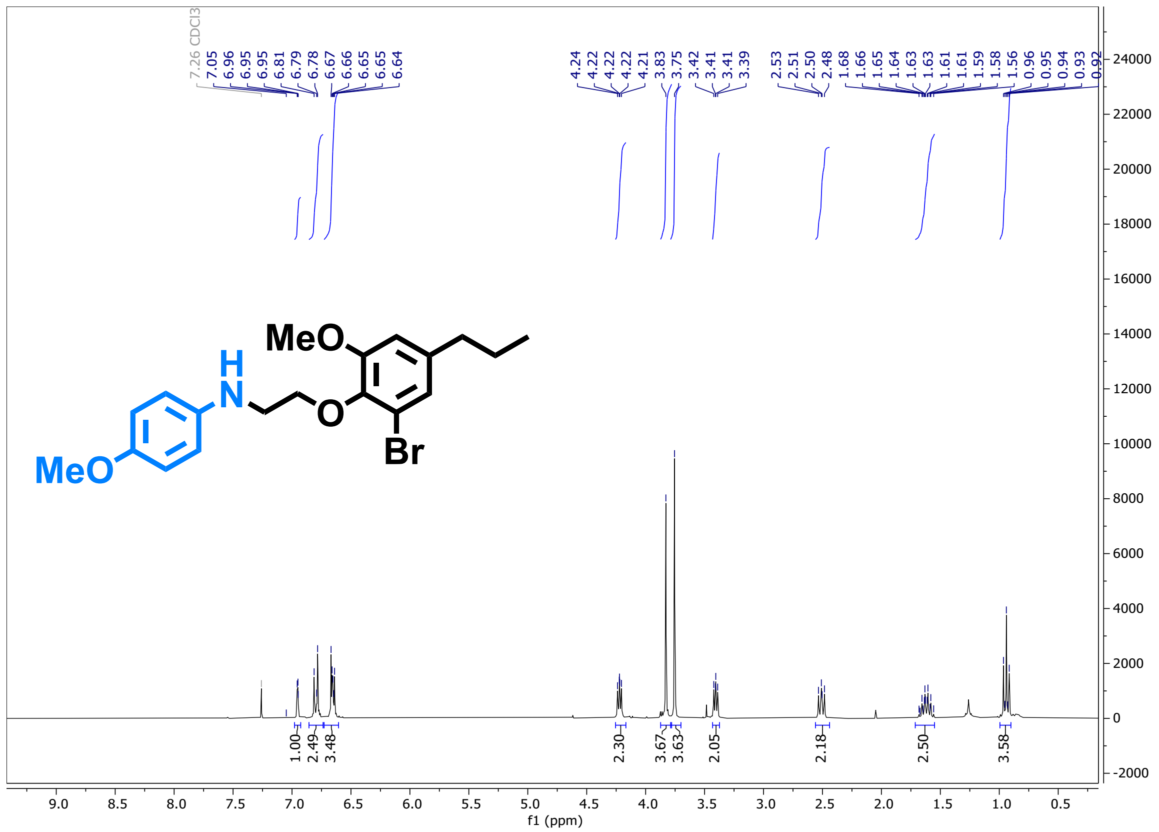

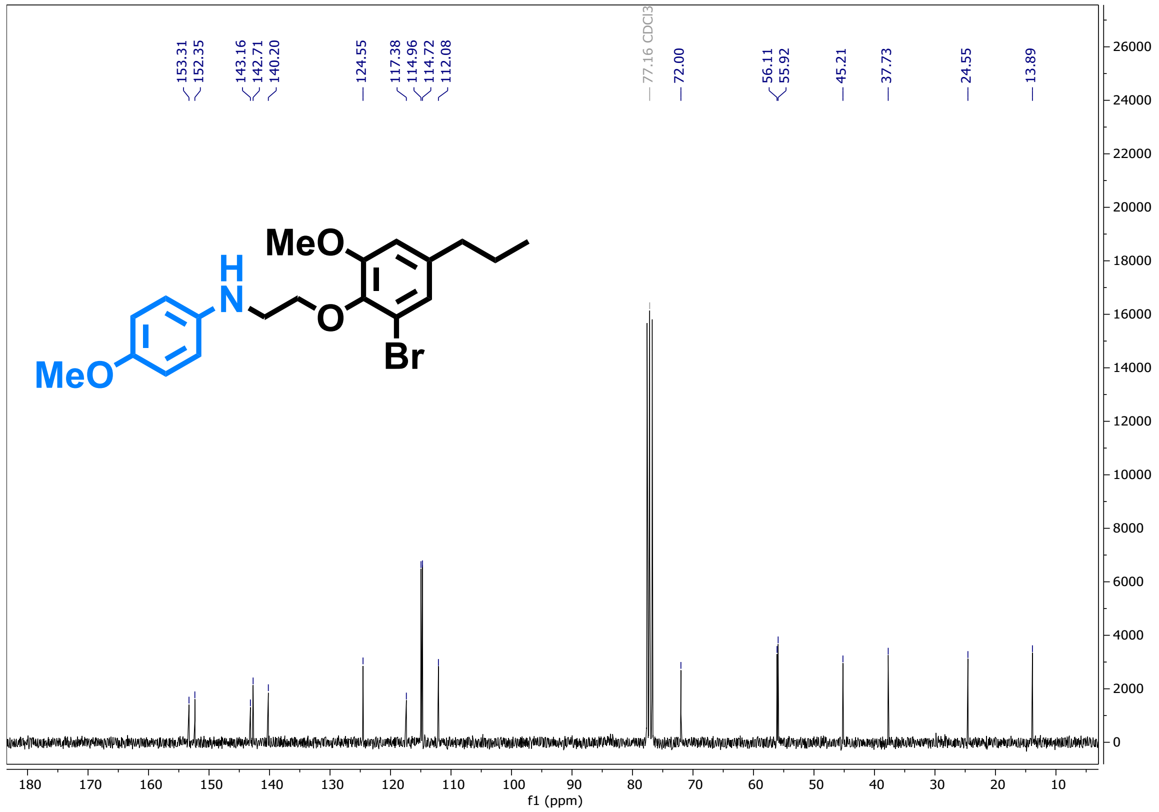

Fig. S24 ^{1}H and ^{13}C NMR spectra of compound **2Gc1**.

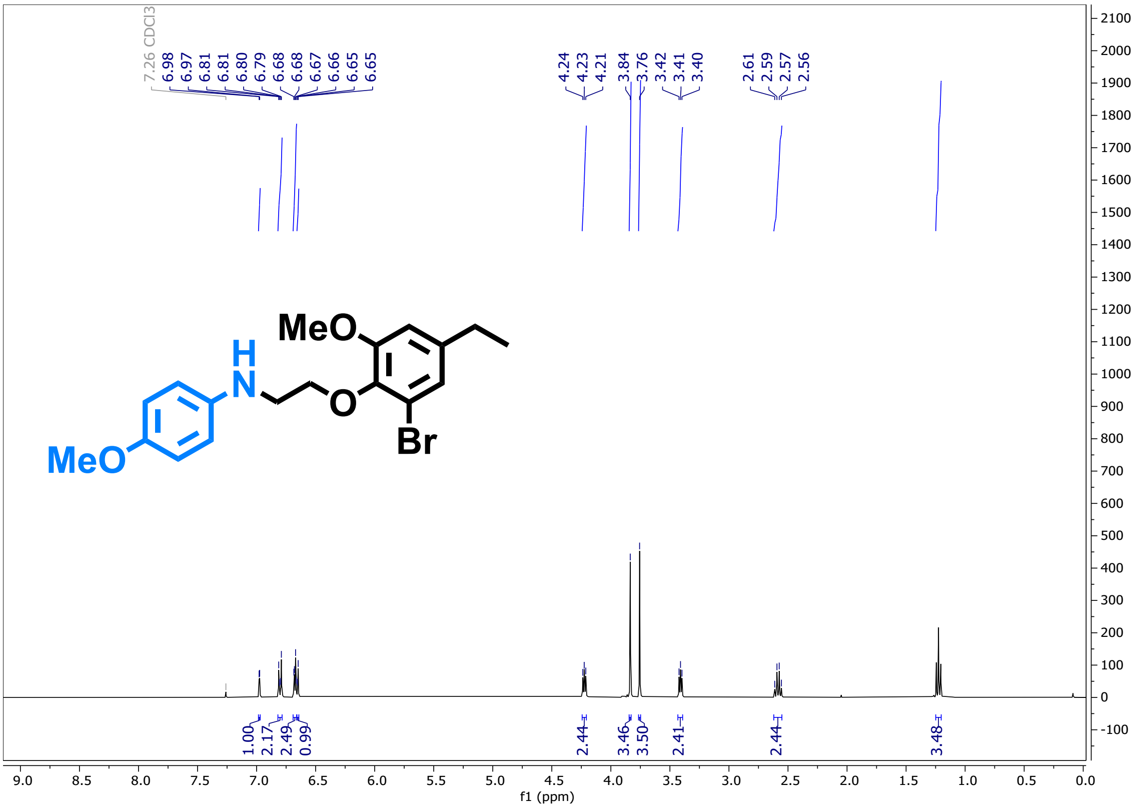

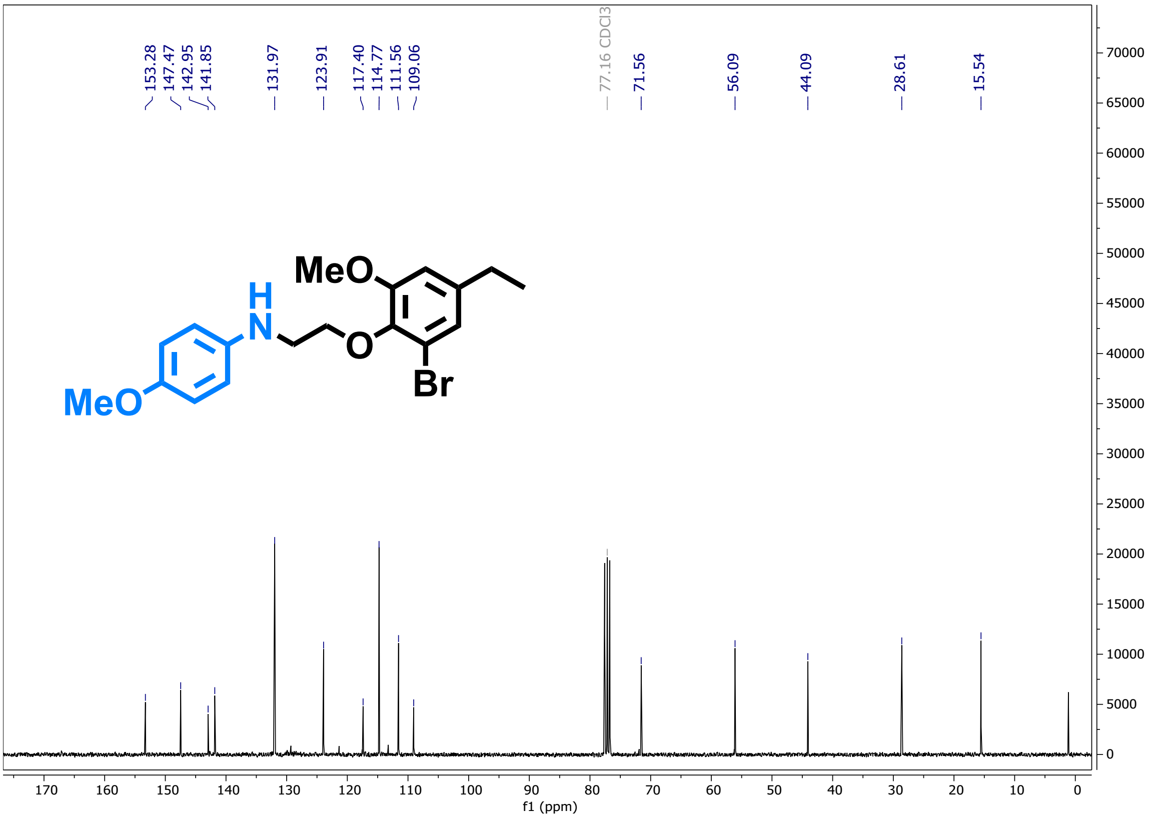

Fig. S25 ^{1}H and ^{13}C NMR spectra of compound **3Gc1**.

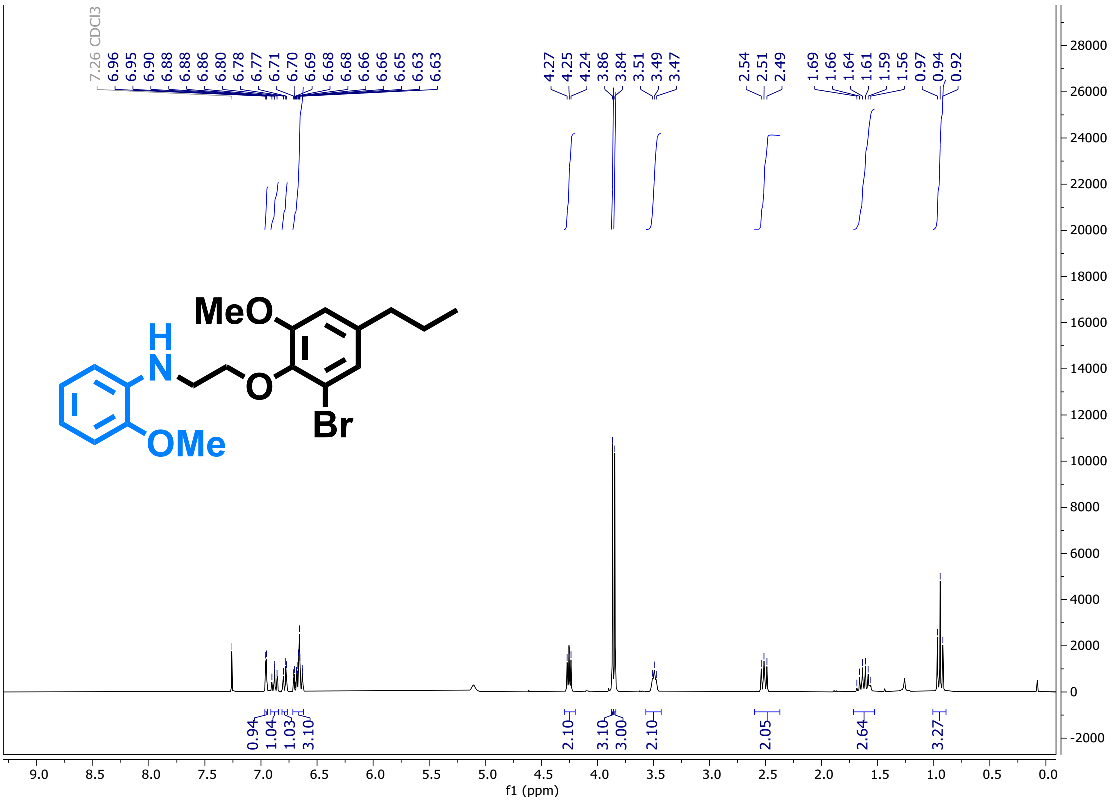

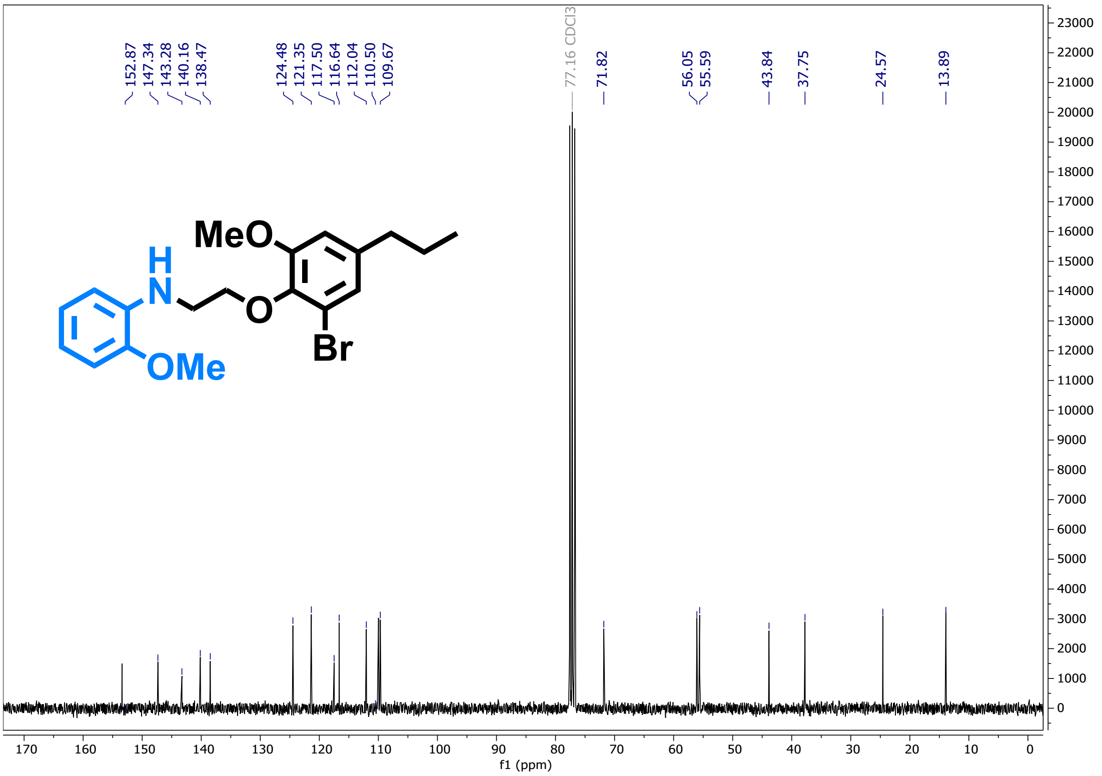

Fig. S26 ^{1}H and ^{13}C NMR spectra of compound **2Gc2**.

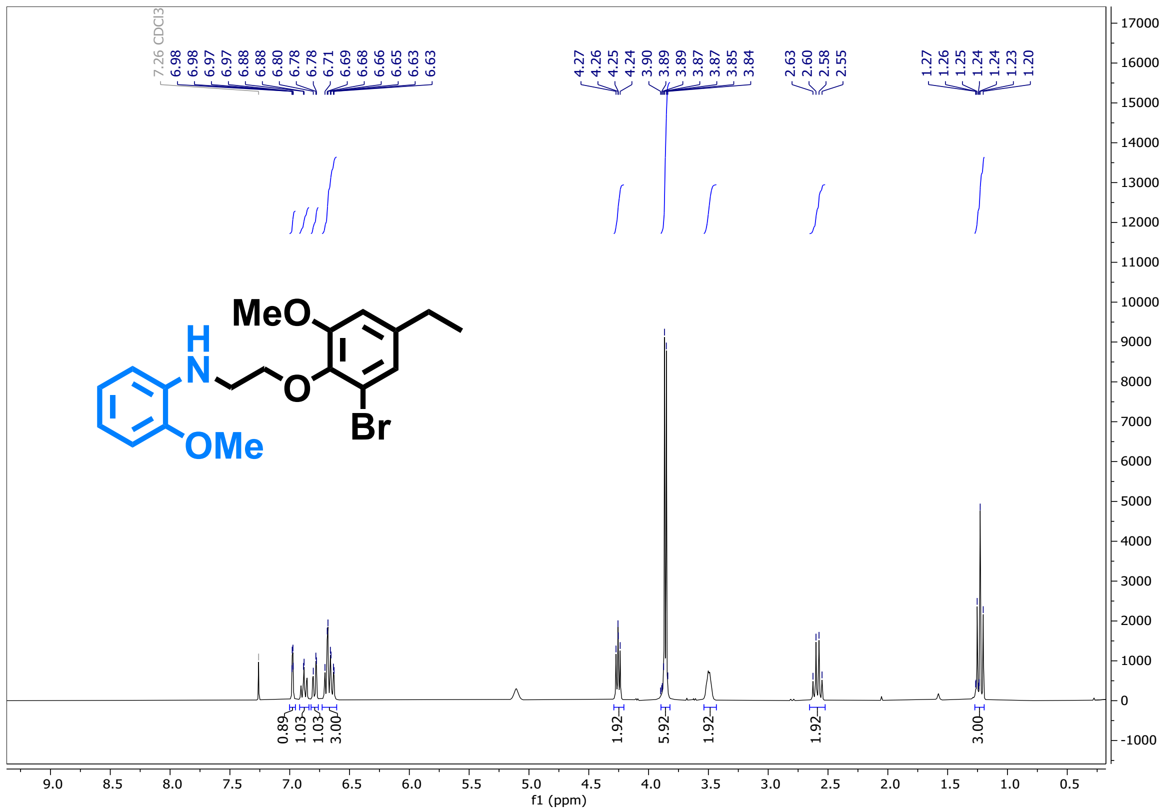

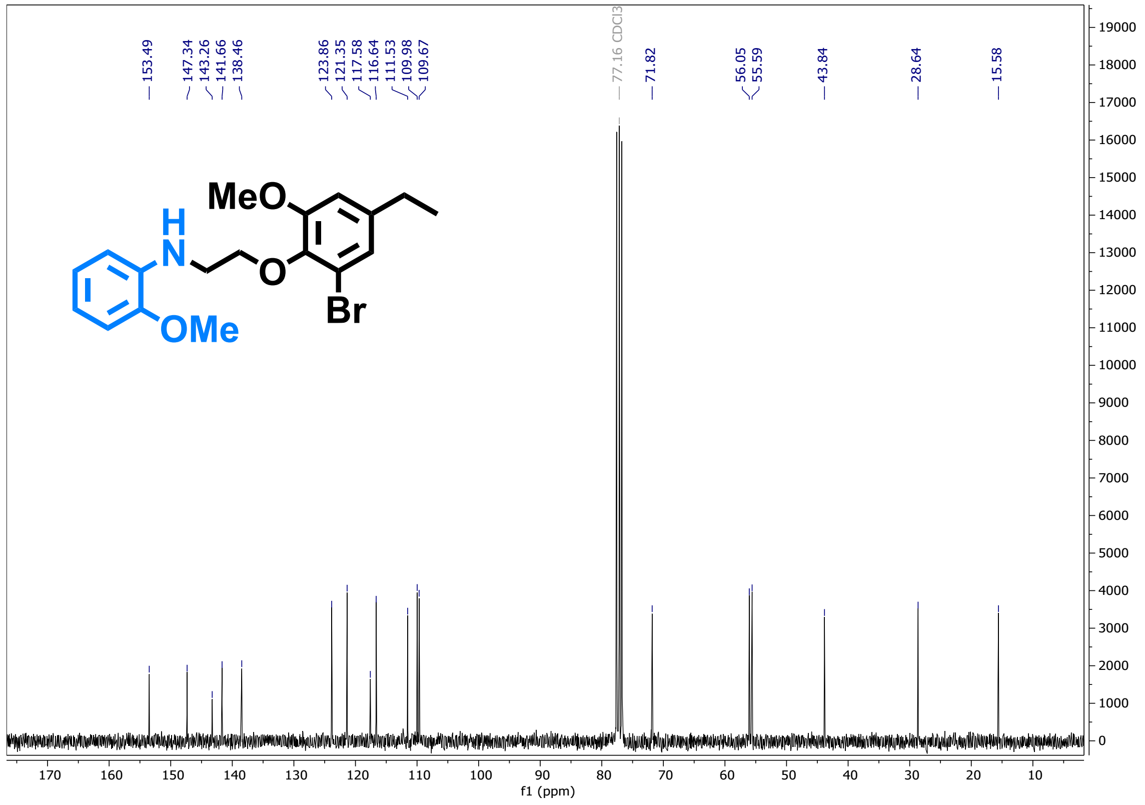

Fig. S27 ^{1}H and ^{13}C NMR spectra of compound **3Gc2**.

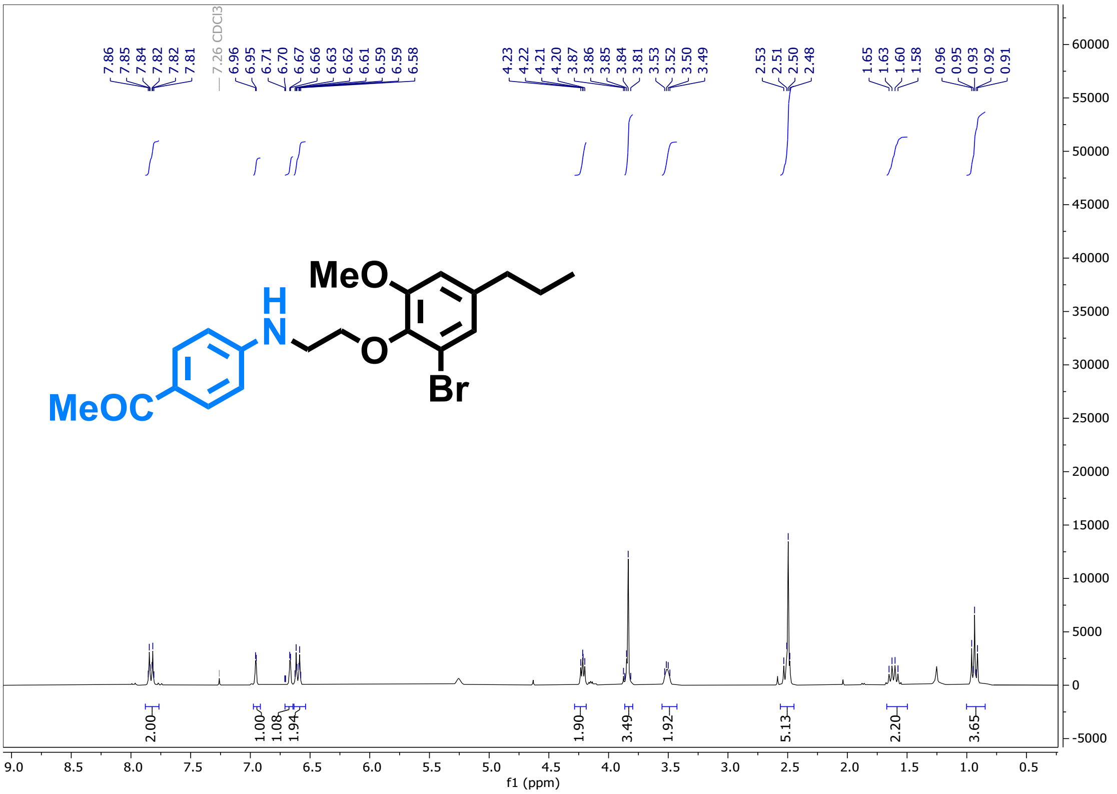

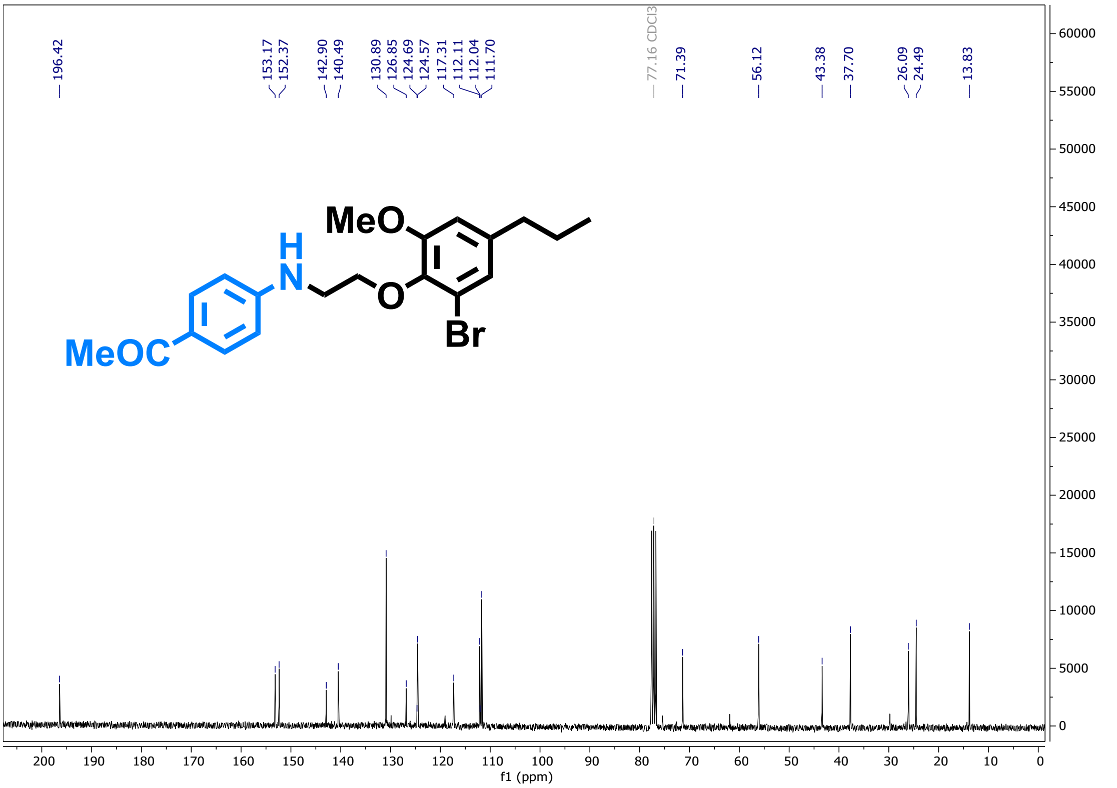

Fig. S28 ^{1}H and ^{13}C NMR spectra of compound **2Gc3**.

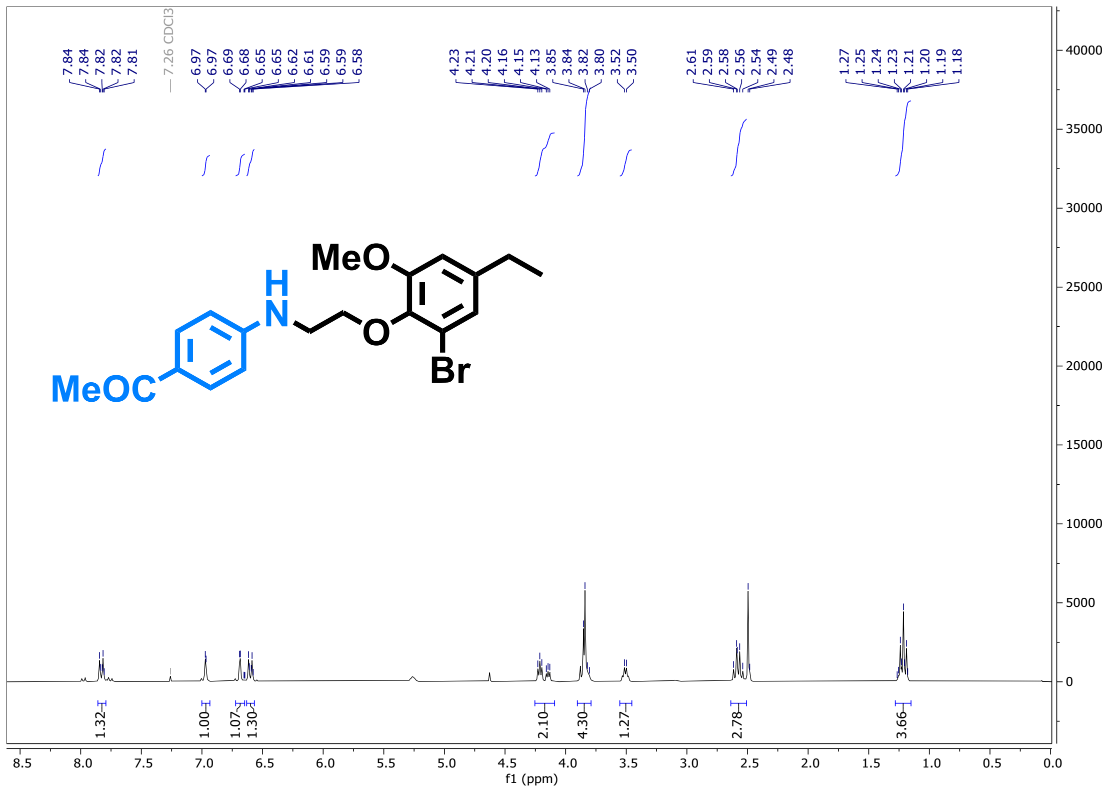

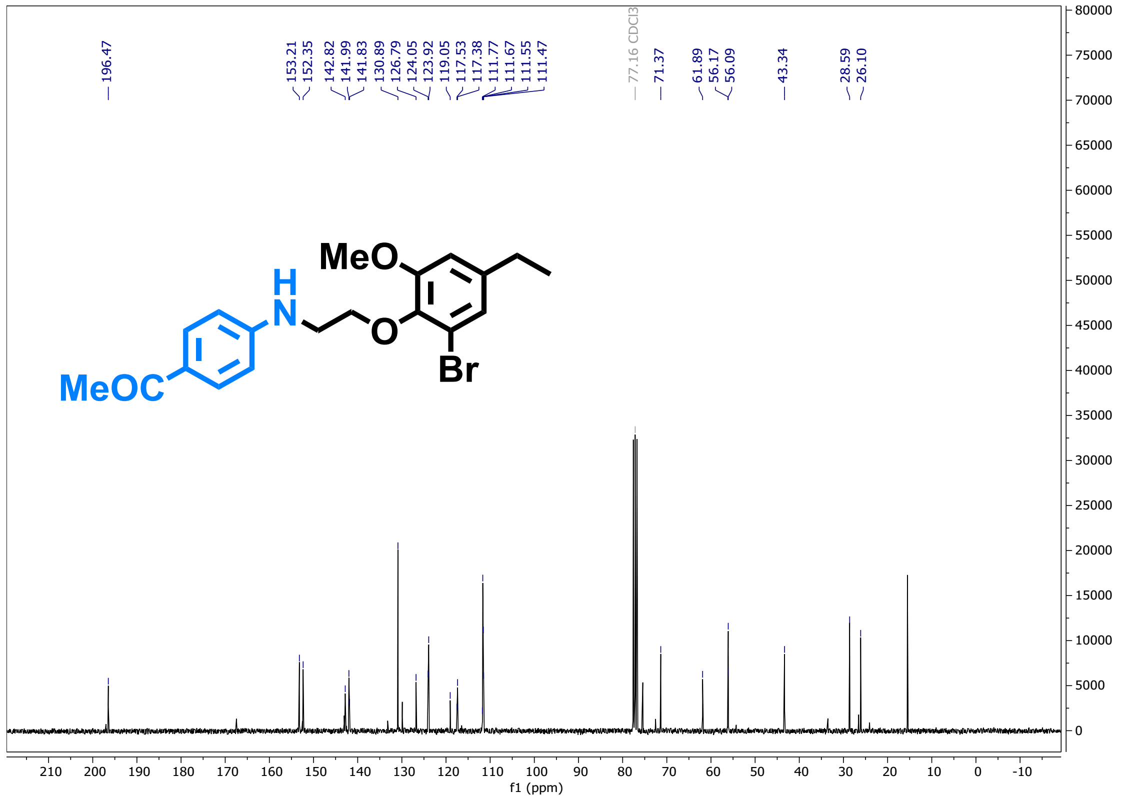

Fig. S29 ^{1}H and ^{13}C NMR spectra of compound **3Gc3**.

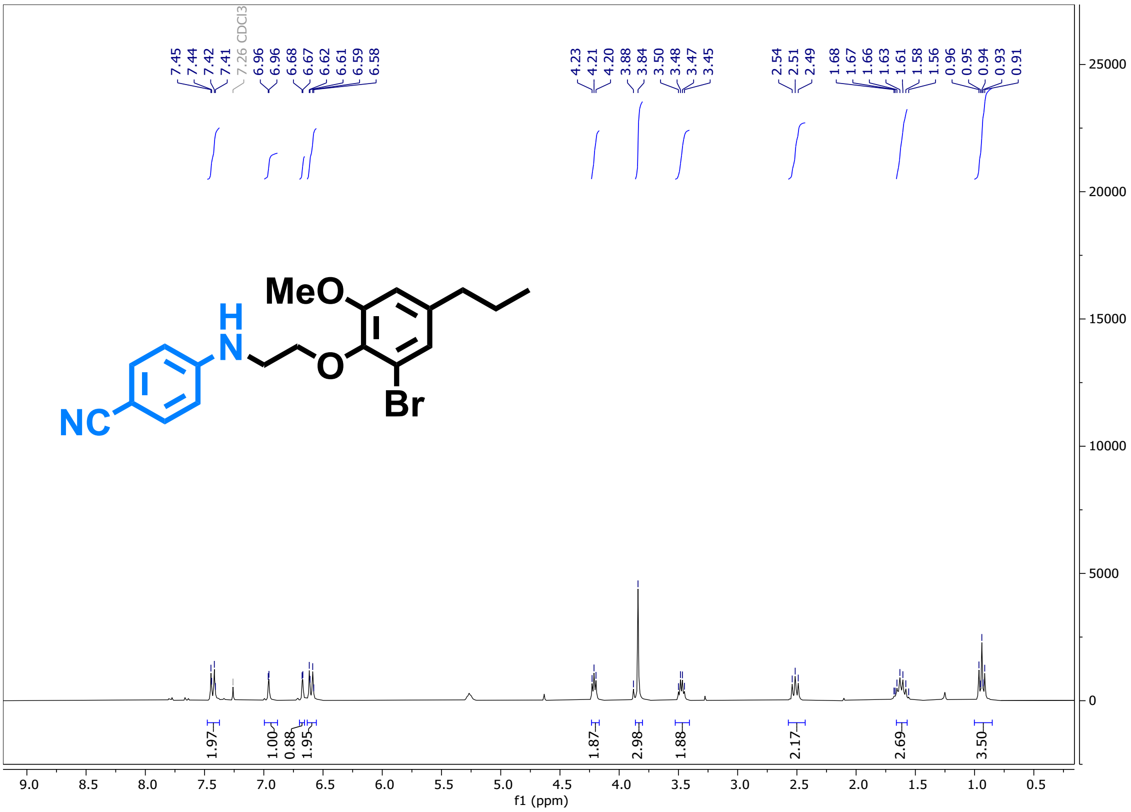

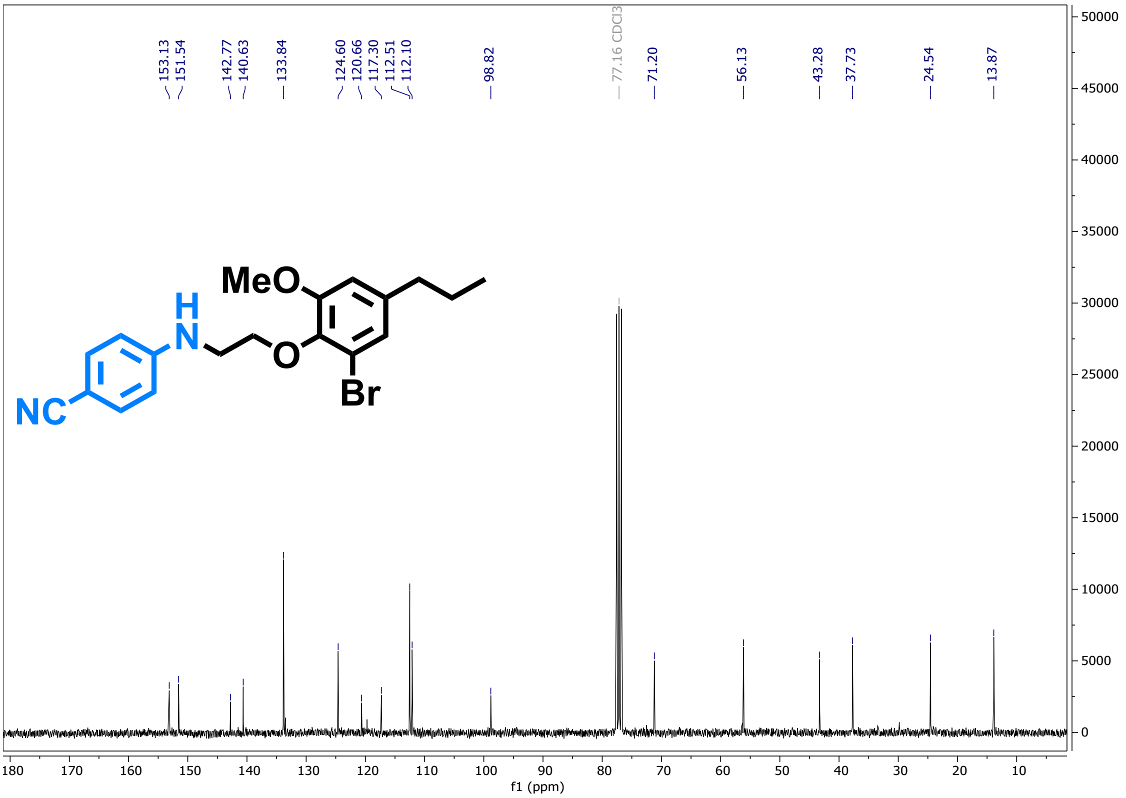

Fig. S30 ^{1}H and ^{13}C NMR spectra of compound **2Gc4**.

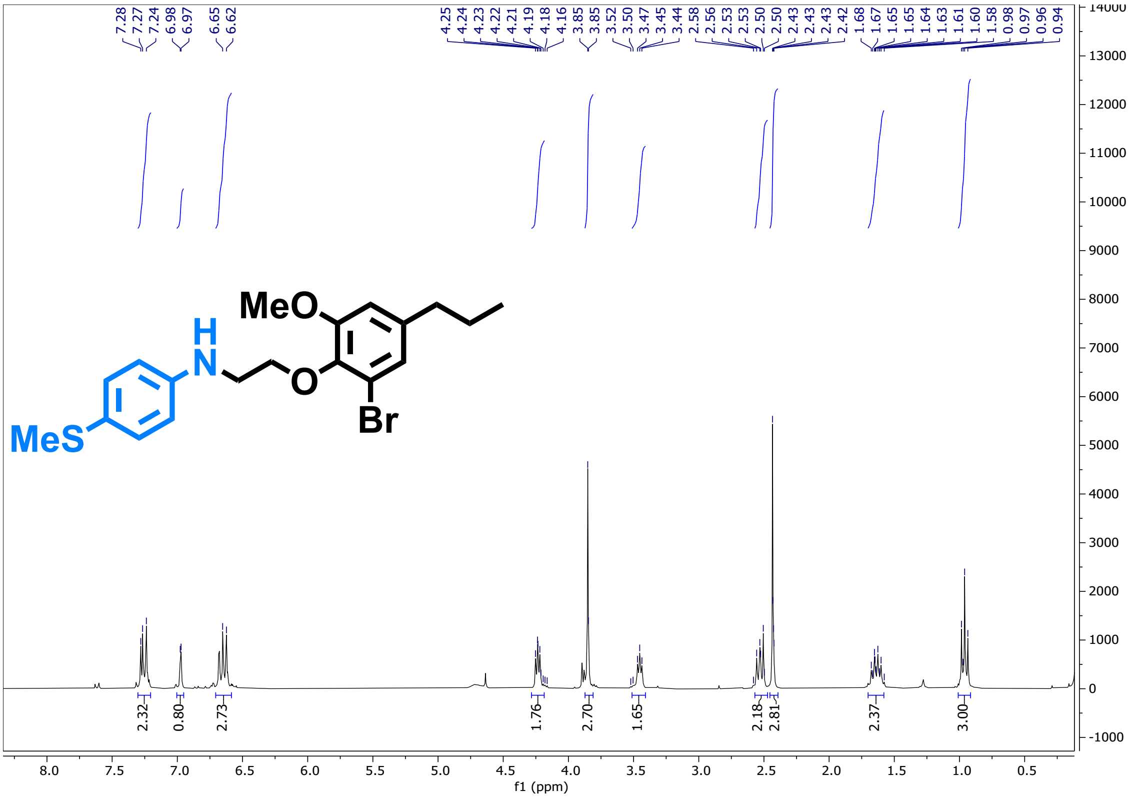

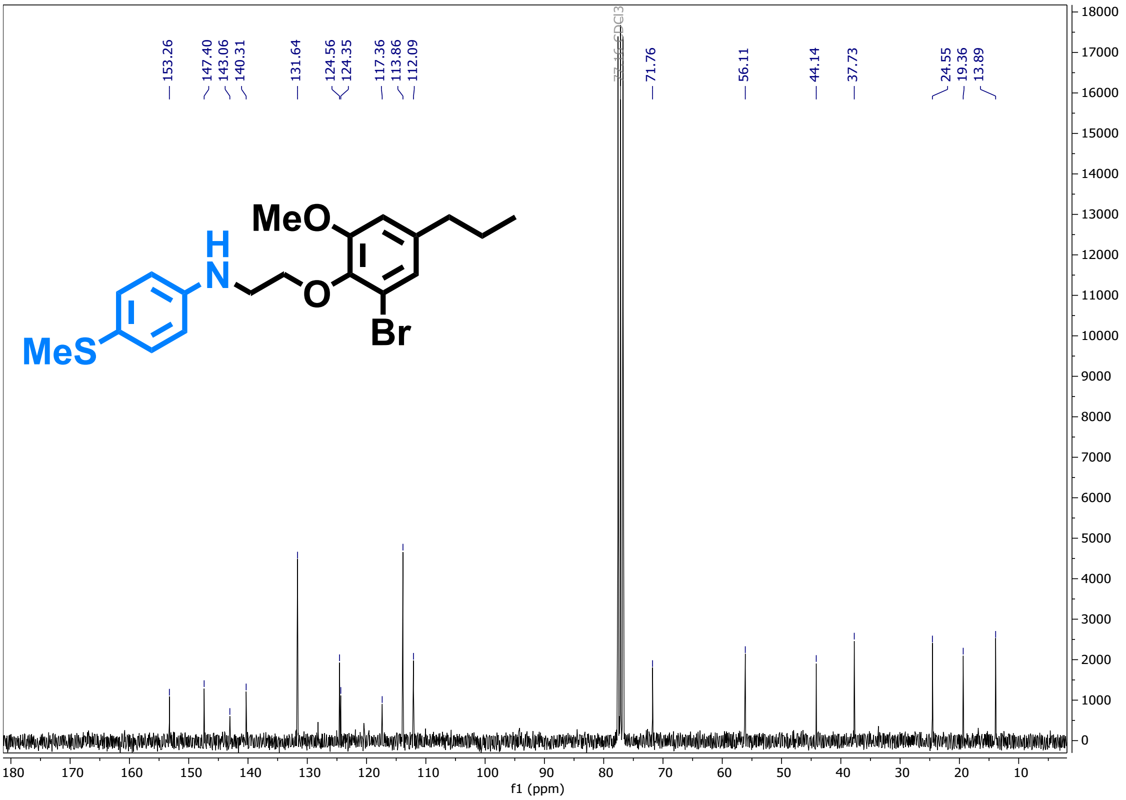

Fig. S31 ^{1}H and ^{13}C NMR spectra of compound **2Gc5**.

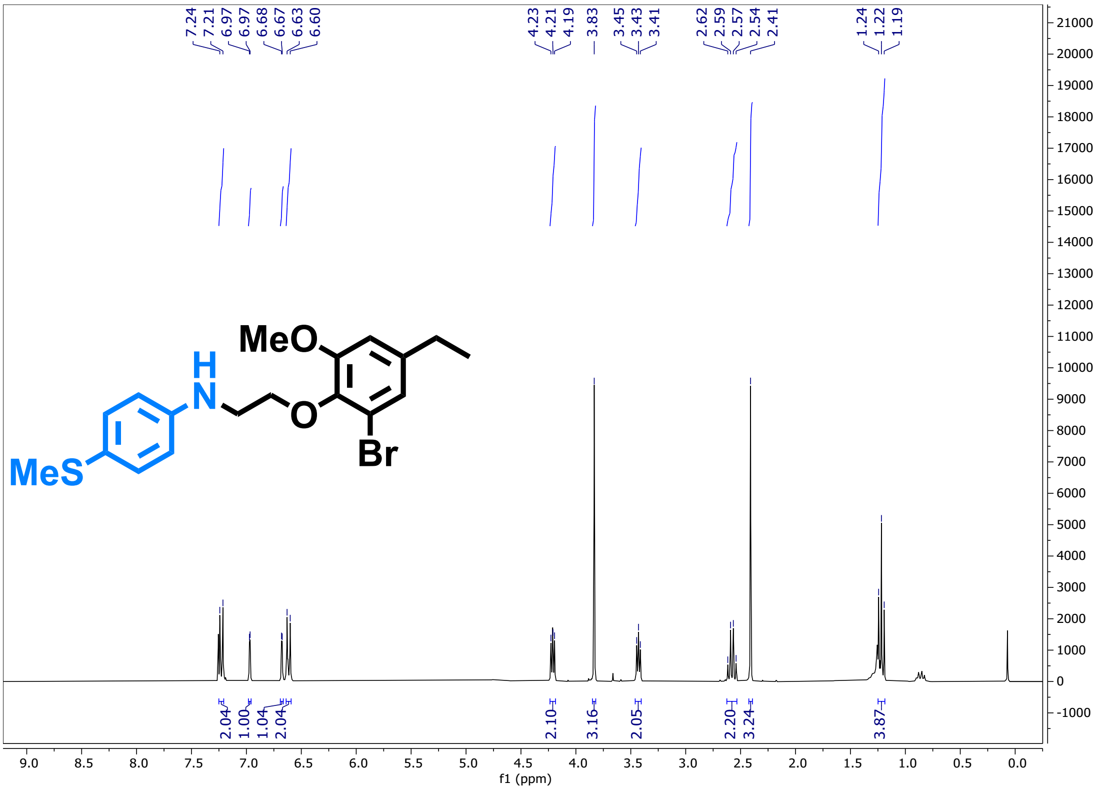

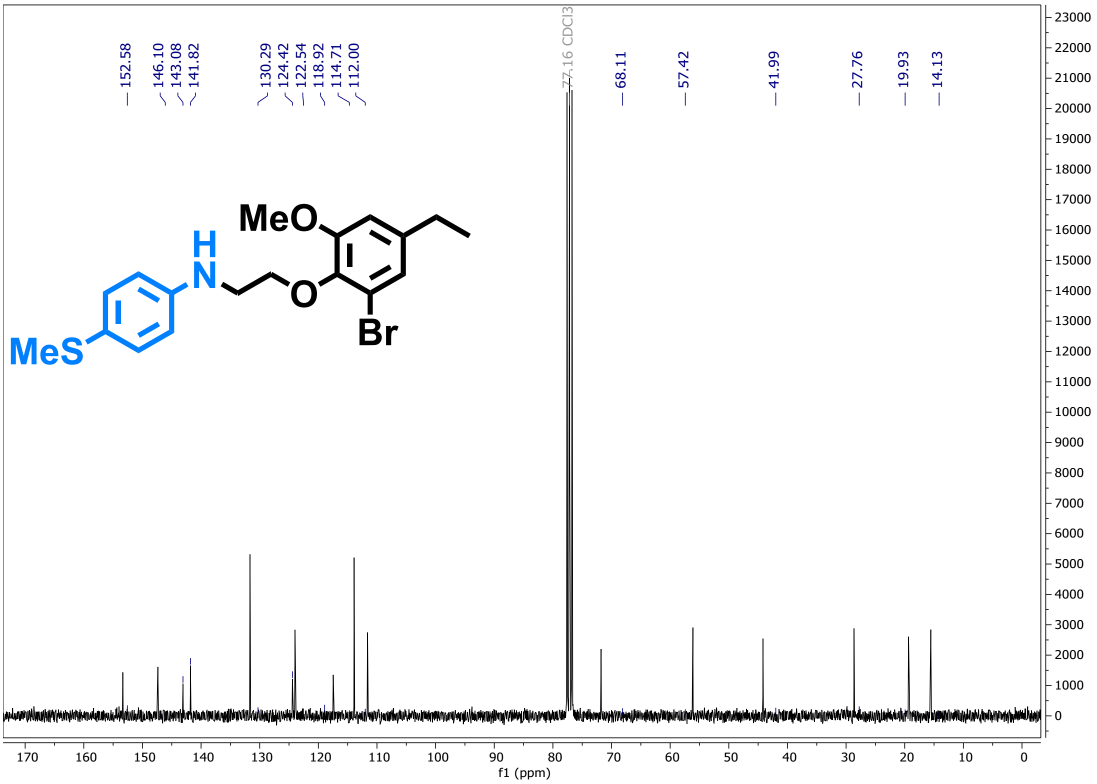

Fig. S32 ^{1}H and ^{13}C NMR spectra of compound **3Gc5**.

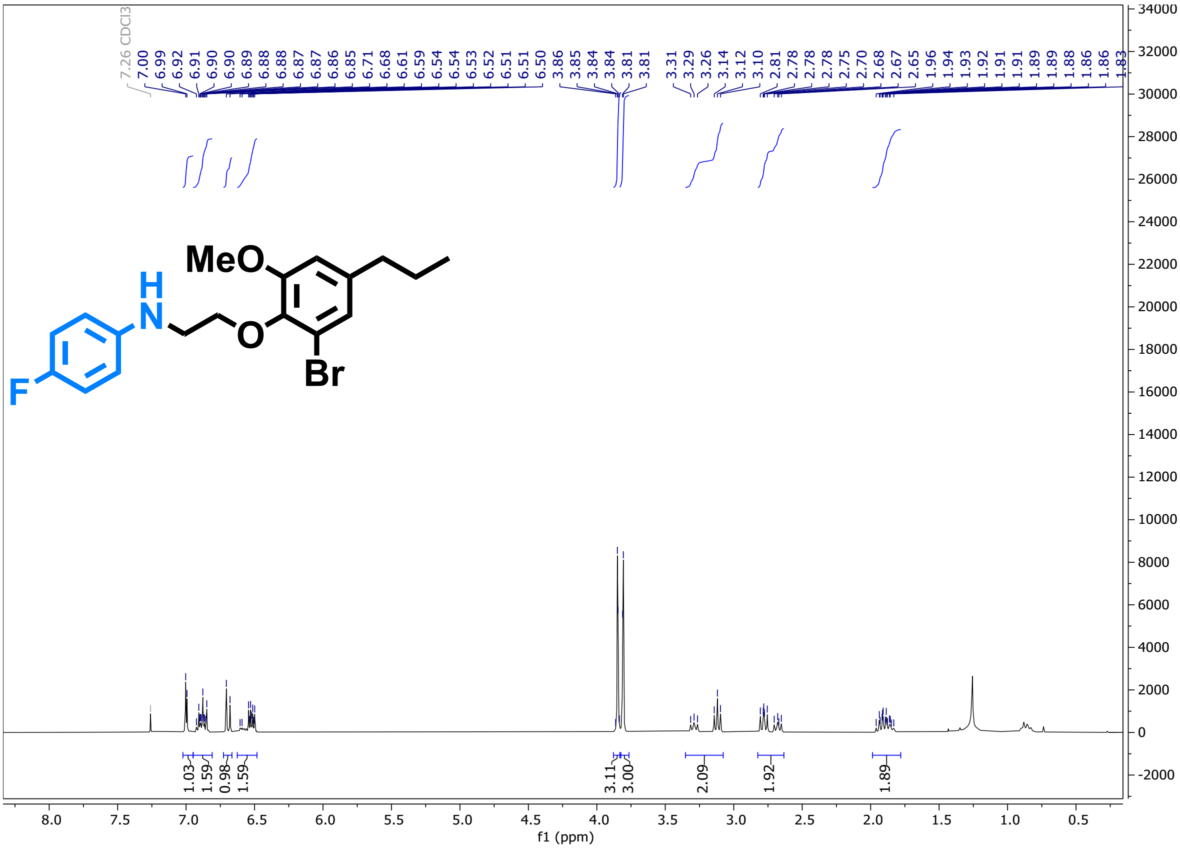

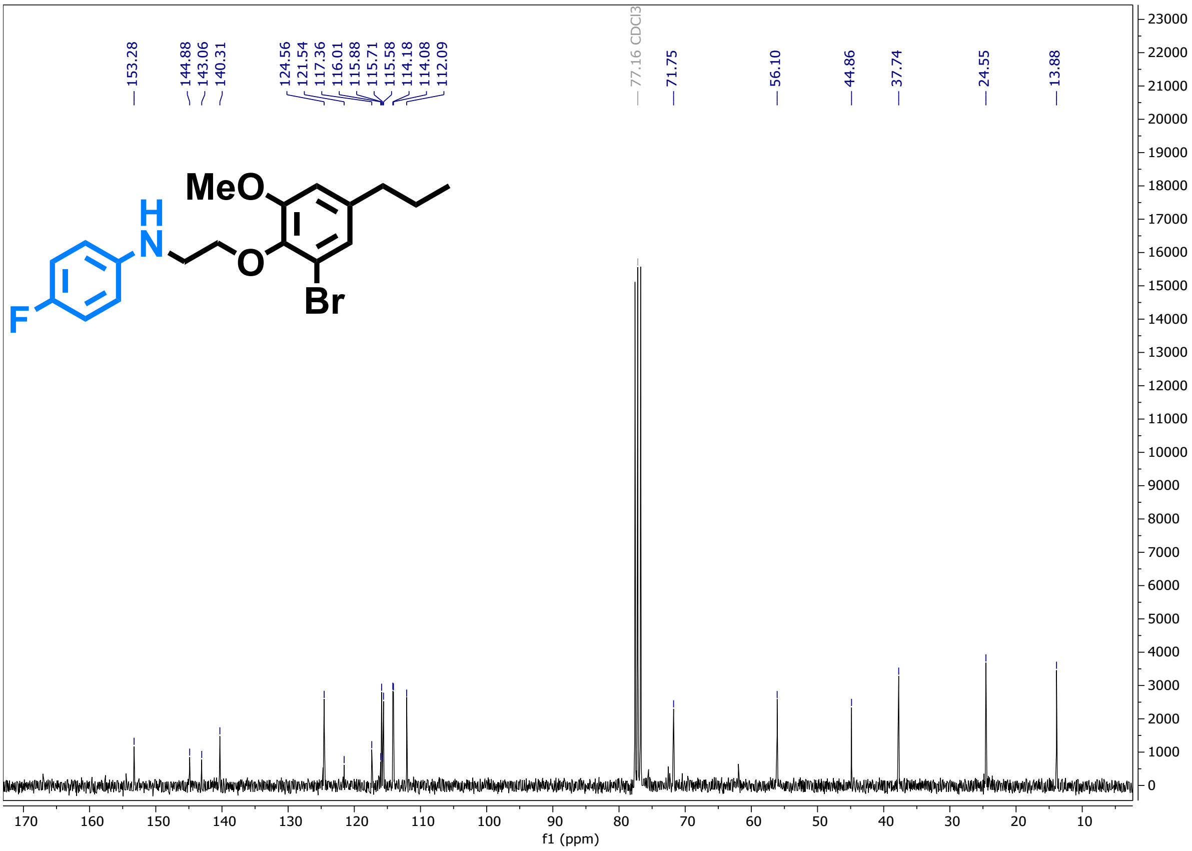

Fig. S33 ^{1}H and ^{13}C NMR spectra of compound **2Gc6**.

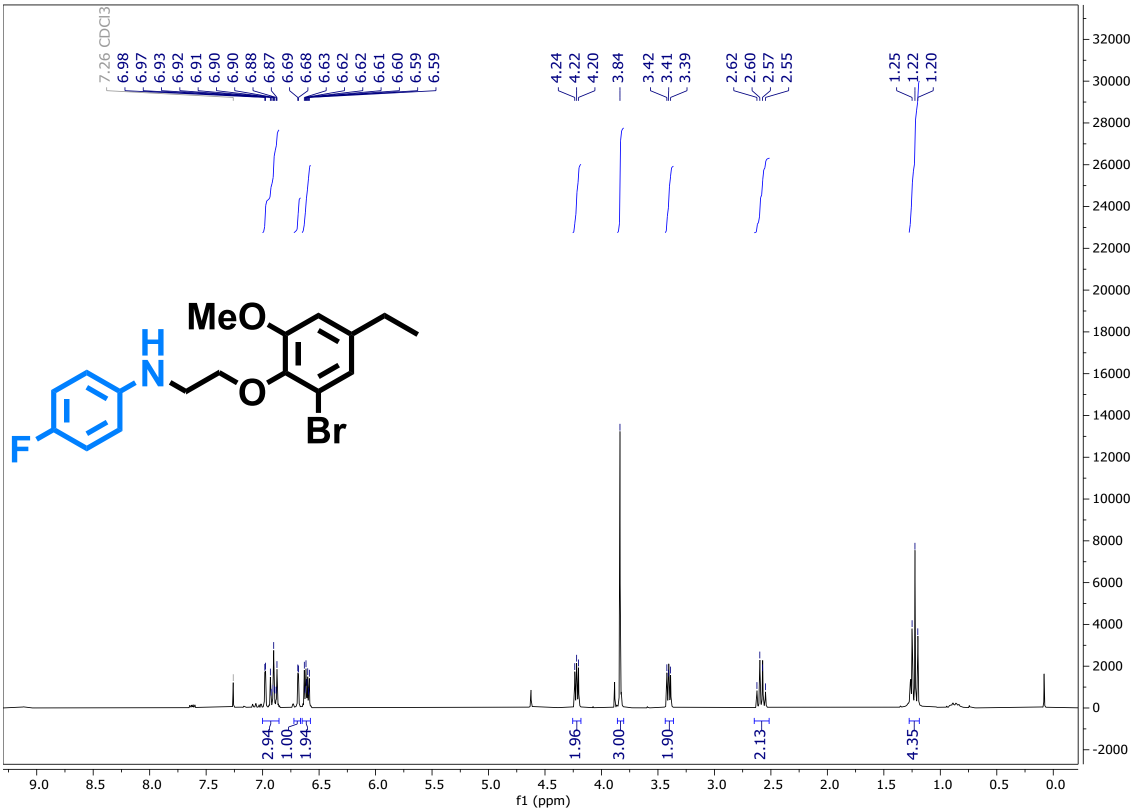

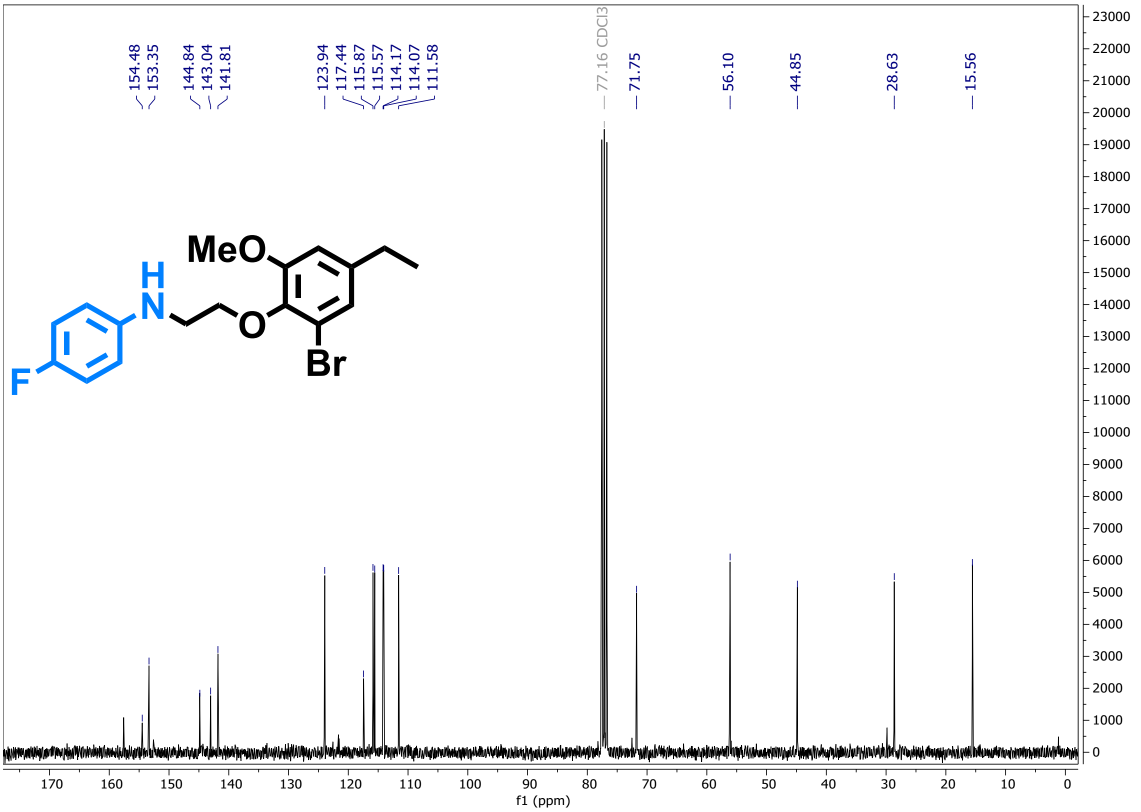

Fig. S34 ^{1}H and ^{13}C NMR spectra of compound **3Gc6**.

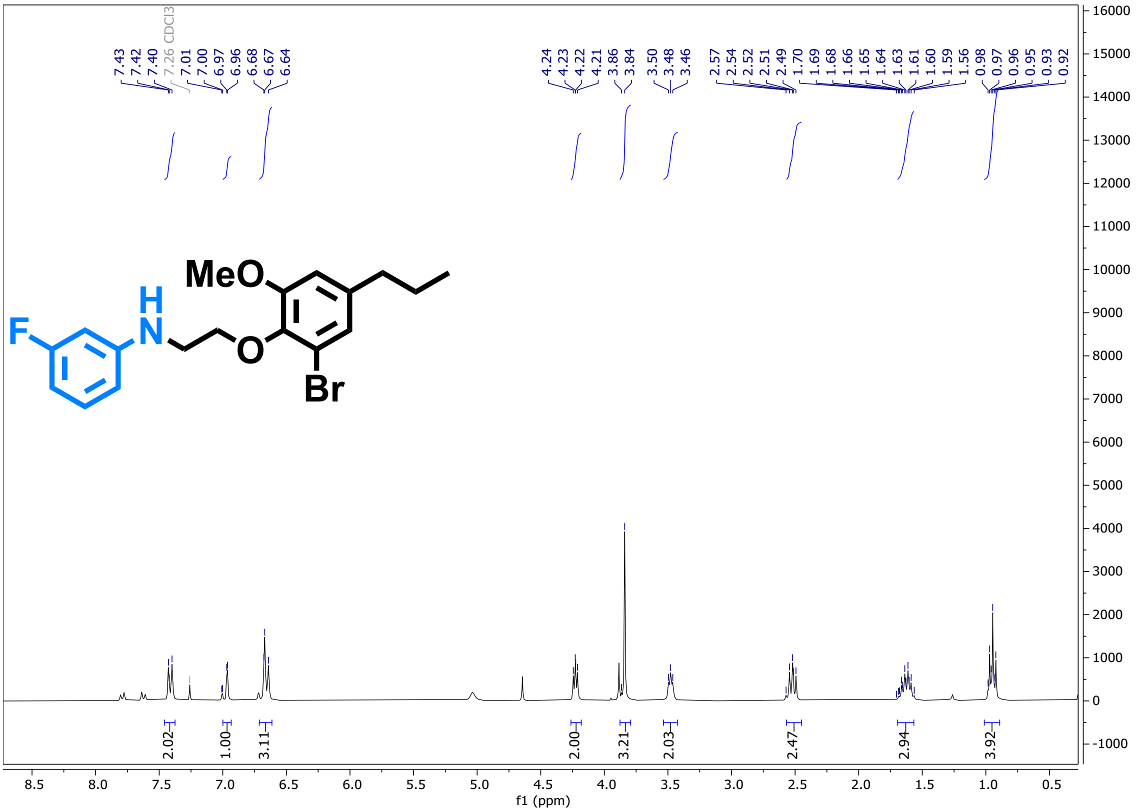

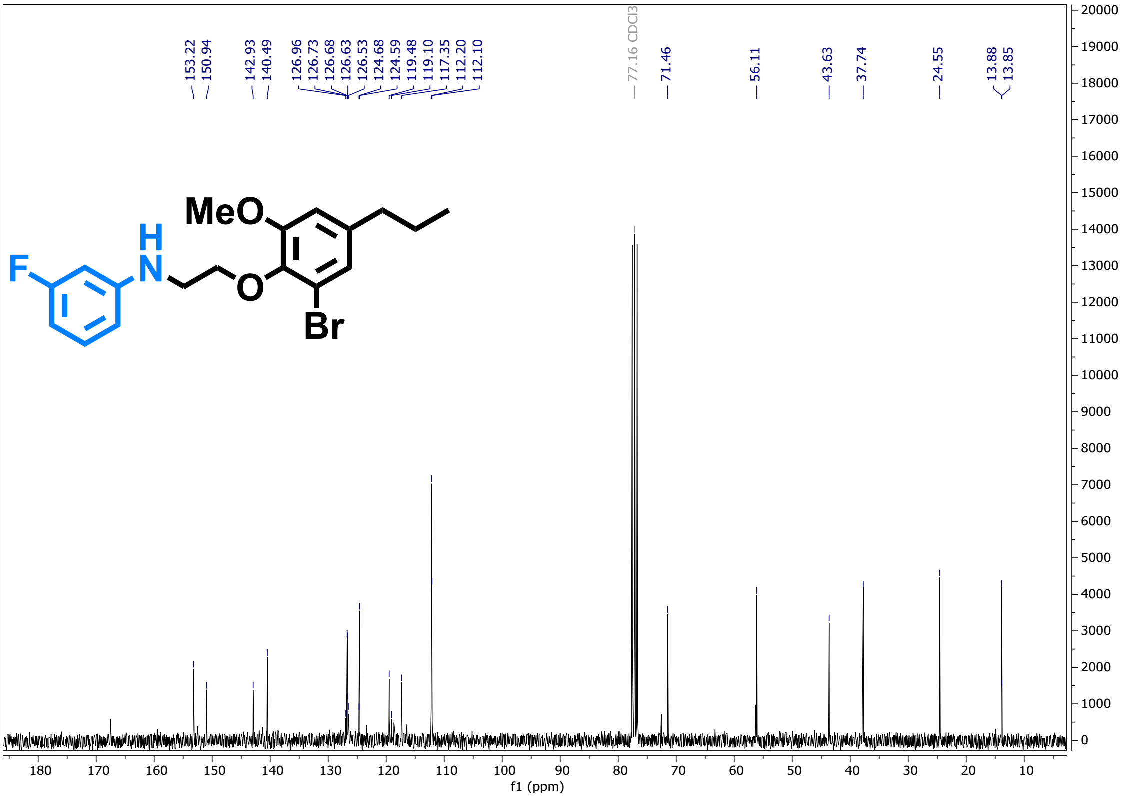

Fig. S35 ^{1}H and ^{13}C NMR spectra of compound **2Gc7**.

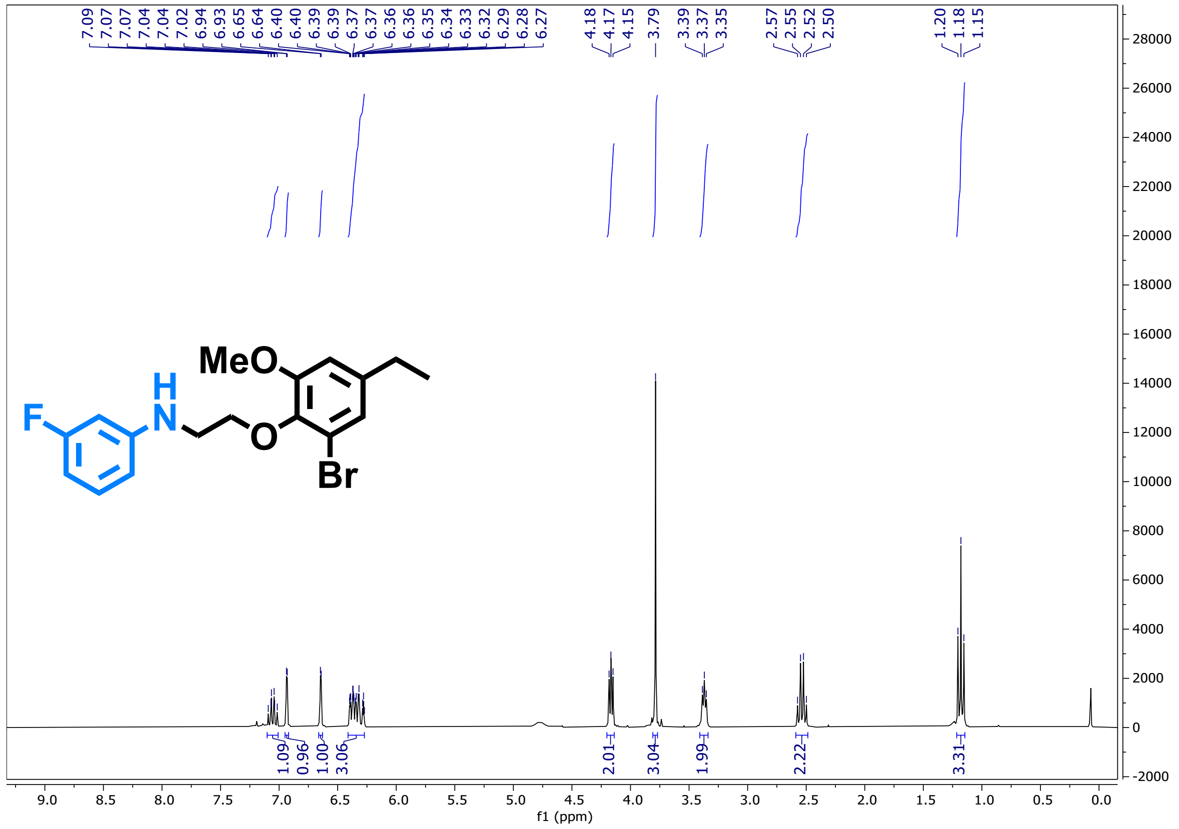

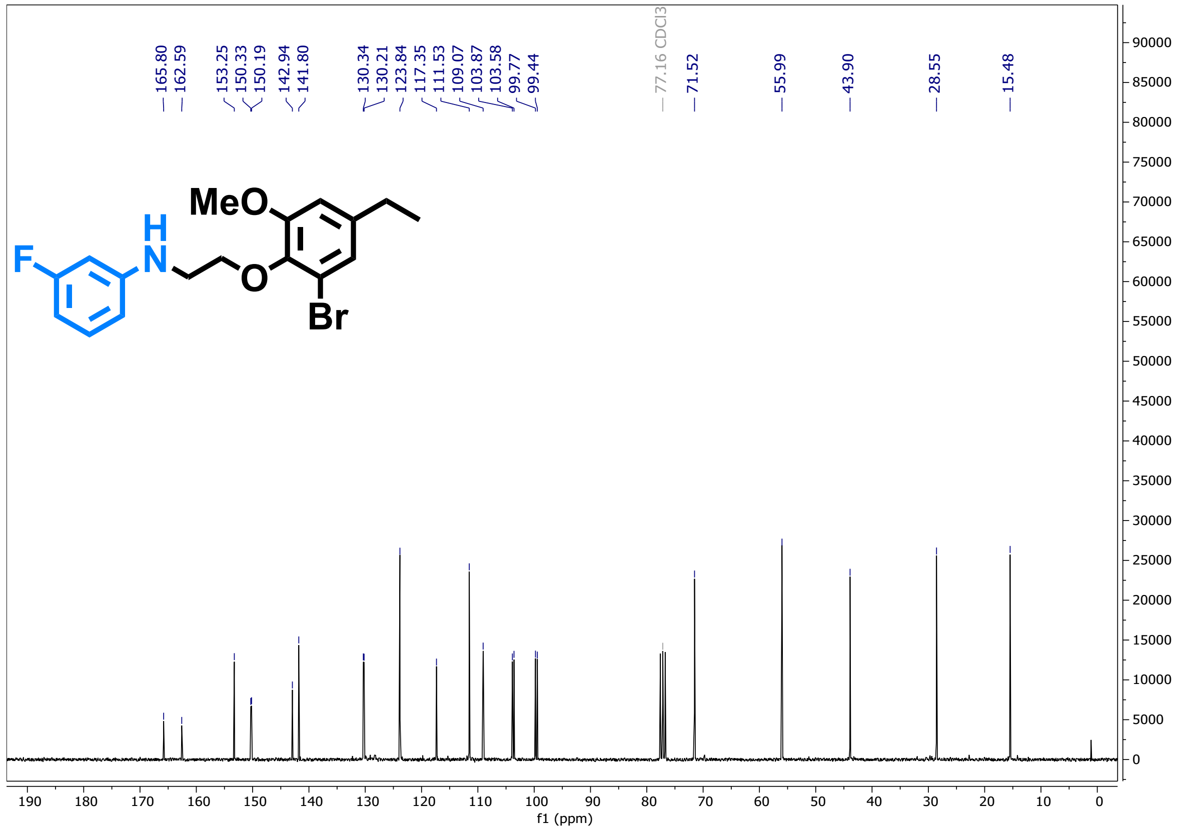

Fig. S36 ^{1}H and ^{13}C NMR spectra of compound **3Gc7**.

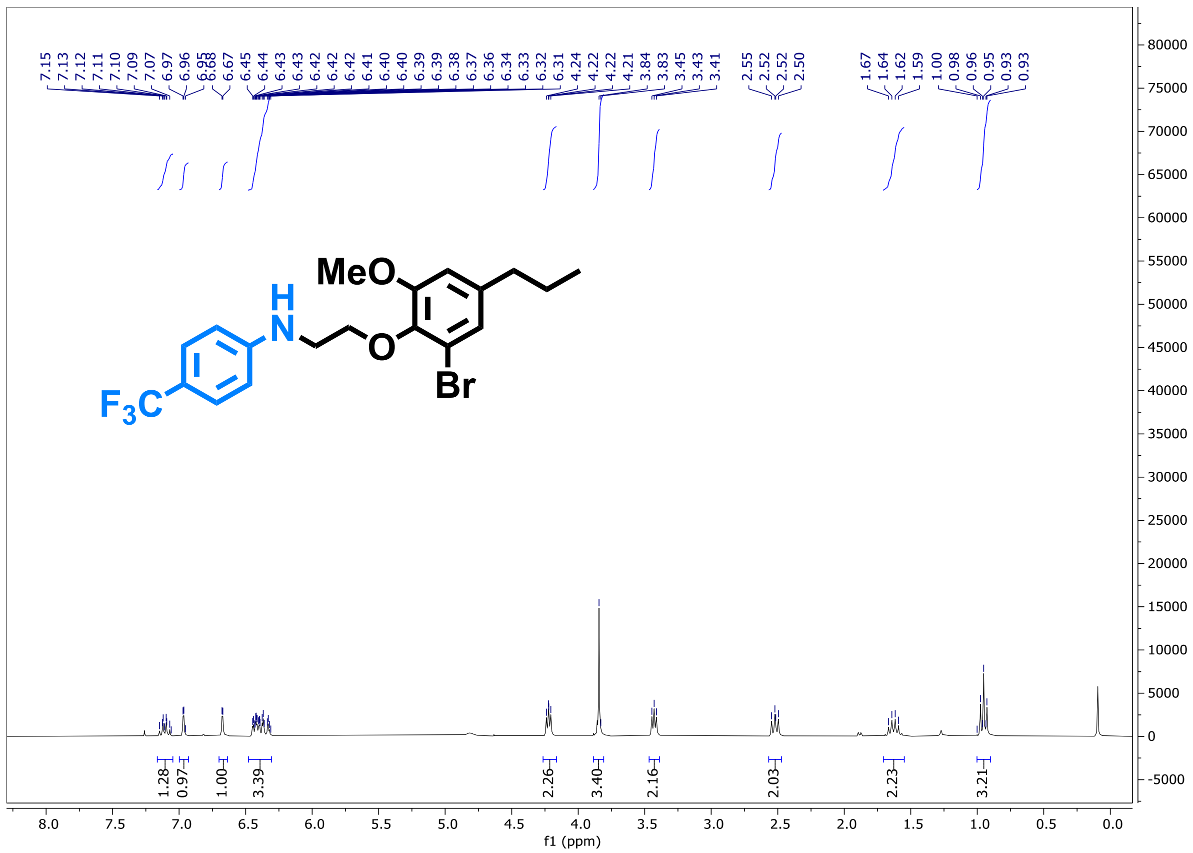

7.15
7.13
7.12
7.11
7.10
7.09
7.07
6.97
6.96
6.95
6.68
6.67
6.45
6.44
6.43
6.43
6.42
6.42
6.42
6.41
6.40
6.40
6.39
6.39
6.38
6.37
6.36
6.34
6.33
6.32
6.31
4.24
4.22
4.22
4.21
3.84
3.83
3.45
3.43
3.41
2.55
2.52
2.52
2.50
1.67
1.64
1.62
1.59
1.00
0.98
0.96
0.95
0.93
0.93
MeO
H
N
O
Br
F3C
1.28
0.97
1.00
3.39
2.26
3.40
2.16
2.03
2.23
3.21
f1 (ppm)

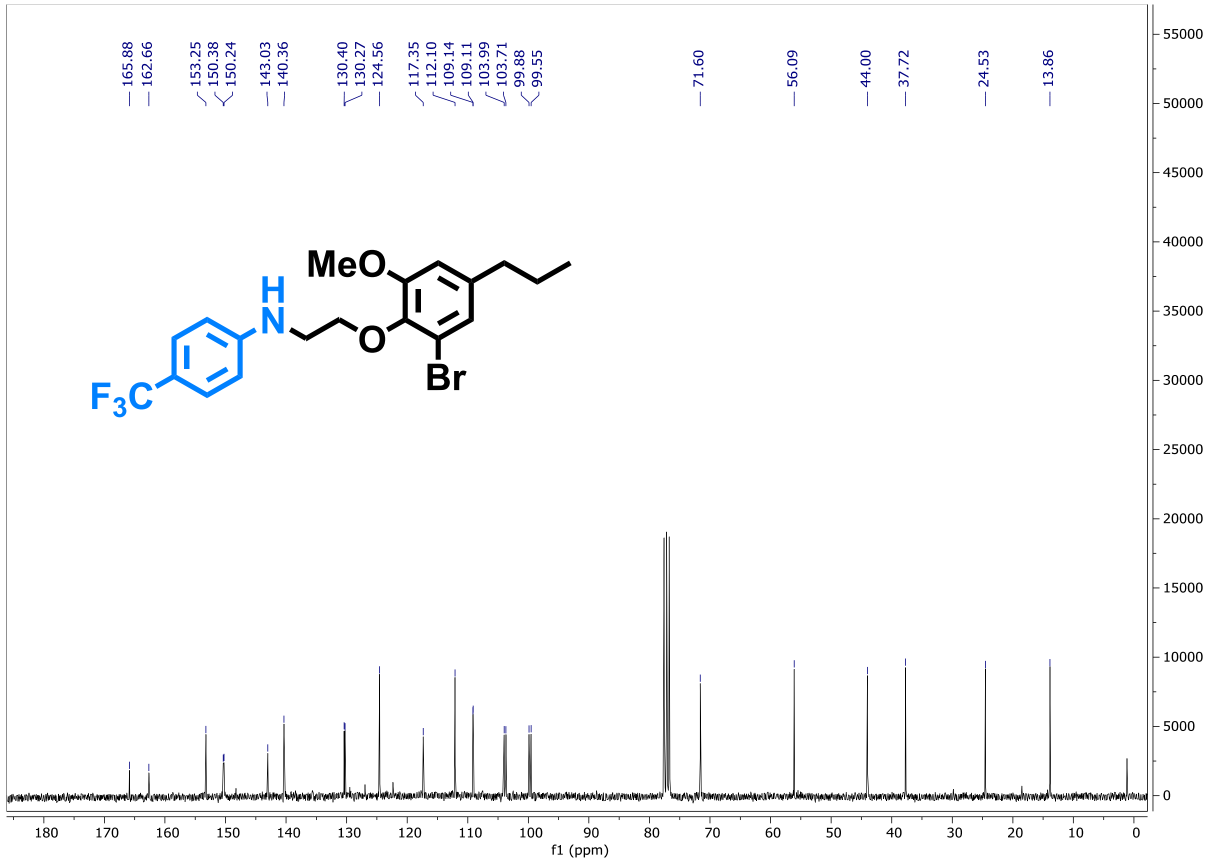

165.88
162.66
153.25
150.38
150.24
143.03
140.36
130.40
130.27
124.56
117.35
112.10
109.14
109.11
103.99
103.71
99.88
99.55
71.60
56.09
44.00
37.72
24.53
13.86
MeO
H
N
O
Br
F3C
f1 (ppm)

Fig. S37 ^{1}H and ^{13}C NMR spectra of compound **2Gc8**.

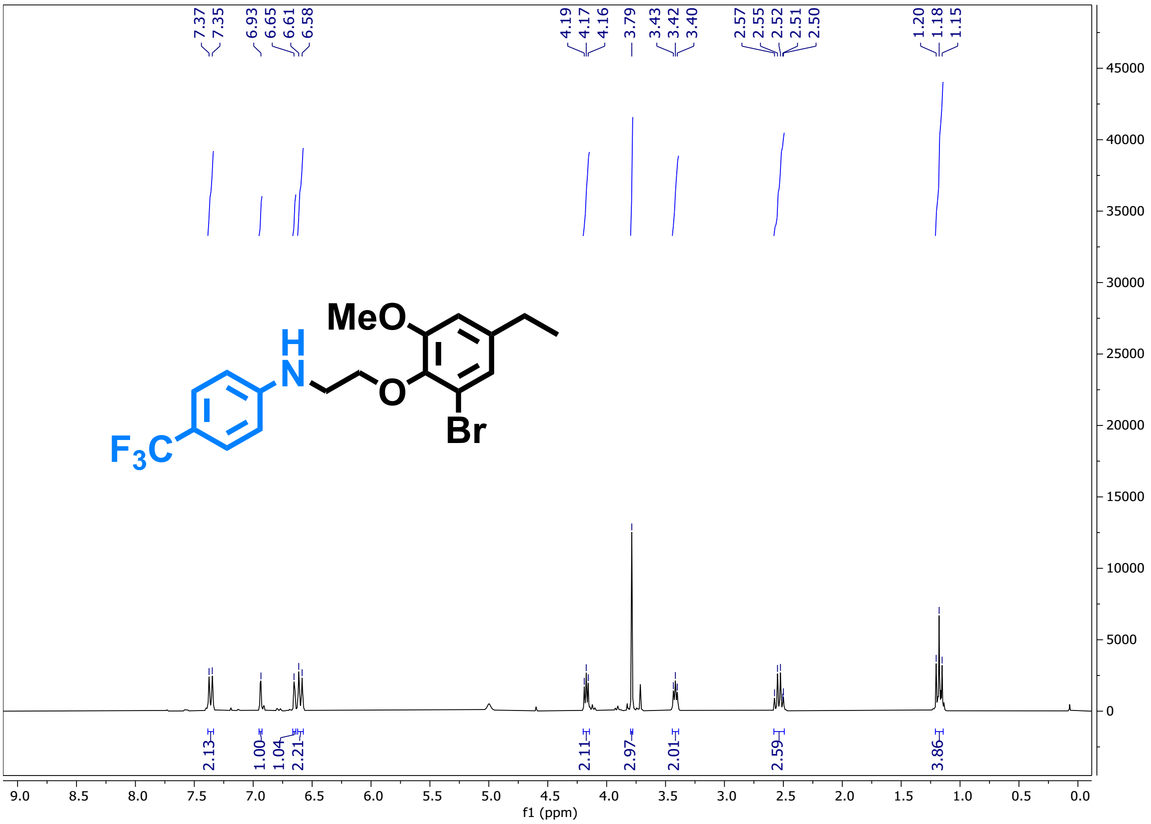

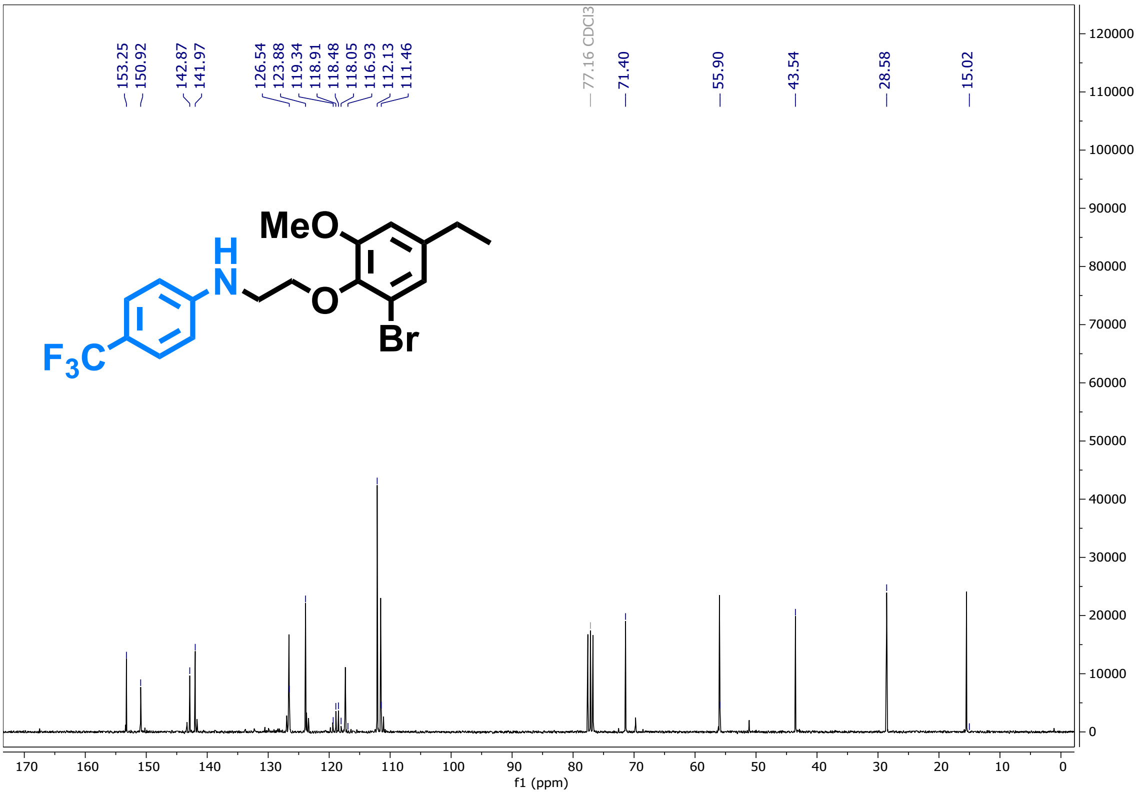

Fig. S38 ^{1}H and ^{13}C NMR spectra of compound **3Gc8**.

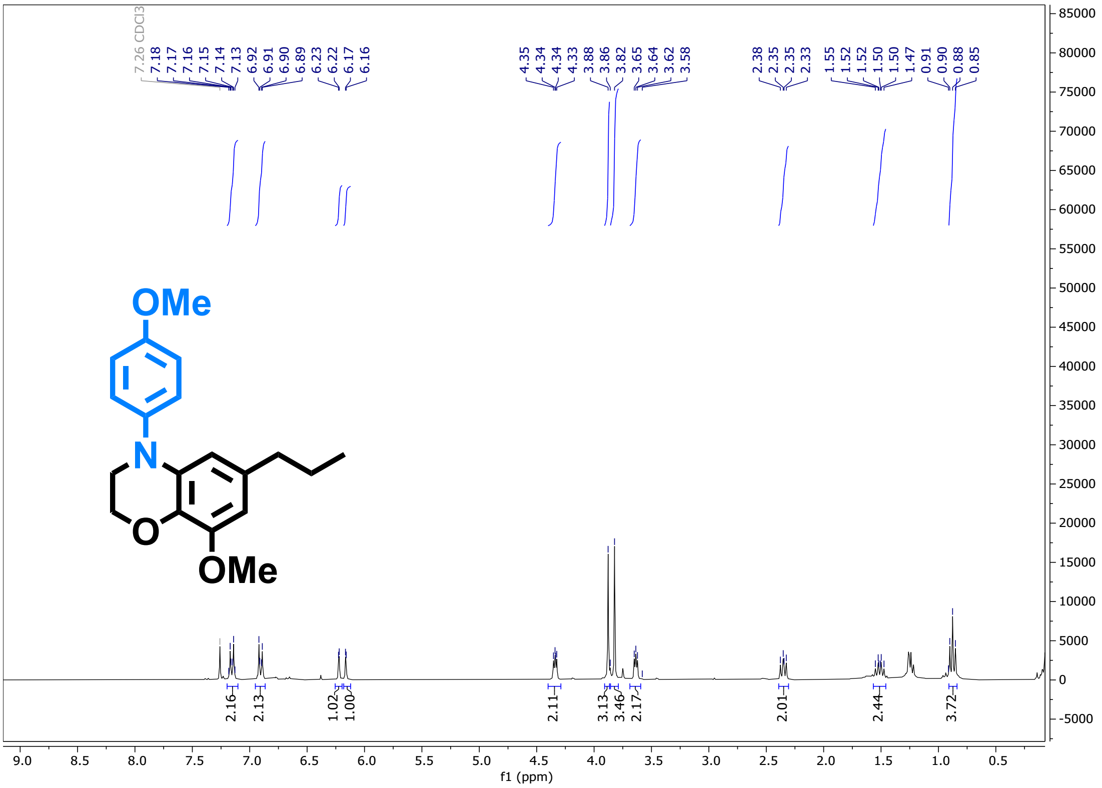

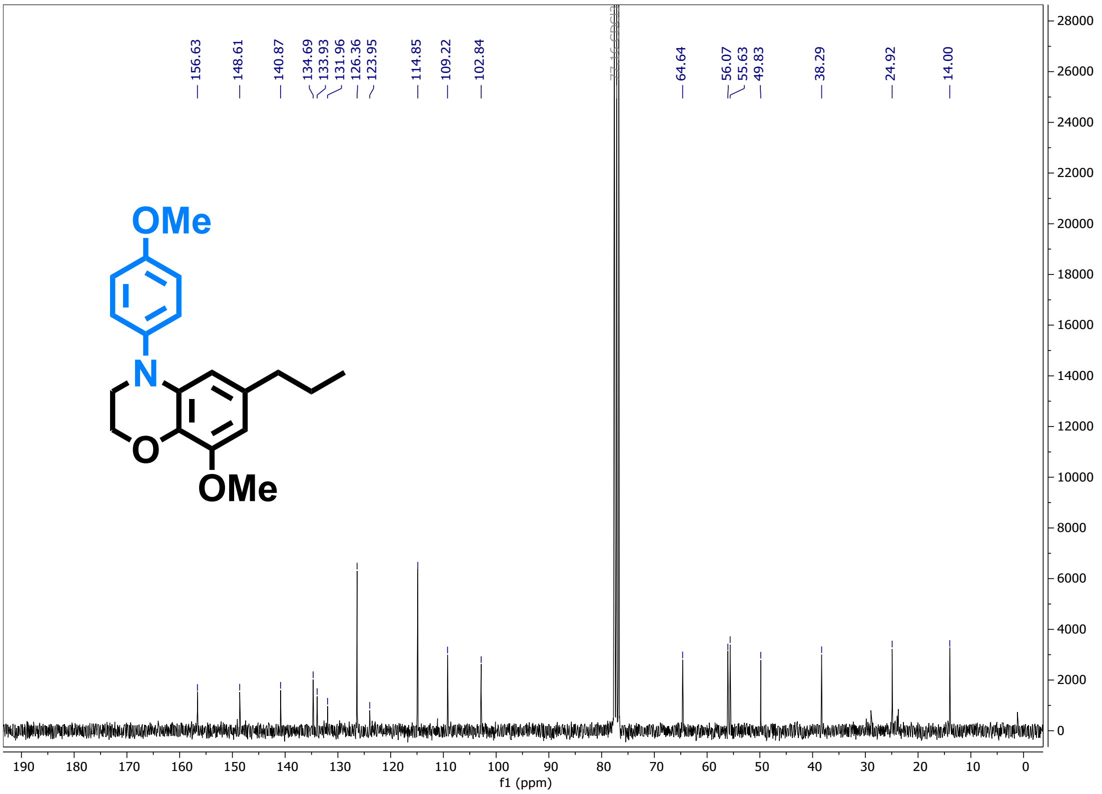

Fig. S39 ^{1}H and ^{13}C NMR spectra of compound **2Gd1**.

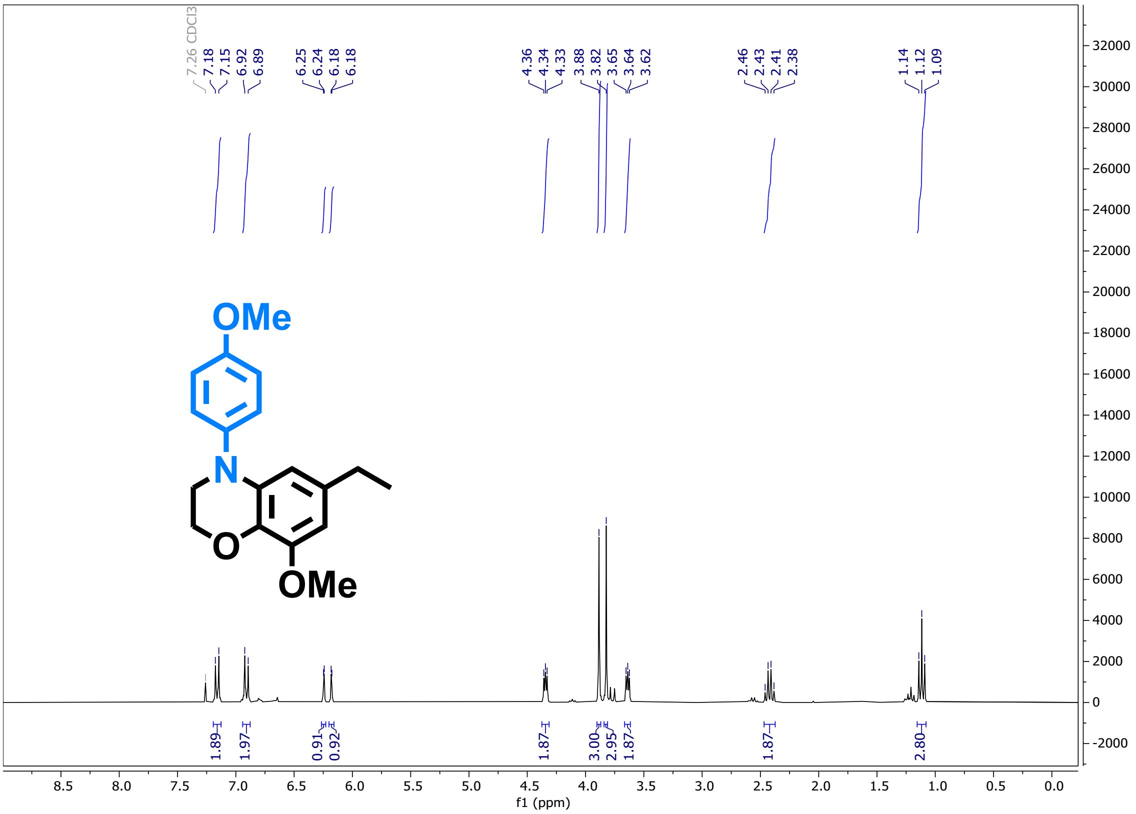

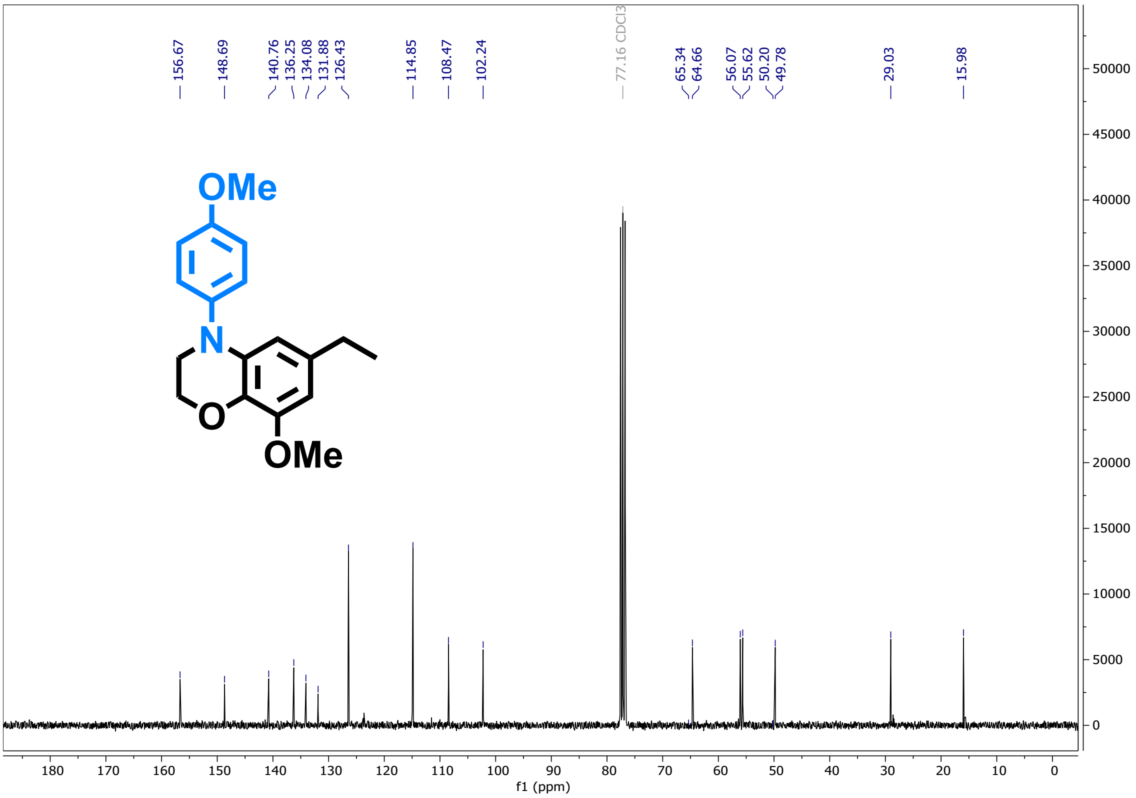

Fig. S40 ^{1}H and ^{13}C NMR spectra of compound **3Gd1**.

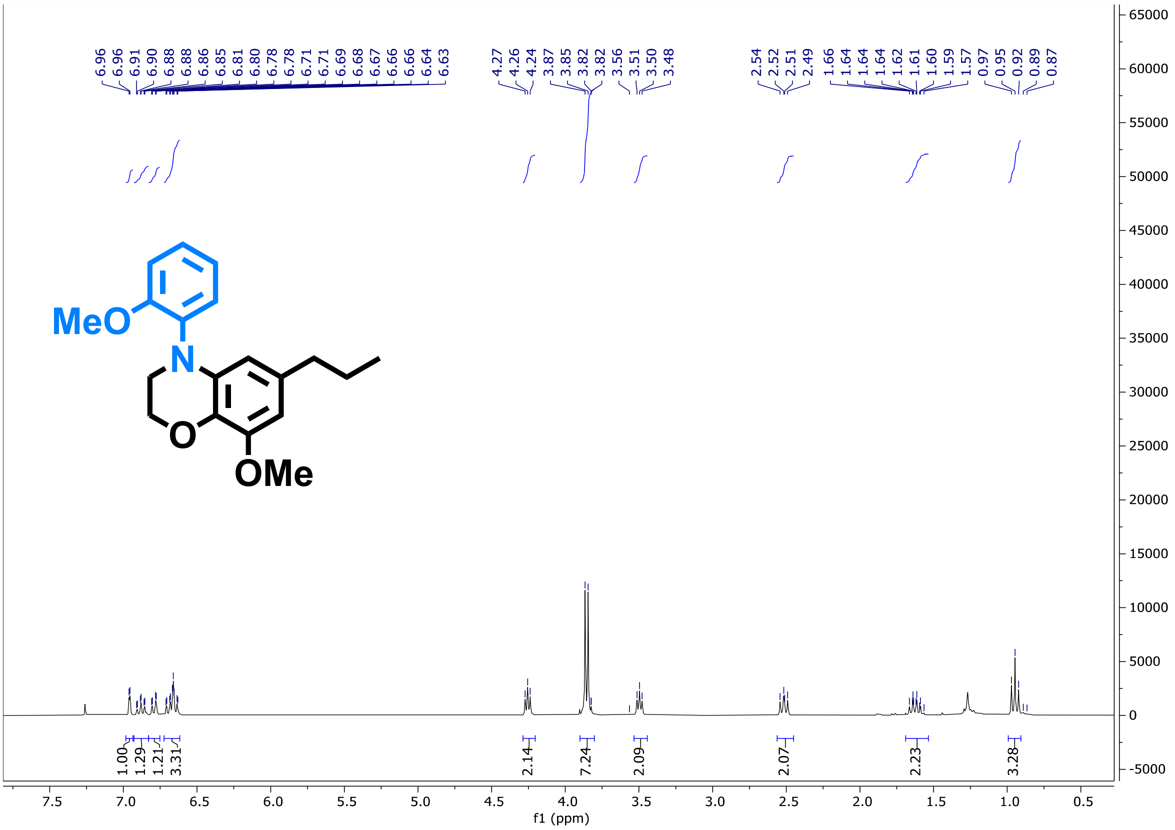

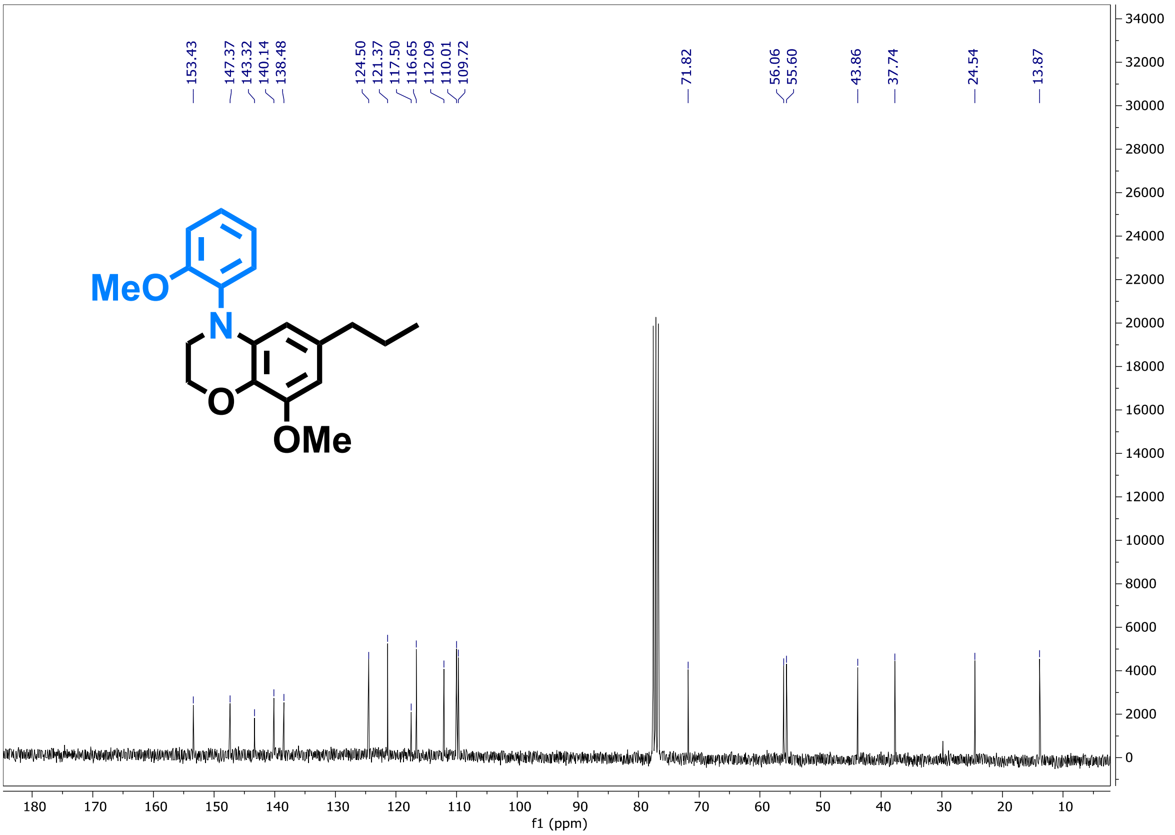

Fig. S41 ^{1}H and ^{13}C NMR spectra of compound **2Gd2**.

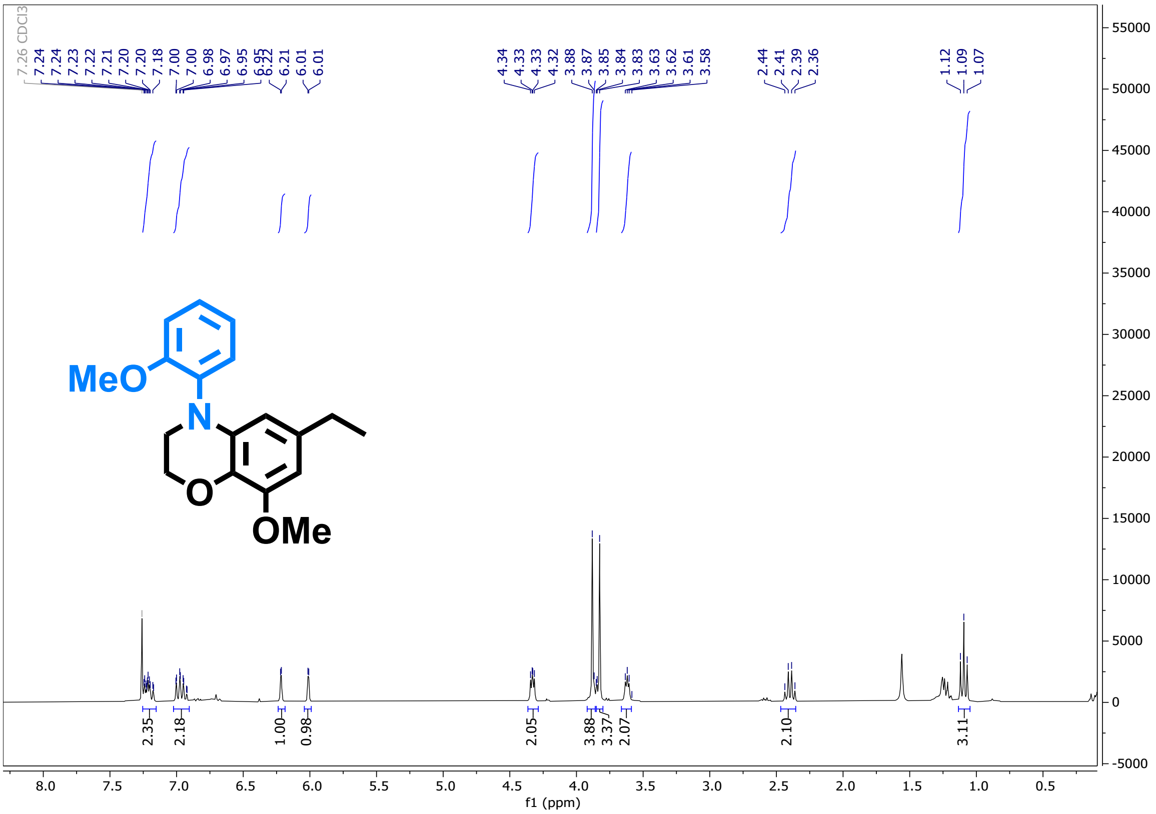
7.26 CDCl3
7.24
7.24
7.23
7.22
7.21
7.20
7.20
7.18
7.00
7.00
6.98
6.97
6.95
6.95
6.22
6.21
6.01
6.01
4.34
4.33
4.33
4.32
3.88
3.87
3.85
3.84
3.83
3.63
3.62
3.61
3.58
2.44
2.41
2.39
2.36
1.12
1.09
1.07
MeO
N
O
OMe
2.35
2.18
1.00
0.98
2.05
3.88
3.37
2.07
2.10
3.11
f1 (ppm)

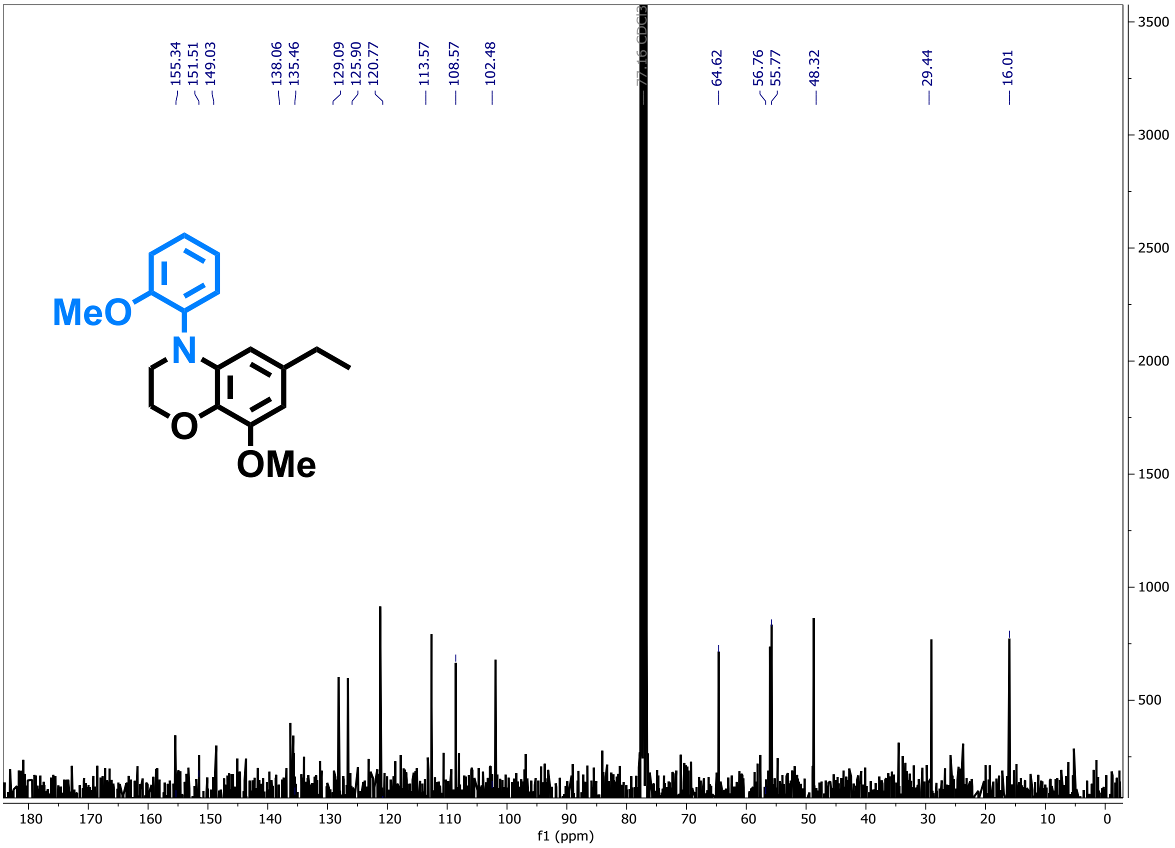
155.34
151.51
149.03
138.06
135.46
129.09
125.90
120.77
113.57
108.57
102.48
77.16 CDCl3
64.62
56.76
55.77
48.32
29.44
16.01
MeO
N
O
OMe
f1 (ppm)

Fig. S42 ^{1}H and ^{13}C NMR spectra of compound **3Gd2**.

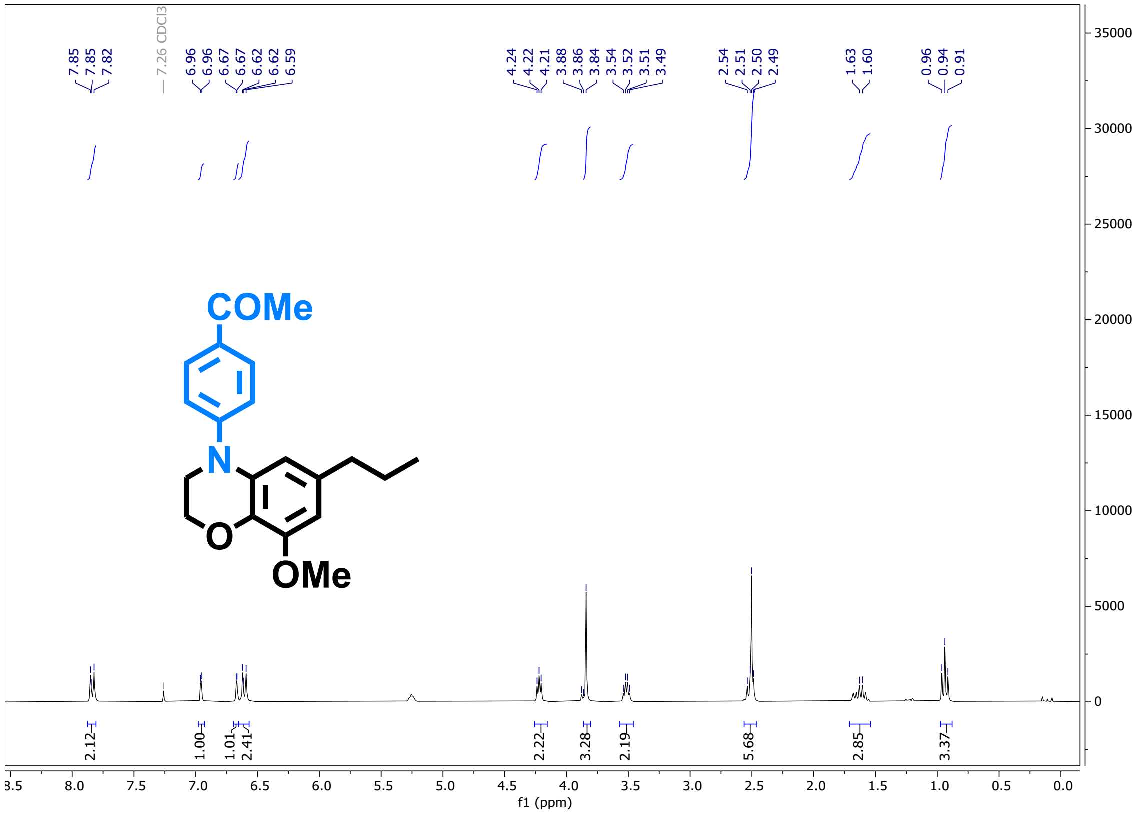

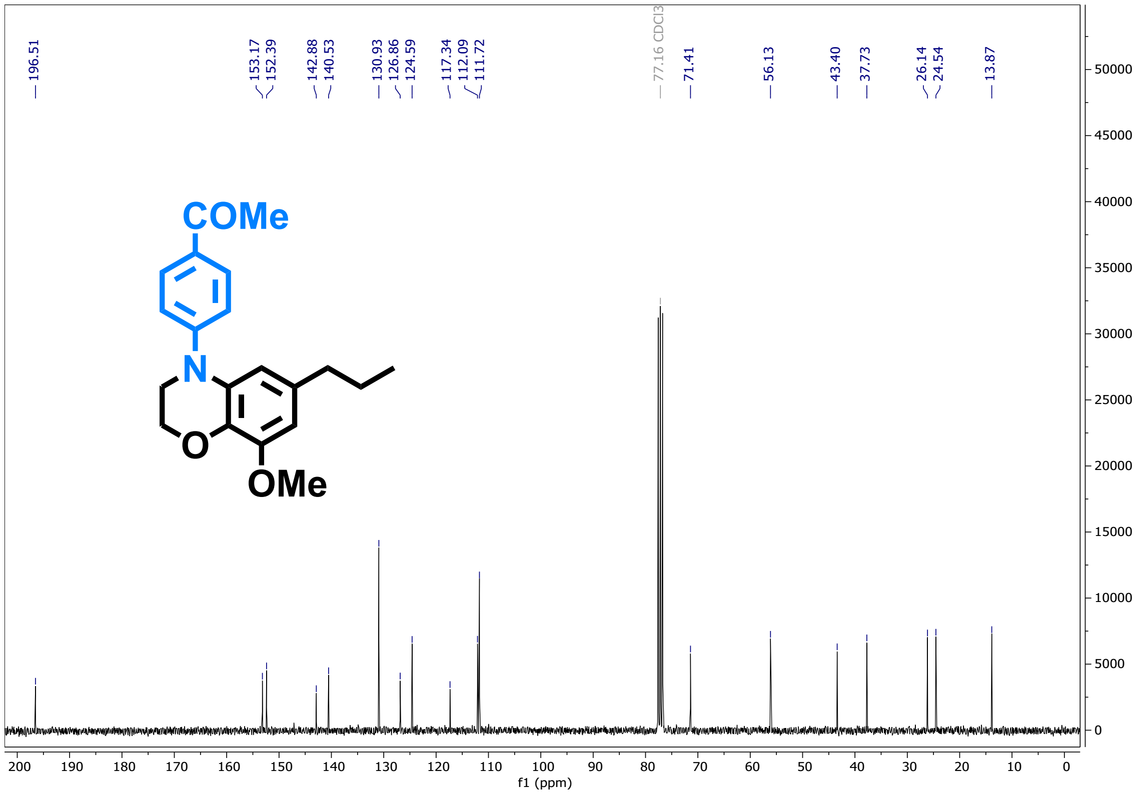

Fig. S43 ^{1}H and ^{13}C NMR spectra of compound **2Gd3**.

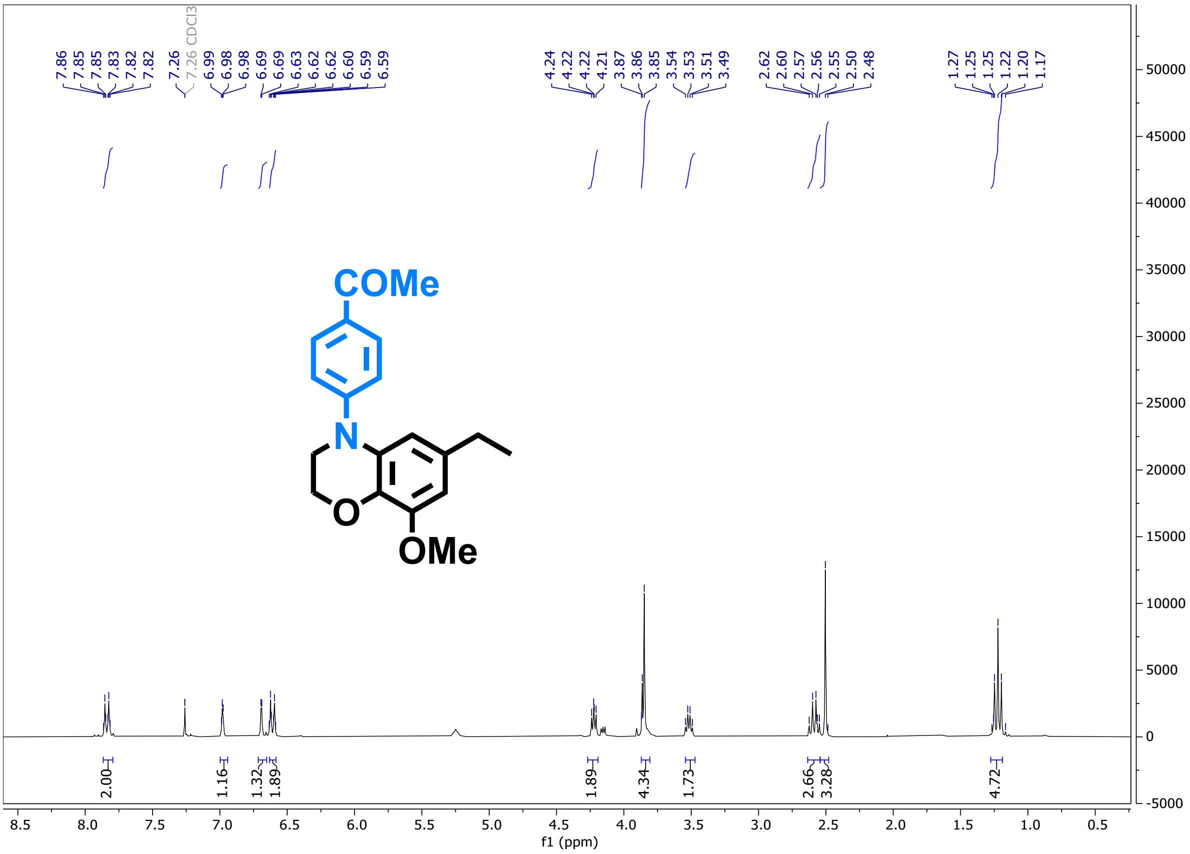
COMe
N
O
OMe
7.86
7.85
7.85
7.83
7.82
7.82
7.26
7.26 CDCl3
6.99
6.98
6.98
6.69
6.69
6.63
6.62
6.62
6.60
6.59
6.59
4.24
4.22
4.22
4.21
3.87
3.86
3.85
3.54
3.53
3.51
3.49
2.62
2.60
2.57
2.56
2.55
2.50
2.48
1.27
1.25
1.25
1.22
1.20
1.17
2.00
1.16
1.32
1.89
1.89
4.34
1.73
2.66
3.28
4.72
f1 (ppm)

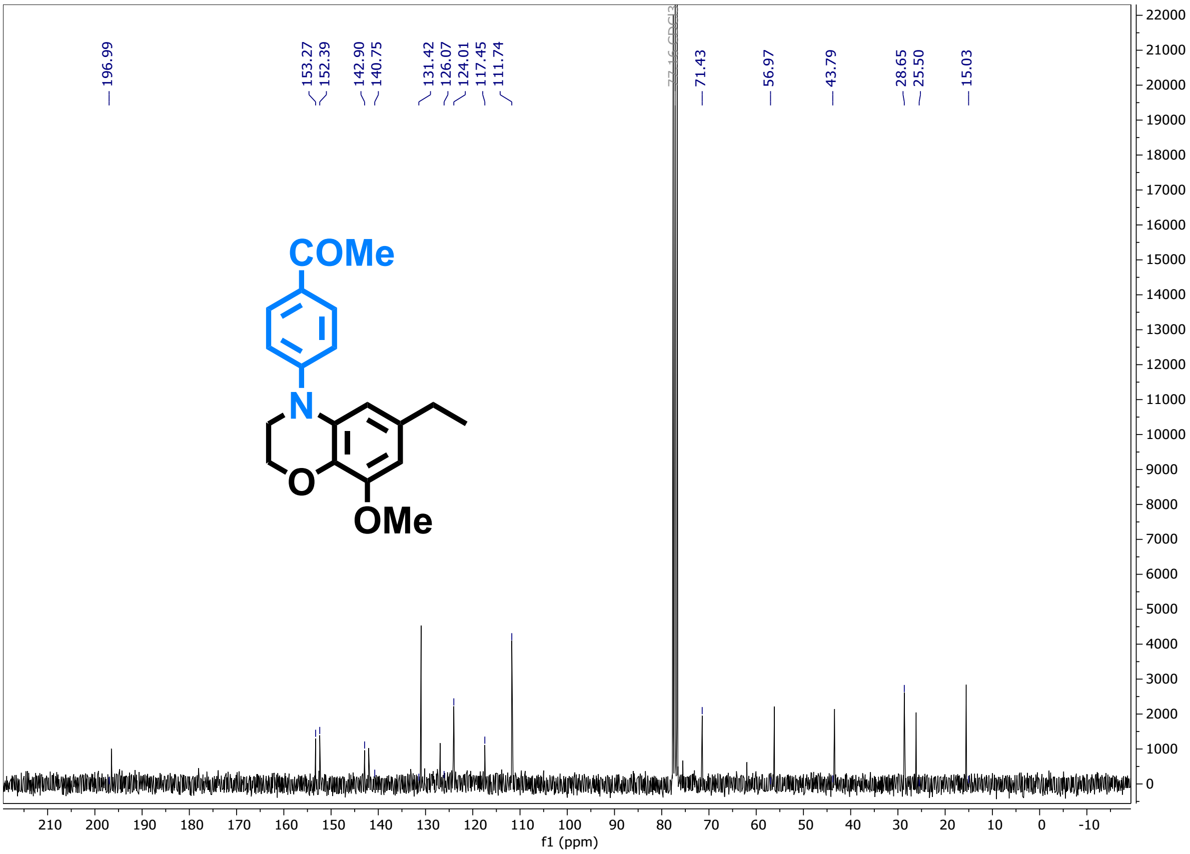

Fig. S44 ^{1}H and ^{13}C NMR spectra of compound **3Gd3**.

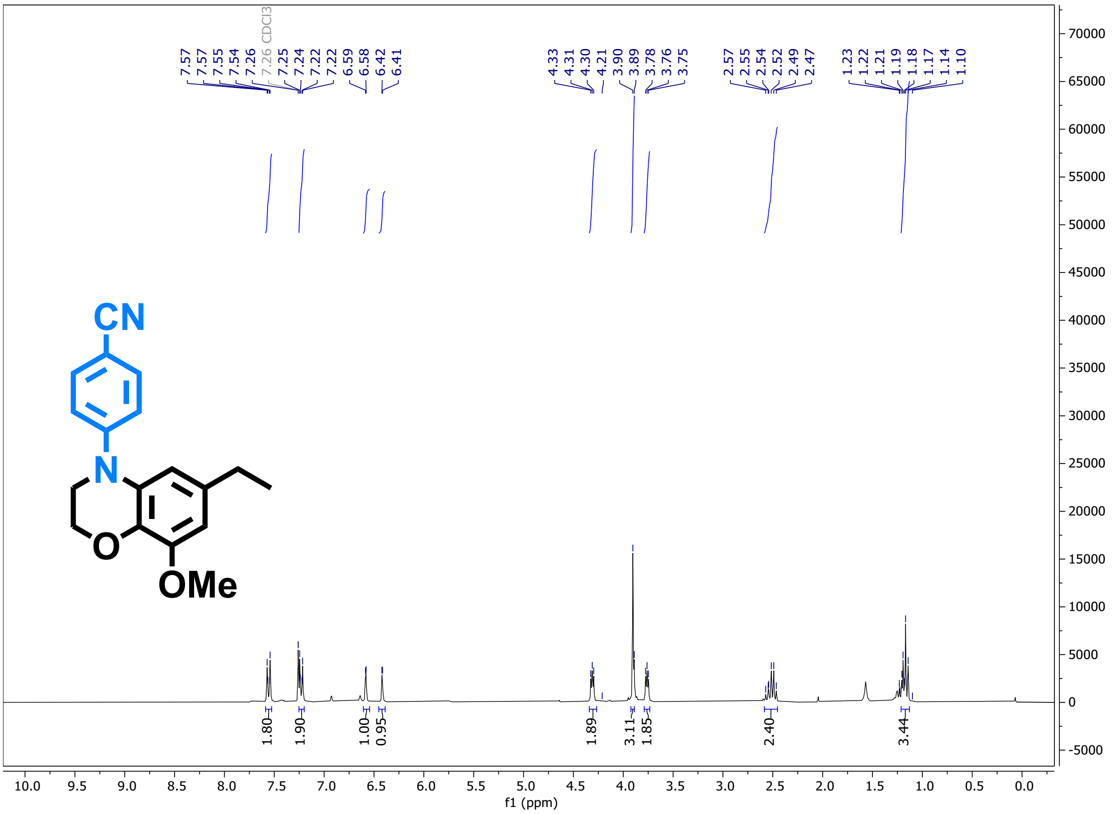

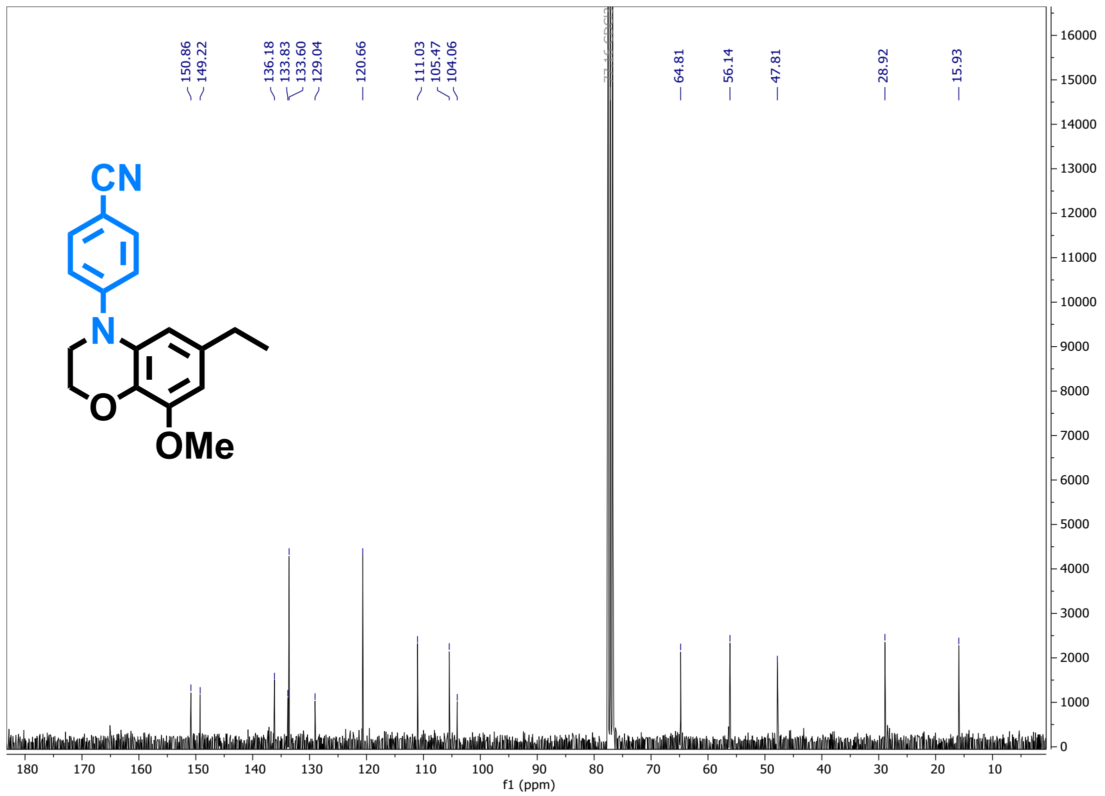

Fig. S45 ^{1}H and ^{13}C NMR spectra of compound **3Gd4**.

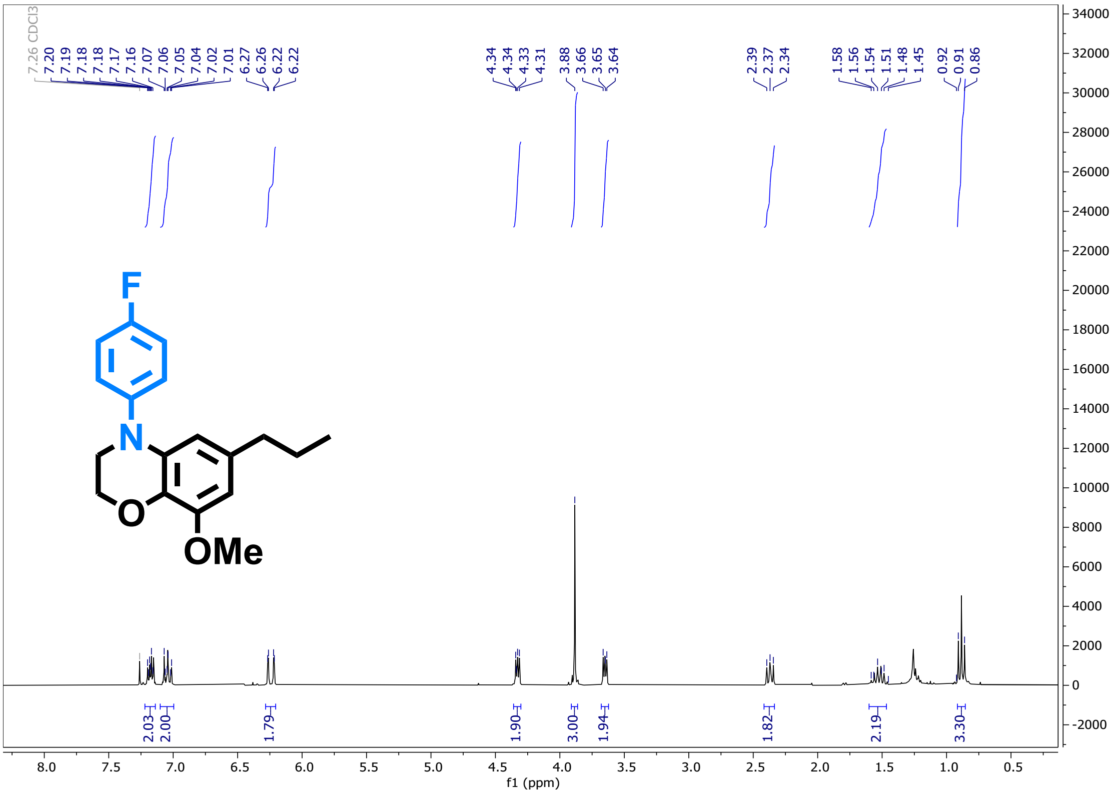

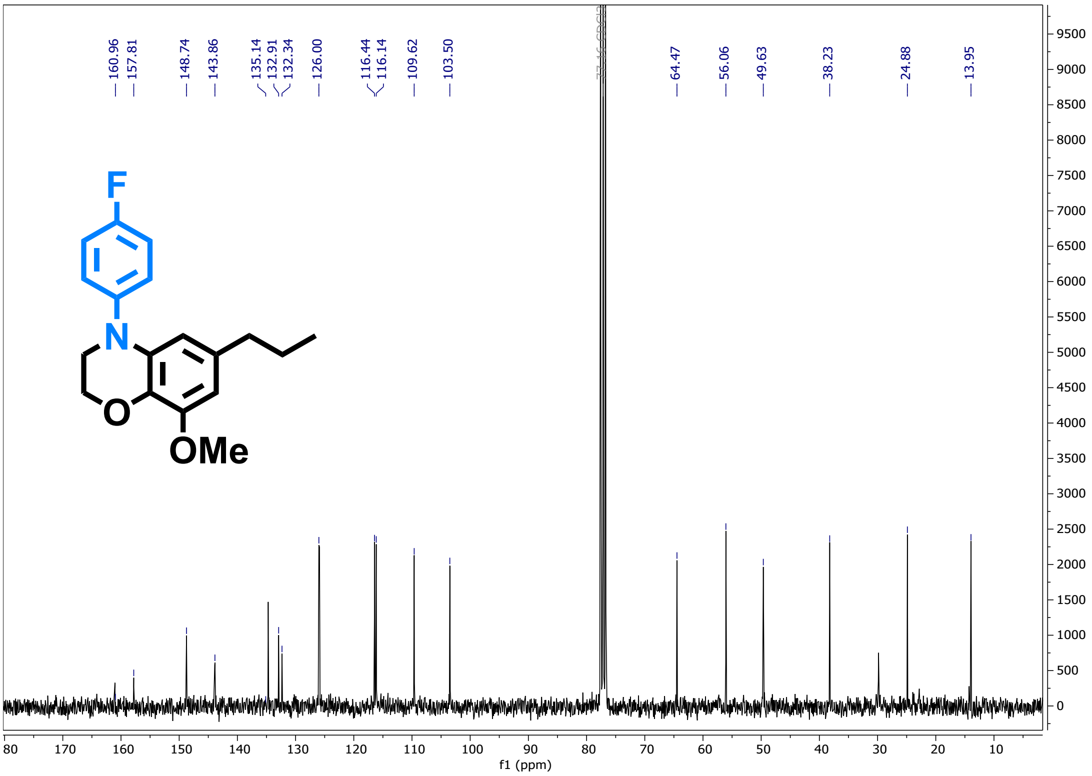

Fig. S46 ^{1}H and ^{13}C NMR spectra of compound **2Gd6**.

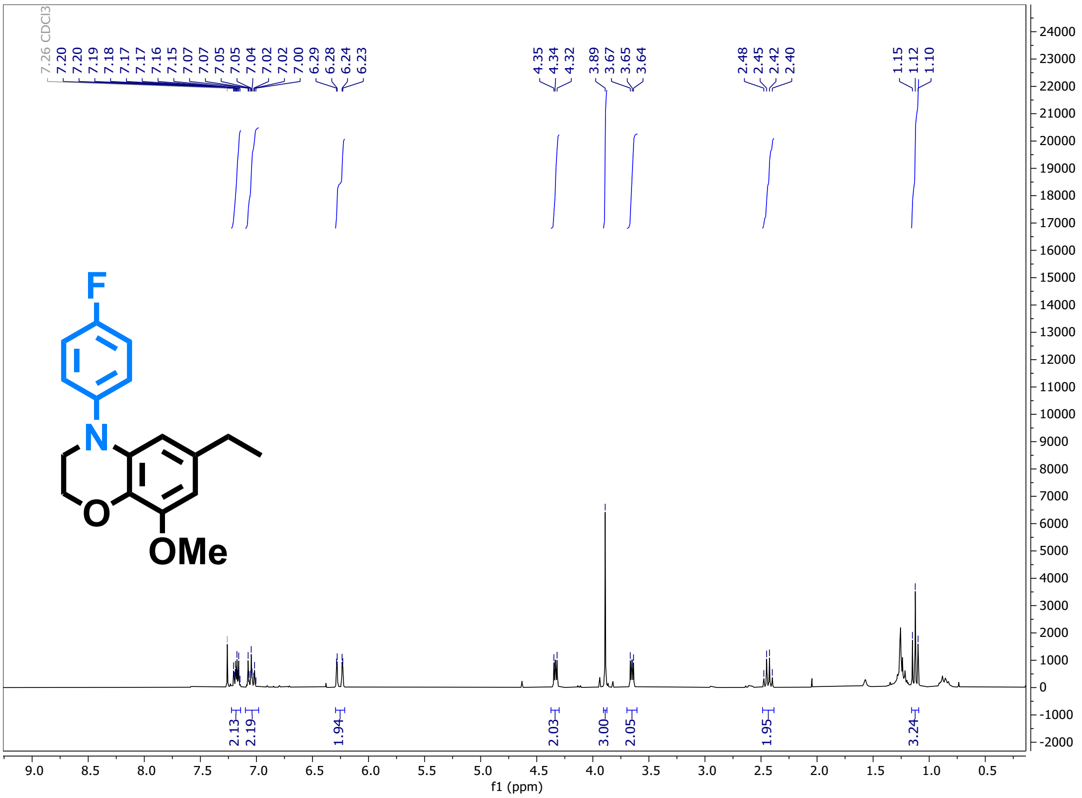

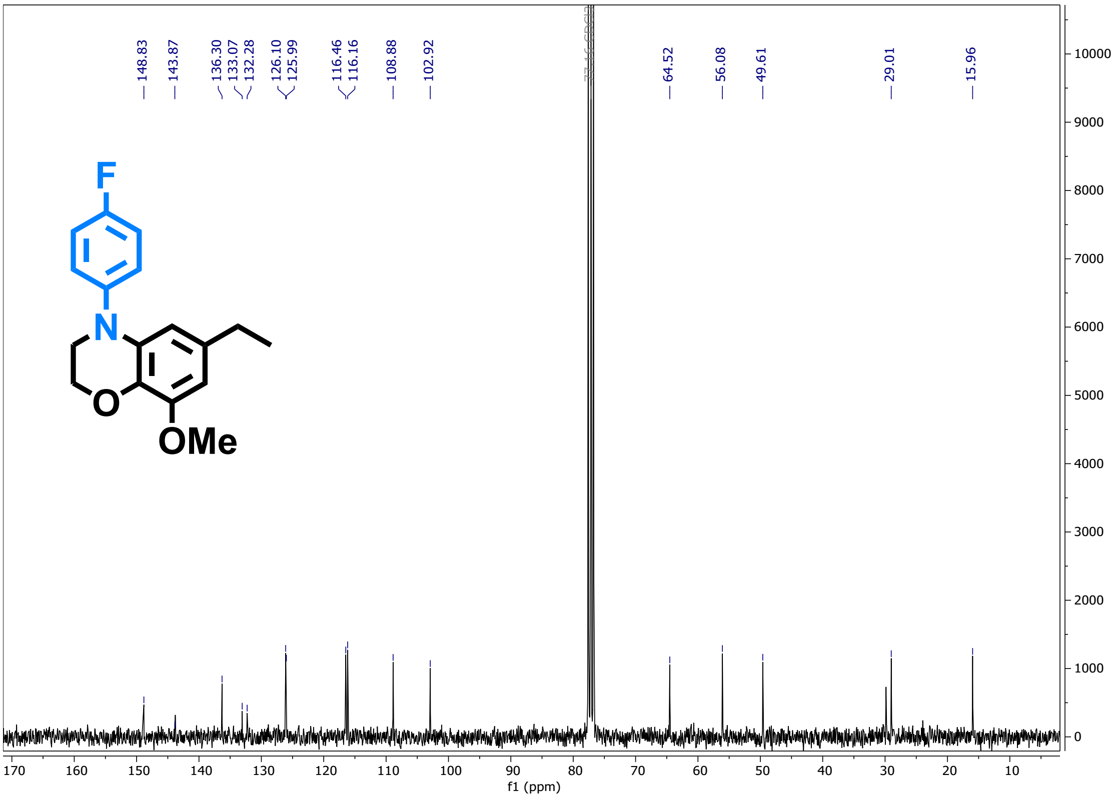

Fig. S47 ^{1}H and ^{13}C NMR spectra of compound **3Gd6**.

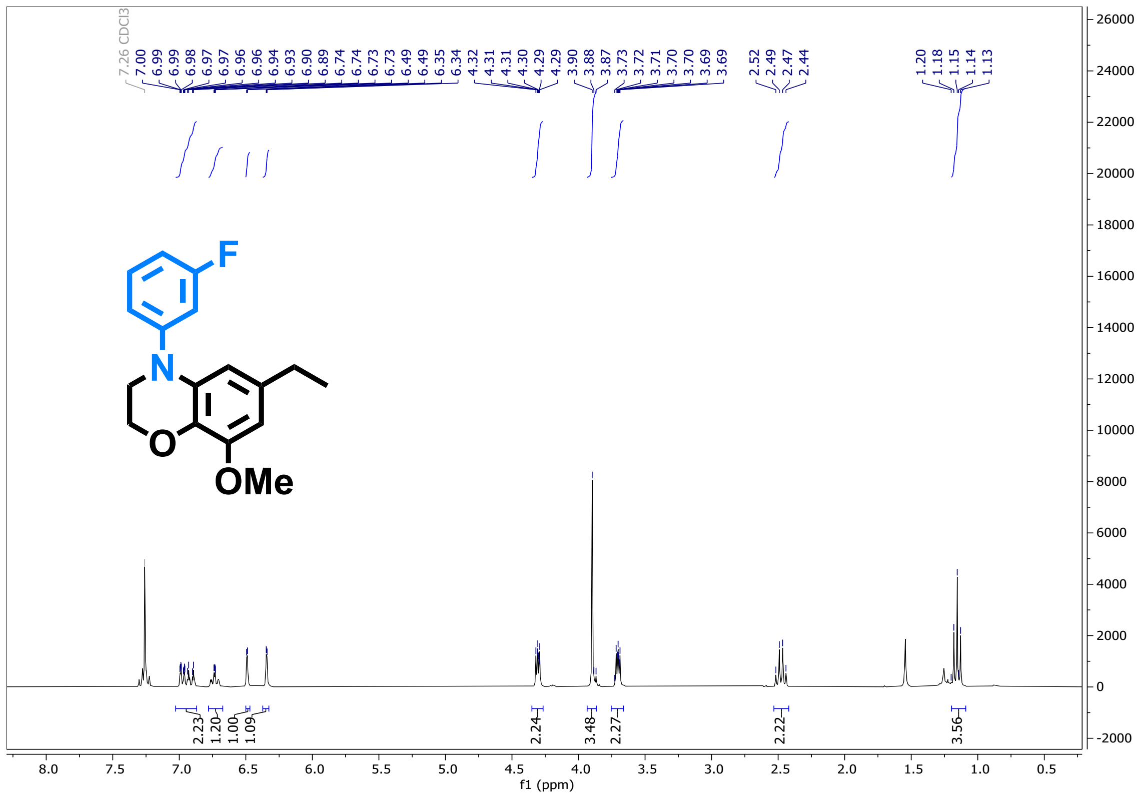

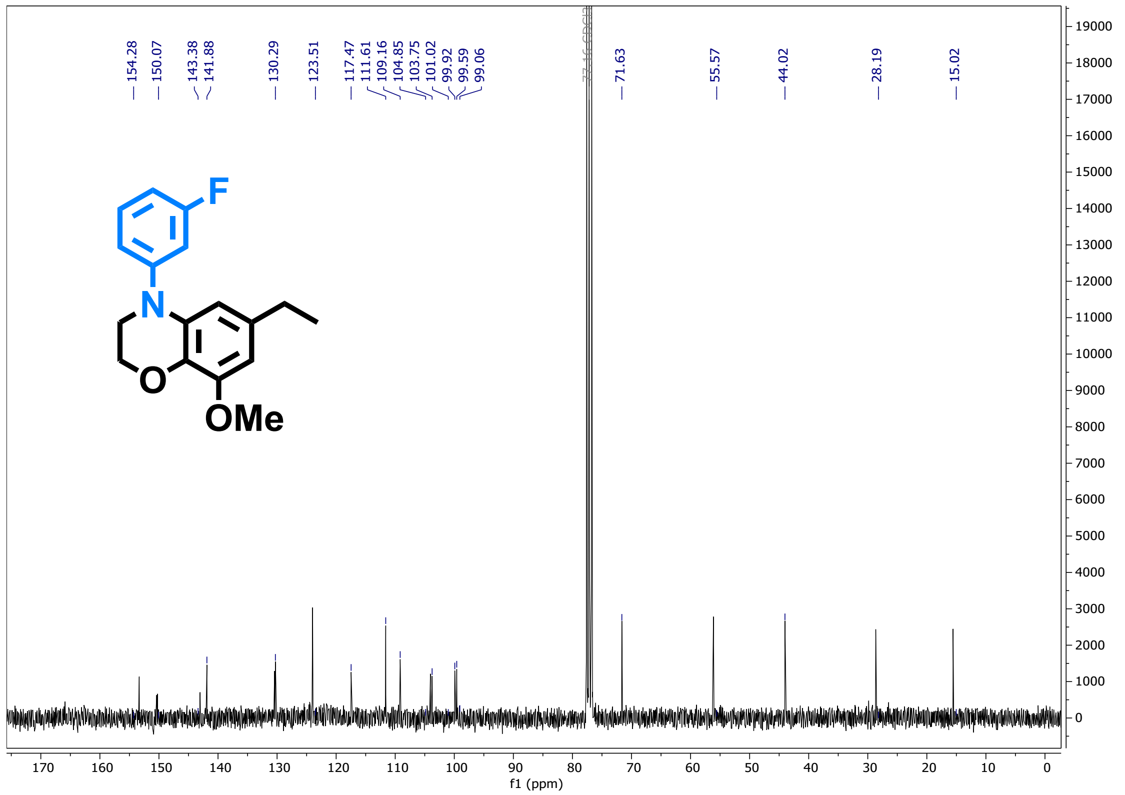

Fig. S48 ^{1}H and ^{13}C NMR spectra of compound **3Gd7**.

References

1. J. Konstantinović, A. M. Kany, A. Alhayek, A. S. Abdelsamie, A. Sikandar, K. Voos, Y. Yao, A. Andreas, R. Shafiei, B. Loretz, E. Schönauer, R. Bals, H. Brandstetter, R. W. Hartmann, C. Ducho, C.-M. Lehr, C. Beisswenger, R. Müller, K. Rox, J. Haupenthal, A. K.H. Hirsch, *ACS Cent. Sci.* 2023, **9**, 12, 2205–2215.
2. Haupenthal, J.; Baehr, C.; Zeuzem, S.; Piiper, *A. Int. J. Cancer* 2007, **121**, 206–210.
3. J. W. Bode, M. P. Doyle, M. N. Protopopova and Q. L. Zhou *J. Org. Chem.* 1996, **61**, 9146–9155.
4. T. Freese, B. Fridrich, S. Crespi, A. S. Lubbe, K. Barta and B. L. Feringa *Green Chem*. 2022 **24**, 3689–3696.
5. A. M. Afanasenko, X. Wu, A. De Santi, W. A. M Elgaher, A. M. Kany, R. Shafiei, M.-S. Schulze, T. Schulz, J. Haupenthal, A. K. H Hirsch and K. Barta, *Angew. Chem. Int. Ed.* 2023, e202308131
